# Genotypic variation in root architectural traits under contrasting phosphorus levels in Mediterranean and Indian origin lentil genotypes

**DOI:** 10.7717/peerj.12766

**Published:** 2022-03-10

**Authors:** Muraleedhar Aski, Reena Mehra, Gyan Prakash Mishra, Dharmendra Singh, Prachi Yadav, Neha Rai, Venkata Ravi Prakash Reddy, Arun Kumar MB, Renu Pandey, Madan Pal Singh, Gayacharan, Ruchi Bansal, Kuldeep Tripathi, Sripada M. Udupa, Shiv Kumar, Ashutosh Sarker, Harsh Kumar Dikshit

**Affiliations:** 1 Division of Genetics, Indian Agricultural Research Institute, New Delhi, Delhi, India; 2 International Center for Agricultural Research in the Dry Areas (ICARDA), Bhopal, Madhya Pradesh, India; 3 Seed Science and Technology, Indian Agricultural Research Institute, New Delhi, Delhi, India; 4 Plant Physiology, Indian Agricultural Research Institute, New Delhi, Delhi, India; 5 Division of Germplasm Evaluation, National Bureau of Plant Genetic Resources, New Delhi, Delhi, India; 6 International Center for Agricultural Research in the Dry Areas (ICARDA), Rabat, Morocco; 7 India International Center for Agricultural Research in the Dry Areas (ICARDA), New Delhi, Delhi, India

**Keywords:** *Lens culinaris*, Hydroponics, Adequate and deficit mediums, Root system, Phosphorus efficiency and CPEM value

## Abstract

The development of phosphorus-efficient crop cultivars boosts productivity while lowering eutrophication in the environment. It is feasible to improve the efficiency of phosphorus (P) absorption in lentils by enhancing phosphorus absorption through root architectural traits. The root architectural traits of 110 diverse lentil genotypes of Indian and Mediterranean origin were assessed, and the relationships between traits were investigated. In a hydroponics experiment, the lentil lines were examined at the seedling stage under two conditions: adequate P supply and deficient P supply. The Pearson correlation coefficients between root architectural traits and genetic diversity among lentil lines were assessed. To estimate variance components, a model (fixed factor) was used. In this experiment, both phosphorus (P) and genotype were fixed variables. Our lentil lines showed significant genetic variability and considerable genetic diversity for all traits under both treatments. The TRL (total root length) and PRL (primary root length) showed strong positive associations with all other characteristics excluding root average diameter (RAD) in both P treatments. In both P treatments, the RAD revealed a negative significant association with Total Root Tips (TRT), as well as total root volume (TRV) and total root forks (TRF) in the deficit conditions of P. Total root volume (TRV), total surface area (TSA), and total root tips had higher coefficient variance values. The first two principal components represented 67.88% and 66.19% of the overall variance in the adequate and deficit P treatments respectively. The Shannon-Weaver diversity index (H′) revealed that RAD, PRL, and TSA had more variability than TRT and TRF under both treatments. According to the Comprehensive Phosphorus Efficiency Measure (CPEM), the best five highly efficient genotypes are PLL 18-09, PLS 18-01, PLL 18-25, PLS 18-23, and PLL 18-07, while IG112131, P560206, IG334, L11-231, and PLS18-67 are highly inefficient genotypes. The above contrasting diverse lentil genotypes can be utilized to produce P-efficient lentil cultivars. The lentil germplasm with potentially favorable root traits can be suggested to evaluated for other abiotic stress to use them in crop improvement programme. The scientific breakthroughs in root trait phenotyping have improved the chances of establishing trait–allele relationships. As a result, genotype-to-phenotype connections can be predicted and verified with exceptional accuracy, making it easier to find and incorporate favourable nutrition-related genes/QTLs in to breeding programme.

## Introduction

The domesticated lentil (*Lens culinaris* Medik. ssp. *culinaris*) is a winter-loving food grain legume, having diploid (2n = 2x = 14), with a genome size of about 4,063 Mpb (Arumuganathan & Earle, 1991). Lentil is also one of the Neolithic crops of Near East origin which has been linked to the inception of agricultural activities since the days of its emergence (Ladizinsky, 1979; Yadav, McNeil & Stevenson, 2007). It is predominantly cultivated in West Asia, South Asia, North Africa, and North America. Lentils are rich in minerals, prebiotic carbohydrates, carbohydrates, dietary proteins (18–34%), and fiber.

Lentils are important in addressing micronutrient deficiencies and reducing malnutrition in underdeveloped nations (Kumar et al., 2019; Kumar et al., 2015; Siva et al., 2019). Since, lentils have a lower glycemic index; they can help with typical lifestyle issues like obesity, diabetes, and cardiovascular disorders (Srivastava & Vasishtha, 2012).

Canada, India, and Turkey are the largest global growers of lentils, accounting for almost 71% of the world’s harvest. In 2017, approximately 7.59 million tonnes (MT) of lentils were grown on 6.58 million hectares around the world (FAOSTAT, 2019). In comparison to the world average of 1,153 kg ha^−1^, India’s productivity (736 kg ha^−1^) is significantly very low (FAOSTAT, 2019; Dey et al., 2017).

Lentils have a lower productivity than other pulses because they are grown by resource-constrained farmers in rainfed agro-ecosystems in India, where little or no fertiliser is often used. Apart from that, available phosphorus (P) levels in Indian soils range from moderate to poor (Tewatia et al., 2017). In India, nutrient usage (NPK) is generally considered poor, with K and P use being especially poor (Mogollón et al., 2018).

The use of nitrogen in an extremely imbalanced manner does not boost crop yields or payback over time, and it greatly diminishes available soil P (Tewatia et al., 2017). In most Indian soils, P deficiency is common. P loss will typically outnumber net P supply, and the decrease in P will going to rise over time, limiting agricultural productivity. In 2005, the global demand for agricultural supplies of P fertilizer was 14.5 Tg P yr^−1^, with a projected demand of 22–27 Tg P yr^−1^ by 2050 (Where Tg: teragram; 1 Tg = 1012 g = 1 million metric tonnes) (Mogollón et al., 2018). The world’s top phosphate fertilizer manufacturers are the United States of America, the People’s Republic of China, and Morocco (Wu et al., 2016). Potential domestic demand has compelled the USA and China to halt rock phosphate exports to the rest of the world (van de Wiel, van der Linden & Scholten, 2016). A sustainable plan for the potential use of P becomes focused on a projected world’s population of around 9.7 billion people and sufficient agricultural output in 2050 (Abel et al., 2016).

Phosphorus (P) is important for the growth and development of plants, the production of DNA, the production of cell membranes, and is vital for all forms of life. It performs a variety of essential functions, including photosynthesis, glycolysis, synthesis of DNA, respiration, ATP synthesis, synthesis of the cell membrane, redox reactions, sugar, and starch transformation, energy transfer, inactivation/activation of enzymes, movement of nutrients, metabolism of carbohydrates, inheritance of traits, signaling, and fixation of nitrogen (Malhotra, Sharma & Pandey, 2018; Abel, Ticconi & Delatorre, 2002; Rychter & Rao, 2005).

P deficiency has a much greater impact on the shoot than it does on the root, contributing to a higher root: shoot ratio (RSR) (Pandey, 2015). In crops, a lack of P causes darker green foliage, reddish-purple tips, and stunted growth, all of which interfere in the electron transport mechanism by decreasing orthophosphate aggregation in the stroma of the chloroplast, ATP synthase hindrance, and photosynthesis breakdown (Chen et al., 2014; Carstensen et al., 2018). Since P is attached or fixed to Ca in alkaline soils and iron and aluminum in acidic soils, the process of soil phosphorus uptake is quite challenging (Heuer et al., 2017). According to some figures, there is a P deficiency of around 5.7 billion ha, and P levels in soil solutions were well below critical thresholds (0.1 to 10 mM) (Hinsinger, 2001).

Enhancements in either P uptake or P utilization will increase the phosphorus use efficiency (PUE) of crops (Valle, Pinochet & Calderini, 2011; Sandaña & Pinochet, 2014). In chickpea (Keneni et al., 2015), mungbean (Irfan, Shah & Abbas, 2017; Reddy et al., 2020a, 2020b), field pea (Powers & Thavarajah, 2019), pigeonpea (Sidhu, Kaur & Singh, 2017), cowpea (Abaidoo et al., 2017), soybean (Krishnapriya & Pandey, 2016), brassica (Hu et al., 2010), sorghum (Leiser et al., 2014), pearl millet (Faye et al., 2006), maize (Zhang et al., 2014), rice (Wissuwa et al., 2015), and wheat (Hari-Gowthem et al., 2019), the PUE and its component traits showed higher genetic variation. The modification of root architecture characteristics aims to explore soil space, and thus improved interplay between root and soil improves phosphorus uptake from soil.

(Panigrahy, Rao & Sarla, 2009; Stangoulis, 2019; Lynch, 2011). The rate of nutrient uptake by the root system is determined by the specific nutrient levels on the root surface area, morphology of root, and the crop’s needs (Adler, Cumming & Arora, 2004). The root is a crucial plant organ for nutrient and water uptake and by increasing root surface area (RSA) and thus exposing a larger amount of soil available nutrients (Hodge et al., 2009).

Plants change their root architectural characteristics in low-phosphorus environments (Hodge et al., 2009; Hodge, 2004), including decreased growth in primary roots, increased length and number of root-hairs and lateral roots (López-Bucio, Cruz-Ramırez & Herrera-Estrella, 2003; Pérez-Torres et al., 2008; Akhtar, Oki & Adachi, 2008), enhanced root volume and surface area (Jakkeral, Kajjidoni & Koti, 2009), a shallow angle of growing roots (Zhu, Kaeppler & Lynch, 2005), and an enhanced in root biomass (Hermans et al., 2006). Genetic diversity in root morphological traits can also be used to enhance the availability of water and nutrient quality under difficult growing conditions (Malamy & Benfey, 1997).

The P-efficient genotypes in mungbean had substantially longer roots length, higher surface area, biomass, and carboxylate exudation ability of roots, compared with inefficient genotypes (Pandey et al., 2014). In the case of black gram 45 days after sowing, the P uptake was significantly influenced by root surface area, root weight, root volume, and lateral root number (Jakkeral, Kajjidoni & Koti, 2009). During 8 days of exposure, deficit phosphorus condition causes a significant rise in total root length, primary root length, and the number of lateral roots in lentils (Sarker & Karmoker, 2009). Large adventitious and densities of lateral roots have been linked to higher soybean and common bean uptake of P per unit length (He et al., 2017; Borch et al., 1999).

Genotypes having a broad root system and deeper lateral roots outperformed genotypes with a medium and narrow root system of lupine in terms of yield and P efficiency of roots (Chen et al., 2013). In rice, in all genotypes tested against low P constraints, root density and root hair length enhanced significantly (Vejchasarn, Lynch & Brown, 2016). In the case of wheat, root biomass and root length maintenance are vital to cope against P deficiency (Shen et al., 2018). Plants with fibrous roots (maize, wheat, brassica) had a greater reduction in root length in low P conditions than legumes (chickpea, soybean, lupin, and broad bean). There was a greater root-to-shoot ratio for maize and wheat and brassica had a better root-specific length than that of legumes (Lyu et al., 2016). Even though P deficiency can affect crops during the growing season, the high-performance and low-cost method of phenotypic seedling evaluation save time and space (Meeks et al., 2013). The theory of stress gradients (Kitajima & Fenner, 2000; De La Cruz et al., 2008) shows that the seedlings’ condition affects the plant population structure and dynamics. The new digital image processing makes the plant root system accurate and cost-effective yet labor-saving study simpler (Bouma, Nielsen & Koutstaal, 2000; Himmelbauer, Loiskandl & Kastanek, 2004).

This research aimed to (i) describe the root trait variability in lentil under P deficit and adequate conditions, (ii) determine the root traits that contributed to the majority of the variation in lentil, and (iii) compare the performance of 110 lentil genotypes and identifying the best responsive genotypes based on root trait architecture and CPEM values under adequate and deficient P situations respectively.

## Materials and Methods

### Experimental material and growth conditions

The root architectural traits of 110 lentil genotypes (including 42 exotic lines of germplasm), 49 ABL (advanced breeding lines), 11 Indian origin lentil germplasm lines, and eight commercially released varieties (Table S1) were assessed using a completely randomised design (CRD) in a concentration of 250 m KH_2_PO_4_ for sufficient P treatment and 3.0 M KH_2_PO_4_ for deficit P treatment. During November 2018 and October 2019, the investigation was carried out in controlled environment chambers at the National Climate Resilient Agriculture Initiative (NICRA) at the Indian Agricultural Research Institute (IARI), New Delhi, India. The chamber room air temperature at 23/18 °C and a relative humidity of about 45 percent (±2%), was maintained.

The light intensity was sustained around 450 and 1,310 μmol m^−2^ s^−1^. Before being put for germination, lentil seeds were sterilized and washed three times with double-distilled water over three and a half minutes with 0.1 percent HgCl_2_ (w/v) on the surface. At the two leaves of the cotyledonary stage, uniform seedlings with no root anomalies were transplanted to Hoagland solution. In compliance with (Sivasakthi et al., 2017), macro and micronutrients were included in the nutrient solution. Preliminary research was conducted to evaluate adequate and deficient P levels on the grounds of biomass production, visual symptoms, and chlorophyll content. KH_2_PO_4_ was used to maintain two levels of P (sufficient, P 250 M, and deficit, P 3 M). The pH of the nutrient solution can be adjusted to 6.0 by slowly adding 1 M KOH or 1 M HCl solution. In this plastic container (experimental unit or hydroponics tray) with five seedlings per genotypes were screened and three uniform seedlings constituted three biological replicates. The eight genotypes were evaluated in an experimental unit at time. The aquarium air pump (two way) with a 5 W capacity has oxidised the solution, and the solutions needs to be replaced before the fourth day. ICAR-IARI New Delhi is having seed materials. They can be accessed following the Indian Government’s National Biodiversity Agency’s regulations and laws.

### Measurements on roots

The root parameters of seedlings grown in adequate and deficient P at the age of 24 days were measured. Each plant’s entire root structure was carefully extracted and spread out on a scanning tray, with no overlapping roots. The roots were investigated using a root scanner Epson (model: V700, EPSON™). The grayscale image quality of TIFF files was analyzed by WinRhizo™.

Settings specifications: 400 dpi image resolution, white background with manual dark root color, scanner calibration, depth of eight-bit, 4,395 × 6,125-pixel image resolution, and focal length of 0 mm. The roots were placed in an acrylic tray (30 × 40 × 2 cm) filled with 700 ml of water. By floating the root specimen in a flask of water, the debris was manually removed. Cleaner roots, which were trash-free, were used for analysis and scanning. Total root length (TRL), primary root length (PRL), root average diameter (RAD), total root crossings (TRC), total surface area (TSA), total root volume (TRV), total root forks (TRF), and total root tips (TRT) were the root characteristics studied (Table S2).

The WinRhizo™ also produced new output files that allow us to categorize root features into five groups namely (1) 0 to 0.5 mm, (2) 0.5 to 1.0 mm, (3) 1.0 to 1.5 mm, (4) 1.5 to 2.0 mm, and (5) >2.0 mm, based on root diameter intervals in total root tips (TRT), total root volume (TRV), and root surface area (RSA) (Li et al., 2015; Gorim & Vandenberg, 2017; Liu et al., 2018). The percentage of root characteristics in each class was determined as a (%) percentage of total traits (Liu et al., 2018). Every class’s percentage of root trait was computed as a percentage of total traits (Liu et al., 2018).

### Statistical analysis

The STAR 2.1.0 (Statistical Tool for Agricultural Research) software was used to analyze data and calculate descriptive statistics such as standard deviation, mean, ANOVA (analysis of variation), coefficient of variation (CV), and Pearson’s correlation coefficients, for tested characteristics in sufficient and deficit P conditions (Gulles et al., 2014). The broad sense heritability (H^2^) were calculated using a SAS (SAS Institute Inc, 2009) program as previously described by (Schmidt et al., 2019). The fixed model was used for the analysis of root trait variance. Lentil genotype and Phosphorus are fixed factors in the fixed factor model. The additive linear model is as follows:

$Y_{ijk} = {\mu} + G_{i} + P_{i} + (GP)_{ij} + E_{ijk}$

In the equation above, *Y*_*ijk*_ represents the finding from the *k*^*th*^ replication of the experimental unit *(ij*^*th*^*)*, the overall mean is represented by *µ*, *G*_*i*_ represents the primary impact of the lentil line *(i*^*th*^*)*, *Pj* represents the primary effects of the *j*^*th*^ P stage, *GP*_*ij*_ represents the lentil line -P interaction effect, and the error of random effects perplexed by *E*_*ijk*_.

Lentil genotypes (110) were classified into three classes based on the data collected: (1) (x – SD) lentil entries with low performance, (2) (x – SD) medium-performing genotypes to (x + SD), (3) (x + SD) lentil entries high-performance, where SD and x refer to the standard deviation and mean of respective root characters (Abdel-Ghani et al., 2012; Zar, 2010). For each trait, the Shannon-Weaver Diversity Index (H818c) (Shannon & Weaver, 1963), a polymorphic diversity index, was computed using the equation:

$H^\prime=-\mathop \sum \limits_{i = 1}^{\rm s} pi\; \left( {{\rm ln}pi} \right)$

In which, *pi* denotes the portion of lines in the class (*i*^*th*^*)*, and the genotypes total unit was denoted by *S*.

Using the STAR 2.1.0 program, PCA (principal components analysis) was used to classify characteristics that contributed the most variation in the evaluated lentil genotypes. The efficiency potential of all lentil entries studied was assessed using a CPEM value (comprehensive P efficiency measurement). The following formulas were used to measure the CPEM value (Li et al., 2015; Xu, Zhang & Xiang, 2009). The PEC-P efficiency coefficient was computed as the proportion of the variables estimated from the phosphorus deficit (DP) and phosphorus sufficient (SP) treatments of the very same lentil line for each characteristic utilizing the below formula.

$PEC_{ij} = X_{ijDP}/X_{ijSP}$

where phosphorus efficiency coefficient is represented by *PEC*_*ij*_ of the characteristics (*j*) of the lentil line (*i*); *X*_*ijDP*_ and *X*_*ijSP*_ are the estimates of the characteristics (*j*) of the lentil line (*i*) tested in deficit P (DP) and sufficient P (SP) conditions.

The fuzzy subordination approach must be used to fully evaluate the P efficiency and prevent a single index shortage. A fuzzy set’s membership function is a generalization of a classical set’s indicator function, representing the amount of truth in a valuation extension (Li et al., 2015; Zadeh, 1965; Chen et al., 2012). The membership function value of phosphorus efficiency (MFVP), abbreviated as *U*_*ij*_, suggests a favorable relationship between traits and P efficiency.

$Uij = \displaystyle{{PECij - PECjmin} \over {PECjmax - PECjmin}}\quad \rm (j = 1,2,3 \ldots n)$

*U*_*ij*_ represents the value of membership function in characteristics (*j*) in the genotype (*i*) of P efficiency; the highest value of the P efficiency coefficient in characteristics is *PEC*_*jmax*_ (*j*); *PEC*_*jmin*_ means the lowest of *PEC*_*j*_.

The below formula was used to calculate the total P efficiency (in terms of CPEM):

${\rm P} = \mathop \sum \limits_{j = 1}^n [Uij \times \left| {PECij} \right|/\mathop \sum \limits_{j = 1}^n \left| {PECij} \right|]\quad \rm (Where\; j = 1,2,3,4,5,\ldots n)$

Here CPEM denotes the comprehensive P efficiency calculation of every lentil genotype in the low or deficit P. Based on their CPEM values, all lentil entries were grouped into five classes: (i) Highly Efficient lines, (ii) Efficient lines, (iii) Moderately Efficient lines, (iv) Inefficient lines and (v) Highly Inefficient lines.

## Results

### Influence of phosphorus stress on root architectural characteristics

**Figure 1:**
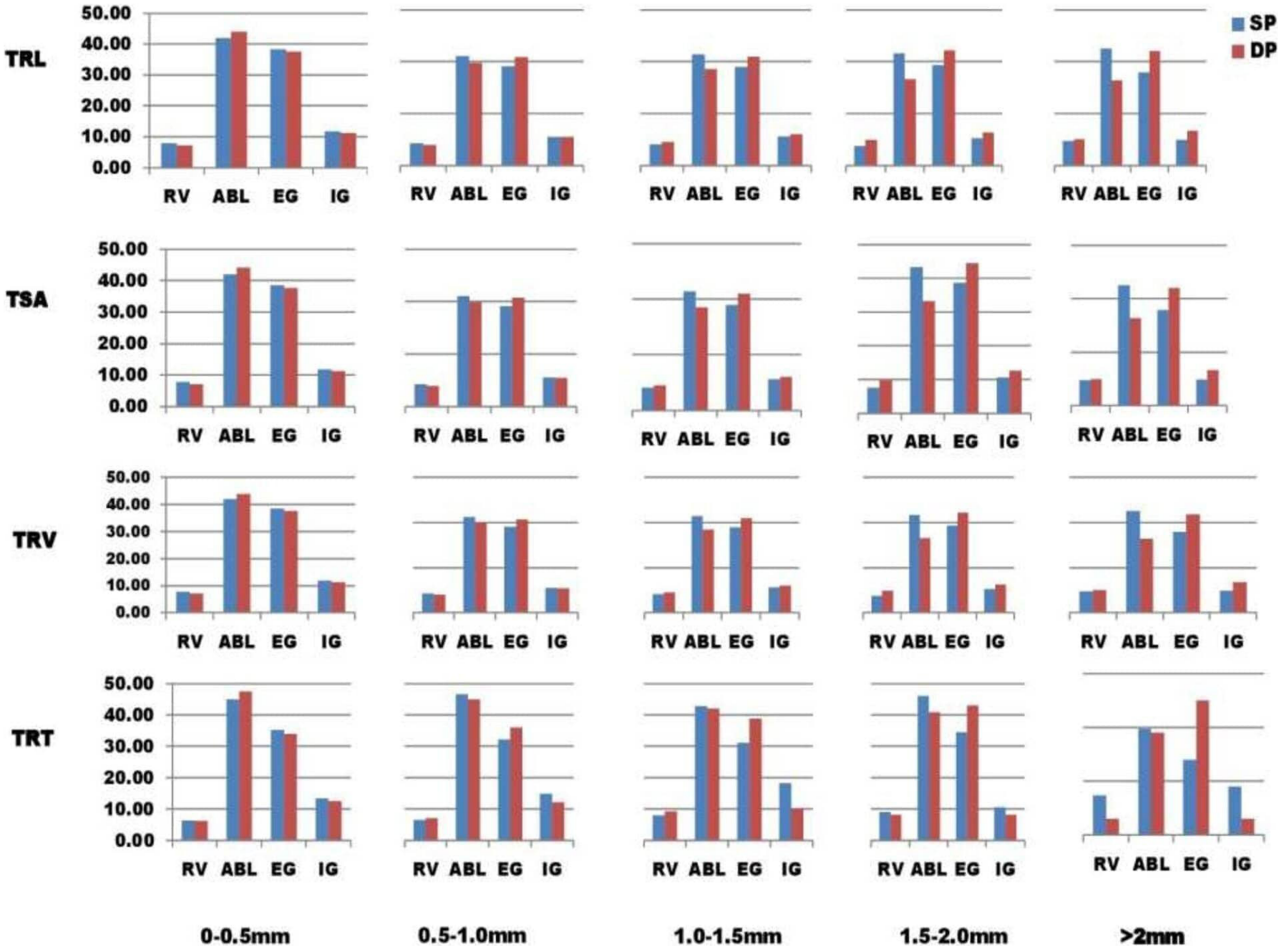
The graphical representation of frequency distribution in seven key root traits.

There was a lot of variability in the mean for the samples tested in the present research of 110 lentil lines for root architectural traits under SP and DP treatments (Table 1). An independent t-test showed that the mean values of all traits (*p*-value 0.05) were significantly higher in DP than in SP, except the TRV and TRF. The PEC mean value (phosphorus efficiency coefficient) for PRL was the highest, preceded by RAD and TRT. Average TRL, RSA, TRV, and TRT values in five root diameter groups ((a) 0 to 0.5 mm, (b) 0.5 to 1.0 mm, (c) 1.0 to 1.5 mm, (d) 1.5 to 2.0 mm, (e) more than 2.0 mm) were found to vary in SP and DP levels (Fig. 1). PEC values greater than 1 were found in TRL, RSA, and TRV for root diameters 0 to 0.5 mm, 1.5 to 2.0 mm, and >2.0 mm, except for 0.5 to 1.0 mm, 1.5 to 2.0 mm, and >2.0 mm; PEC values greater than 1 were also found in TRT for root diameters 0–0.5 mm, 1.0 to 1.5 mm, except for 0.5 to 1.0 mm, 1.5 to 2.0 mm, and >2.0 mm. At root diameters of 0–0.5 mm, 1.5 to 2.0 mm, and >2.0 mm, the PEC was greater than 1.0, implying an enhancement in root volume under the DP scenario (Fig. 1). In the 0 to 0.5 mm root diameter class, the TRT was higher under the DP.

**Table 1:** The trait-wise average value, Phosphorus Efficiency-Coefficient (PEC) value, and standard deviation (SD) under two phosphorus regimes. Except for TRV and TRF characteristics, all average values in the DP situation were significantly higher than in the SP situation, according to an independent t-test. DP, Deficit phosphorus; SP, sufficient phosphorus; TRL, total root length; PRL, primary root length; RAD, root average diameter; total root surface area (TSA); TRF, total root forks; TRT, total root tips; TRV, total root volume; TRL _1–5_, RSA _1–5_, TRV _1–5_, TRT _1–5_ indicate total root length, root surface area, root volume and root tips in diameter between (1) 0.0 to 0.5 mm, (2) 0.5 to 1.0 mm, (3) 1.0 to 1.5 mm, (4) 1.5 to 2 mm and (5) greater than 2.0 mm.

S. No.	Traits	Average ± SD (SP)	Average ± SD (DP)	Average ± SD (PEC)
1	PRL	19.79 ± 4.75	32.60 ± 5.51	1.71 ± 0.37
2	TRL	423.45 ± 199.37	292.91 ± 167.31	0.68 ± 0.16
3	TSA	47.62 ± 22.69	36.49 ± 18.56	0.87 ± 0.51
4	RAD	0.35 ± 0.07	0.46 ± 0.06	1.32 ± 0.21
5	TRV	0.52 ± 0.22	0.47 ± 0.31	1.04 ± 0.76
6	TRT	355.09 ± 276.05	293.95 ± 192.14	1.16 ± 1.02
7	TRF	701.59 ± 395.40	613.17 ± 571.73	1.08 ± 1.17
8	TRL _1_	237.5413 ± 106.72	202.02 ± 88.27	1.04 ± 0.87
9	TRL _2_	62.61 ± 25.66	51.00 ± 24.74	0.95 ± 0.68
10	TRL _3_	9.86 ± 4.13	7.59 ± 3.94	0.96 ± 0.96
11	TRL _4_	4.27 ± 2.31	2.81 ± 1.85	1.11 ± 1.87
12	TRL _5_	1.79 ± 1.23	1.10 ± 0.87	1.02 ± 1.25
13	TSA _1_	21.89 ± 10.05	18.56 ± 8.37	1.04 ± 0.87
14	TSA _2_	12.61 ± 5.06	10.25 ± 4.89	0.95 ± 0.66
15	TSA _3_	3.73 ± 1.56	2.86 ± 1.49	0.96 ± 0.99
16	TSA _4_	2.30 ± 1.25	1.51 ± 1.00	1.10 ± 1.82
17	TSA _5_	1.26 ± 0.87	0.77 ± 0.61	1.03 ± 1.28
18	TRV _1_	0.18 ± 0.08	0.15 ± 0.07	1.05 ± 0.88
19	TRV _2_	0.21 ± 0.08	0.17 ± 0.08	0.94 ± 0.64
20	TRV _3_	0.11 ± 0.05	0.09 ± 0.05	0.96 ± 1.02
21	TRV _4_	0.010 ± 0.05	0.05 ± 0.04	1.09 ± 1.78
22	TRV _5_	0.07 ± 0.05	0.04 ± 0.03	1.04 ± 1.32
23	TRT _1_	408.04 ± 281.68	323.38 ± 247.06	1.16 ± 1.56
24	TRT _2_	9.50 ± 7.75	6.46 ± 3.45	0.95 ± 0.78
25	TRT _3_	0.95 ± 0.77	0.95 ± 0.71	1.20 ± 1.53
26	TRT _4_	0.47 ± 0.43	0.41 ± 0.41	0.65 ± 0.97
27	TRT _5_	0.18 ± 0.26	0.15 ± 0.24	0.15 ± 0.42

### ANOVA-analysis of variance and (H^2^) heritability (Broad sense)

**Figure 2:**
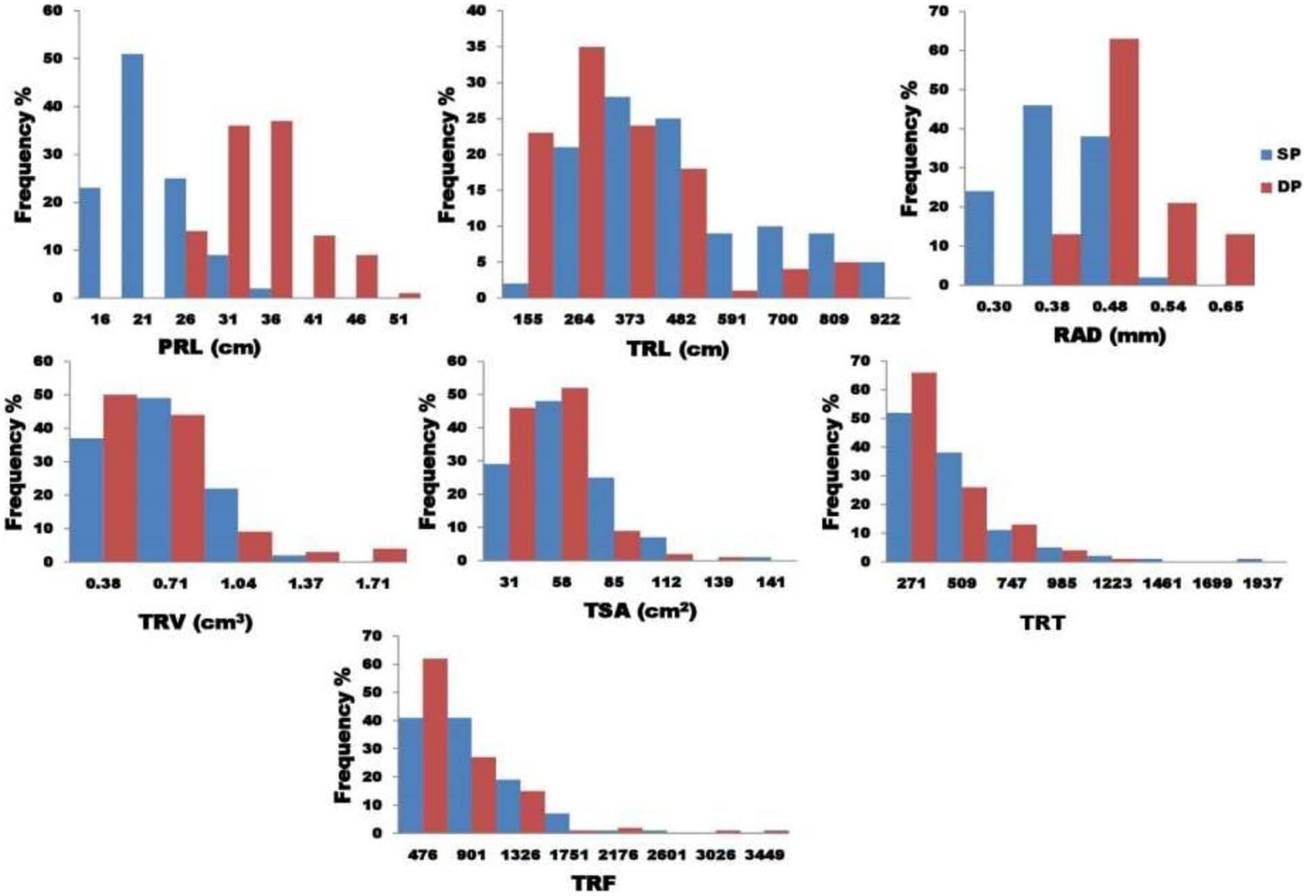
The graphical representation frequency distribution in TRL, TSA, TRV, and TRT among five diameter classes in two phosphorus conditions.

Analysis of variance found that seven traits (PRL, TSA, TRL, RAD, TRT, TRV, and TRF) examined in two P treatments showed highly significant variance (Table 2). The findings show a strong interaction between genotype and treatment, implying that the studied root characteristics differed significantly between the two P treatments. Figure 2 depicts the variability for the P efficiency in the seven root characteristics studied. A histogram of the frequency distribution showed an almost normal root distribution. For the seven traits tested, the coefficient of variation ranged from 6.76 (RAD) to 13.67 (TSA). For the traits tested, broad-sense heritability (H^2^) extended from 0.25 (TRF) to 0.97 (TRL).

**Table 2:** ANOVA of various root characteristics in two phosphorus conditions.

S. No.	Traits	Mean squares (F value)						
		Genotype (G)	Phosphorus (P)	G × P	Error	Pr (>F)	CV%	Heritability
	**df**	109	1	109	440			
1	PRL	125.42 (31.91)	27,097.64 (6,894.54)	33.60 (8.55)	3.930	0.000	7.57	0.86
2	TRL	1,90,947.24 (135.70)	2,811,610.99 (1,998.12)	12,273.62 (8.72)	1,407.12	0.000	10.47	0.97
3	TSA	1,921.22 (58.10)	20,439.02 (618.11)	656.15 (19.84)	33.067	0.000	13.67	0.80
4	RAD	0.02 (25.47)	1.81 (2,389.47)	0.01 (6.93)	0.0008	0.000	6.76	0.86
5	TRV	0.26 (72.42)	0.37 (103.75)	0.18 (49.41)	0.0036	0.000	12.07	0.49
6	TRT	2,11,168.36 (225.10)	6,15,429.87 (656.05)	1,28,192.26 (136.65)	938.089	0.000	9.44	0.57
7	TRF	7,79,889.73 (128.83)	1,290,109.70 (213.11)	6,69,750.29 (110.63)	6,053.69	0.000	11.84	0.25
8	TRL _1_	34,400.55 (2.45)	2,08,190.89 (14.81)	23,140.67 (1.65)	1,453.86	0.000	53.94	0.62
9	TRL _2_	2,128.32 (1.97)	22,219.92 (20.58)	1,683.39 (1.56)	1,079.89	0.000	57.84	0.48
10	TRL _3_	61.40 (1.70)	852.55 (23.60)	36.30 (1.01)	36.12	0.000	68.89	0.80
11	TRL _4_	17.31 (2.10)	353.10 (42.87)	8.95 (1.09)	8.236	0.000	81.13	0.84
12	TRL _5_	4.28 (2.45)	77.44 (44.45)	2.49 (1.43)	1.742	0.000	91.37	0.79
13	TSA _1_	311.26 (2.63)	1,830.40 (15.49)	202.30 (1.71)	118.18	0.000	53.75	0.64
14	TSA _2_	83.59 (1.89)	921.44 (20.88)	64.94 (1.47)	44.125	0.000	58.12	0.51
15	TSA _3_	8.82 (1.70)	124.96 (24.15)	5.17 (1.00)	5.17	0.000	68.98	0.81
16	TSA _4_	5.08 (2.11)	102.54 (42.65)	2.64 (1.10)	2.404	0.000	81.52	0.82
17	TSA _5_	2.11 (2.45)	38.69 (44.81)	1.24 (1.44)	0.863	0.000	91.72	0.72
18	TRV _1_	0.02 (2.80)	0.13 (16.20)	0.01 (1.76)	0.0078	0.000	53.42	0.66
19	TRV _2_	0.02 (1.84)	0.27 (21.33)	0.02 (1.39)	0.0125	0.000	58.67	0.55
20	TRV _3_	0.01 (1.71)	0.12 (24.34)	0.00 (0.99)	0.0048	0.505	69.12	0.81
21	TRV _4_	0.01 (2.15)	0.19 (42.54)	0.01 (1.11)	0.0045	0.000	81.78	0.82
22	TRV _5_	0.01 (2.41)	0.12 (45.07)	0.00 (1.44)	0.0027	0.006	92.77	0.72
23	TRT _1_	2,44,981.00 (4.35)	1,182,454.69 (21.00)	1,76,155.50 (3.13)	56,310	0.000	64.89	0.50
24	TRT _2_	117.44 (5.21)	1,524.26 (67.57)	98.54 (4.37)	22.56	0.000	59.49	0.31
25	TRT _3_	1.56 (1.55)	0.00 (0.00)^**NS**^	1.77 (1.76)	1.00	0.000	88.67	0.28
26	TRT _4_	0.52 (1.22)	0.55 (1.28)	0.52 (1.20)	0.43	0.000	79.1	0.05
27	TRT _5_	0.17 (1.08)	0.18 (1.16)	0.20 (1.30)	0.16	0.000	86.48	0.41

**Note:**

Where: PRL, primary root length; TSA, total root surface area; TRL, total root length; TRV, total root volume; TRF, total root forks; TRT, total root tips; RAD, root average diameter; TRL _1–5_, RSA _1–5_, TRV _1–5_, TRT _1–5_ indicate total root length, root surface area, root volume and root tips in diameter between (1) 0.0 to 0.5 mm, (2) 0.5 to 1.0 mm, (3) 1.0 to 1.5 mm, (4) 1.5 to 2 mm and (5) greater than 2.0 mm. df represents the degree of freedom.

### Genetic correlations

The Pearson correlation coefficients between all of the characteristics were evaluated under two P treatments, with significant correlations between sets of characters being listed (Table S3). Under SP treatment, there was a highly positive and significant association between TRV and TRF (r = 0.760), TSA and TRV (r = 0.681), and TRL and TSA (r = 0.608). TRF had a highly significant relationship with TRV (r = 0.76) and TSA (r = 0.513), while TRV and TRF (r = 0.816) had a favorable and highly significant association in DP treatment, followed by TSA and TRV (r = 0.665), and TSA and TRT (r = 0.590).

TRV had a clear positive significant correlation with most other assessed characteristics in both treatments. Except for RAD, the TRL and PRL in both treatments had a significant positive correlation with all other attributes. RAD had a clear negative interaction with TRT in both P regimes, as well as TRV and TRF in the DP scenario. In the SP treatment, the PRL and RAD, as well as the RAD and TRT, were significantly lower than in the DP treatment. The SP and DP treatments had a very different association between PRL and RAD, as well as RAD and TRT.

### Variability in fine roots and its distribution under two phosphorus treatments

Root diameter intervals were used to divide TSA, TRL, TRT, and TRV into five categories 0 to 0.5 mm, 0.5 to 1.0 mm, 1.0 to 1.5 mm, 1.5 to 2.0 mm, and >2.0 mm, and were assigned the names TRL_1–5_, RSA_1–5_, TRV_1–5_, and TRT_1–5_. These root diameter intervals can be used to compare and understand fine root variability across various diameter intervals in both P treatments. The proportions of roots for every diameter group were measured as a percentage of the total by each lentil line group in two phosphorus treatments (Table S4). For all genotypes except the ABL group, the portion of roots with diameters of 1.5 to 2.0 mm and >2.0 mm was higher in DP than in SP treatment (Fig. 1). TRL, RSA, TRV, and TRT traits showed a higher proportion in SP as compared to DP treatment for diameter group 0–0.5 mm (diameter class with % of genotypes in each traits).; for diameter group 0.5 to 1.0 mm, TRL, RSA, and TRV traits showed a higher proportion in SP as compared to DP treatment except in exotic germplasm (EG) genotypes; and for diameter group, 1.0 to 1.5 mm, TRL, RSA, and TRV traits showed a higher proportion in SP as Except in released varieties (RV) and exotic germplasm (EG), diameter groups 0.5 to 1.0 mm and 1.0 to 1.5 mm showed higher percentages in SP relative to DP treatment in TRT.

### Genetic variability among various groups of lentil genotypes

The tested root morphology traits between different groups showed clear-cut variation. The lentil genotypes were classified into four categories namely released variety (RV) advanced breeding lines (ABL), exotic germplasm (EG), and indigenous germplasm (IG) groups. Each root trait was graded using mean and standard deviation into low, medium, and high under SP and DP treatments based on their performance (Table S5). This characterization primarily depicts the genotype frequency distribution for the root traits under evaluation in both SP and DP scenarios. For all of the traits analyzed, a greater proportion of genotypes were assigned to the medium performance group. A crucial review of performance against different P treatments showed that the greater numbers of lentil lines were concentrated in the high category for traits like PRL, TRT, and RAD among the genotypes assessed at the DP treatment. Thirteen ABL group genotypes (26.53 percent) and seven EG group genotypes (16.66 percent) displayed higher for TSA under DP treatment. In both the treatments, the RV group had a smaller number of high-performance genotypes (≥${\rm{\bar{x}}}$ + SD) for all traits except TSA and RAD. But, the IG exhibited a lower percentage of high-performance genotypes than the ABL and EG groups for all traits. Meanwhile, ABL showed a smaller proportion of high-performance genotypes (≥${\rm{\bar{x}}}$ + SD) for all characteristics excluding TSA, TRV, and TRF than the EG groups. This indicates that under the two P treatments, the RV > IG > ABL > EG categories have a higher number of genotypes with phosphorus responsive root traits.

The Shannon-Weaver diversity index (H′) was used to look at the variation in assessed traits across genotypic classes (Table S5). The H′ values ranged with a mean of 0.76 in lentil genotypes for the root traits evaluated. H′ was highest for ABL and lowest for IG among many of the groups studied. Except for TRT and TRF, all other traits in tested lentil genotypes provided higher H′ values in SP treatment, while PRL, TSA, and RAD had higher H′ values in DP treatment. RAD, PRL, and TSA displayed a significantly higher level of variation in both the P conditions, whereas TRT and TRF were less variable among various genotypic classes. Except for PRL, TSA, and TRT, all root characteristics exhibited lower H′ values, suggesting lower diversity under the DP treatment than the SP treatment. In the RV and IG groups, PRL exhibited greater diversity than in the ABL and EG groups, TRL displayed greater diversity in the ABL and IG groups than in the RV and EG groups, and RAD, TRV, and TRF revealed greater diversity across all four groups under the SP condition. In the ABL, EG, and IG groups, TSA displayed greater diversity than in the RV group and TRT under DP treatment indicated greater diversity in all four categories.

### PCA analysis: most contributing traits

Principal component analysis (PCA) was carried out to explore the relationship between traits and lentil genotypes under SP and DP conditions. The Eigen values with the highest absolute value of each of the significant PCs (PC I, PC II, PC III, PC IV, and PC V) were selected (Table S6). The first five components in the PCA analysis with Eigen values > 1 contributed 81.26 percent (under SP) and 79.76 percent (under DP) making them significant PCs. Loading factors of all seven PC were presented in (Tables S7 and S8). Moreover, the first PC explained 45.89% (in SP) and 50.90% (in DP) of the data variation. These variables corresponded to the values highlighted in bold in case of three major PCs (Table S9). Remaining components with Eigen values < 1 contributed 18.71 percent (under SP) and 20.24 percent (under DP) percent variability respectively. TSA and TRV are the most important contributory features, explaining 45.89 percent and 50.90 percent of the overall variability in SP and DP treatment, respectively, according to the first principal variable. In the case of the second and third main components, high contributors were RAD and TRF, TRT and TRF respectively under SP treatment. PRL and TRF, RAD, and PRL were the most important contributors to the second and third major components under the DP treatment. The PCA biplots (PC 1 *vs* PC 2) under SP and DP showed the distribution of lentil genotypes as shown in Fig. S1 and traits in Fig. S2.

### Loading factor of first three components

**Under SP conditions:** The bar represents the magnitude and nature (positive or negative) for each variable “loaded” on the component. Based on the plots, it can be noted that variable TSA and TRV are highly loaded on PC I (Fig. S3). Thus, we can say that variable TSA and TRV are similar on the PC 1. The loading of RAD on PC 1 was negative. We can also see variable RAD dominated the PC 2. TRL and TSA variables are negatively loaded on PC II. In case of PC III, TRF and TRV dominated and PRL and TRT were found negative. The most variables on PC III (TRL, TSA, RAD, TRV and TRF) were positively loaded, but they are all less than 0.5 indicating their least significance.

**Under DP conditions:** Based on the loading graphs, it can be noted that variables TSA, TRV and TRF are loaded on PC I, but they are all less than 0.5. The loading of RAD on PCI and PC II was negative (Fig. S3). We can also see variable RAD dominated the PC III positively with value more than 0.5. TRL, TSA, RAD, TRV and TRF variables are negatively loaded on PC II with values less than 0.5 indicating their least significance. In case of PC III, TRL was found to be negative. The most variables on PC III (TRL, TSA, RAD, TRV and TRF) were positively loaded, but they are all less than 0.5.

### Phosphorus efficiency by CPEM value

To compare the effectiveness of morphological root traits between lentil genotypes under P deficiency, the comprehensive synthetic index (CPEM) value was calculated using subordination function value analysis (Table S10). All lentil genotypes were divided into five classes based on CPEM values. CPEM values greater than 1.3 represent 11 genotypes under high P efficiency in group I. P efficiency was observed in group II, which had 15 genotypes and CPEM values ranging from 1.0 to 1.3. The III group with 30 genotypes displaying CPEM values between 0.8 and 1.0 indicating moderate efficiency. Group IV had 45 genotypes of P inefficiency and CPEM values ranging from 0.5 to 0.8, whereas group V had nine genotypes with P inefficiency and CPEM values less than 0.5. In Table S10 and Fig. S4 the difference in the root architectural characteristics of contrasting genotypes are shown based on CPEM values.

Especially in comparison to the SP treatment, the PLL 18-9 genotype with the greatest CPEM value scored a larger score for all traits except TRL under the DP treatment. In contrast to SP treatment, IG 112131 with the lowest CPEM value was found to be the worst performer under DP treatment for traits TRL, TRV, TRT, TSA, and TRF. Figure S5 indicates the average values of the phosphorus efficiency coefficients for every characteristic in five classes of various P efficiency levels. For all root characteristics, Group I had the highest mean P efficiency coefficient values, whereas Groups II, III, and IV had moderate values, and Group V had the lowest value other than TRL. This implies that phosphorus-efficient lentil lines with higher CPEM values were also more phosphorus-efficient lentil lines.

## Discussion

### Variation in root traits under P deficit treatment

Lentil accessions varied in their response to the deficit P conditions. The response of various crops to P has been varied, soybean (Zhou et al., 2016), faba bean (Daoui et al., 2012), common bean (Miguel, 2004), lentil (Gahoonia et al., 2006), mungbean (Reddy et al., 2020a), wheat (Ortiz-Monasterio, Manske & Van Ginkel, 2001; Ozturk et al., 2005), maize (Zhu & Lynch, 2004), barley, oats, rye, and triticale (Sepehr et al., 2009), cotton (Iqbal et al., 2019; Chen et al., 2019), brassica (Hammond et al., 2009) and the groundnut (Kumar, Kusuma & Gowda, 2009). Numerous studies have commonly used the targeting with few P-use efficiency measures (Fageria, Baligar & Li, 2008) and some other studies have targeted specific root architectural characteristics (Lynch & Brown, 2008) and consideration has also been given to another vital feature of root hair formation (Gahoonia & Nielsen, 2004). All these studies have shown that there are major genotypic differences for different traits in different crop species. The current study found a significant difference in the root traits of 110 lentil genotypes. The current research not only highlights the importance of identifying phosphorus-efficient germplasm for lower phosphorus environments, but it also demonstrates extremely efficient and in-efficient lentil lines when given enough phosphorus. Traditionally, a greater RSR (root-to-shoot ratio) was regarded to be a P efficiency index attributable because of an improvement in root biomass and a deeper RSA capable of extracting further nutrients (Swinnen, 1994; Nielsen, Eshel & Lynch, 2001). The various components of the RSA were for the detection of genotypes under P deficit treatments. The effects of P deficiency on the root architectural features of 110 lentil genotypes were investigated, as well as the different root characteristics TRL, PRL, TRV, TSA, TRF, and TRT.

For these root traits, we found considerable heterogeneity, heritability ranging from low to high, significant associations, and a nearly normal distribution. A low CV (coefficient of variation) and a high heritability mean that the characteristics for this group of lentil lines are genetically stable. The results were consistent with previous studies by other researchers (Da Silva et al., 2016; Wang et al., 2017; Wang et al., 2019). In response to abiotic stresses, the root characteristics, particularly root diameter, increase (hypertrophy) (Franco et al., 2011). The root diameter has the most impact on the root’s surface area and volume, resulting in a higher dry weight (Wasaya et al., 2018). In our study RAD, the commonly used predictor for P studies, was highly heritable, meaning it is a more robust P efficiency attribute.

### Fine and very fine roots distribution

Although the root system’s genetic variability varies by crop, the bulk of root features are defined by the formation of very fine roots with a diameter of less than 0.5 mm and fine roots with a diameter of 0.5 to 2.0 mm, which are necessary for nutrient uptake and water absorption (Hendricks et al., 2006; Zobel & Waisel, 2010; Liu et al., 2010). The proportion of fine roots in the diameter groups 1.5 to 2.0 mm and >2.0 mm was greater in the DP treatment than the SP treatment in this experiment. The proportion of fine root distribution in different diameter classes of lentil genotypes is influenced by the presence of P, as shown in this analysis. Crops may respond to lower P levels by enhancing the growth of cortical aerenchyma root tissue, allowing the crop plants to have a larger root average diameter while reducing total root respiration at the expense of total root cost (Postma & Lynch, 2011; McCormack & Guo, 2014). Increased fine root development allows the maximum adsorption region to expand primarily as a stress tolerance adaptive response (Arif, Islam & Robin, 2019). The principal component analysis indicated that the root morphological traits TSA, RAD, TRV, PRL, and TRT accounted for the majority of the phenotypic variation in the lentil genotypes at the seedling stage.

### Genetic association and trait improvement

The TRL (total root length) is positively and significantly associated with TRV, TRF, and TSA in SP and DP treatments, but negatively with RAD. Based on the principal component analysis, TRL, TRV, TSA, and TRT were found to be appropriate parameters for P uptake efficiency testing at the seedling level. TRT and TRF were strongly and negatively associated with RAD during the DP treatment. This shows that RAD plays a key role in distinguishing P availability from the other root traits studied. Furthermore, these attributes had greater P efficiency coefficient values in P efficient lentil genotypes. This result is in line with the previous studies (Pandey et al., 2014; Swinnen, 1994; Nielsen, Eshel & Lynch, 2001; Wasaya et al., 2018). The P efficient mungbean genotype has a significantly greater root volume and root surface area under P stress (Pandey et al., 2014). The rate of absorption of nutrients has also been reported to be significantly associated with the root surface area (Imada, Yamanaka & Tamai, 2008; Bates & Lynch, 2000). During the plant’s early development stage, aggressively growing roots with a large surface area and root length allow successful macro and micronutrient absorption (Wang et al., 2016). Furthermore, root structural attributes, especially TRL and root number, were positively and significantly correlated with biomass production and grains output (Xie et al., 2017; Liu et al., 2018). The plant’s strong root system benefits not only in the growth of better crops, but also in the plant’s long-term survival in adverse conditions. Variability in root architecture helps maximise the efficiency of nutrient and water use in difficult situations. The ability to adapt to a wide range of genotypes of lentils, as well as the ability to tolerate stress, will be the most important factors in the breeding scheme’s success. To classify genotypes into high and low-performing groups, the standard deviation and mean performance of root properties were utilised as eligibility criteria (Li et al., 2015; Zar, 2010).

Besides, the H′ index (Shannon-Weaver diversity index) was estimated to quantify phenotypic diversity among these traits. PRL, RAD, and TSA (>0.8) revealed a high H′ value in both treatments among all characteristics, and RAD displayed the relatively highest H′ (>0.90) value within both P treatments. Greater root diameter diversity is attributed to an improvement in the fine root dispersion by root diameter class, regardless of root length, in nutritionally better conditions (Zobel, Kinraide & Baligar, 2007). A greater H′ value points to a balanced distribution of frequency and increased higher genetic variability, while a decrease in diversity is pointed out by unbalanced frequency distribution (extremely) and a low H′ value (Hutcheson, 1970; Yadav et al., 2018; Rathinavel, 2017). An analysis of root morphological characteristics among different genotypic groups revealed in this analysis that the ABL group demonstrated high root characteristic diversity than the EG, IG, and RV groups. The existence of high H values for TSA, TRL, TRV, and RAD in the ABL group with moderate to high heritability shows that these genotypes used in the study are a rich source of improvement in P efficiency under DP treatment. Broad-sense heritability analysis (H^2^), the principal component analysis, and the Shannon-Weaver diversity index help to increase the efficiency of target genotype selection for cowpea root architectural characteristics (Adu et al., 2019).

Besides, RAD and TRF were defined in this study to be unfavourable root characteristics influenced by P uptake. The same is observed in mungbean under P deficiency environments (Krishnapriya & Pandey, 2016). TRF is much less variable in drought stress at the seedling stage due to the lower H′ value in all maize lines evaluated (Pérez-Torres et al., 2008). It was recorded that, with the N-optimum state, all the variables enhanced during N-stress excluding root forks (Nagar et al., 2018). All root characteristics excluding RAD in maize displayed greater H′ values and greater diversity under circumstances of water stress (Pérez-Torres et al., 2008). Potassium deficiency leads to a decrease of RAD in the sensitive genotype in comparison to the sweet potato resistant genotype (Liu et al., 2017). To increase soil exploration and minimise metabolic costs, the term ‘root etiolation’ was proposed to characterise the reduction in root diameter of common bean genotypes in P stress (Morrow de la Riva, 2010). Such detrimental consequences due to unwanted characteristics lead to a decline in the acquisition of P by crops. TRL with a higher H′ value and a significantly positive relationship with TRV and TSA, as well as correlation and principal components analysis, suggests that such attributes are appropriate enough to reflect the differences and could be used as an eligibility yardstick for phosphorus performance evaluation at the seedling phase.

The most significant factor to genetic differences in both P treatments between these different root traits was identified to be root average diameter (RAD). There is a decrease in RAD in low P treatment, which is confirmed by previous studies. In white clover (*Trifolium repens*), the smaller the root diameter, the higher the soil volume per unit surface area (Woodfield & Caradus, 1990). P acquisition is effective in genotypes with thinner roots. The studies showed that in mutants of Arabidopsis and Spinach (*Spinacia oleracea*), a greater root surface area was obtained by decreasing the mean root diameter, resulting in thinner roots (Fitter et al., 2002). Small-diameter roots were thought to be an essential and cost-effective way to boost P acquisition in crop plants (Lynch, 2011, 2013).

Root dry weight (RDW) and TRL have been the most effective of adding to total phenotypic variability in maize, and they were essential for other root parameters (Pérez-Torres et al., 2008; Kumar et al., 2012). This study adds to our understanding of how to improve the efficiency of agronomic and nutrient-use features in lentil breeding. In contrast to the current era, nutrient-efficient crops play a critical role in boosting plant production in the new millennium, despite environmental challenges and the exorbitant prices of synthetic fertilizers (Fageria, Baligar & Li, 2008). Based on a detailed index of CPEM values, the lentil genotypes were graded from highly efficient to highly inefficient. Among these, the best five highly efficient genotypes were listed as PLL 18-9, PLS-18-01, PLL 18-25, PLS 18-23, and PLL 18-07 whereas the highly inefficient genotypes were IG 112131, P 560206, IG-334, L 11-231, and PLS 18-67.

Based on P efficiency coefficients, group 4 had the largest number of genotypes, followed by group 3 and group 2, while group 5 had the lowest genotype number. For the selection and identification of lentil lines with favorable root attributes in varying P environments, this form of grouping is necessary. These contrasting genotypes can also be targeted in a recombination breeding plan to develop P-efficient varieties (Gill et al., 2004; Aziz et al., 2011). This research identified 11 lentil genotypes with well-defined root characteristics and high efficiency that might be employed in the lentil breeding programme to improve abiotic stress resistance. In conclusion, the hydroponic testing preserved the lentil root system architecture (RSA) throughout the screening process. This hydroponic evaluation method is particularly effective in screening bigger sets of germplasm with minimal environmental impact (Rajkumar & Ibrahim, 2014).

Research studies successfully investigated root attributes in crops such as soybean (Ochigbo & Bello, 2014), mungbean (Leiser et al., 2014; Pandey et al., 2014; Reddy et al., 2020a, 2020b), maize (Gong et al., 2011), rice (Negi et al., 2016), and wheat (Liu et al., 2016) using a common hydroponic model that focuses on the oxygenated nutrient solution with varying levels of phosphorus.

## Conclusions

At the seedling stage, we found a wide range of reactions to P deficiency scenarios in lentil genotypes for root morphological parameters. The most efficient selection criteria for projecting nutrient utilisation performance during the seedling phase were identified to be the TRL, TRV, and TSA. By controlling access to water and nutrients, the root morphology reactions to P deficit in hydroponics can be explored without causing root damage. Furthermore, genotypes of lentils needs to be investigated at the adult stage in both P deficiency and P adequate conditions. In addition, the complicated interaction of root characteristics and capabilities between adult and seedling stage roots needs additional investigation.

## Supplemental Information

10.7717/peerj.12766/supp-1 Supplemental Information 1 List of diverse lentil germplasm used in the study.

10.7717/peerj.12766/supp-2 Supplemental Information 2 Various root traits, their units and how measured in the study.

10.7717/peerj.12766/supp-3 Supplemental Information 3 Supplementary Table 3. Eigen values and % of variance explained by principal components in the study.

10.7717/peerj.12766/supp-4 Supplemental Information 4 Supplementary Table 4. Loading factors of seven principal components (Eigen vectors) under sufficient P conditions. Where A: TRL (total root length); B: PRL (primary root length); C: RAD (root average diameter); D: TSA (total root surface area); E: TRF(total root forks); F: TRT( total root tips); G: TRV(total root volume).

10.7717/peerj.12766/supp-5 Supplemental Information 5 Supplementary Table 5. Loading factors of seven principal components (Eigen vectors) under deficit P conditions. Where A: TRL (total root length); B: PRL (primary root length); C: RAD (root average diameter); D: TSA (total root surface area); E: TRF (total root forks); F: TRT (total root tips); G: TRV (total root volume).

10.7717/peerj.12766/supp-6 Supplemental Information 6 Supplementary Table 6. Under 2 distinct phosphorus conditions, the H′ index (Shannon-Weaver diversity index) in terms of high (H), medium (M), and low (L) diversity groups in different classes of lentil genotypes. **(**DP, deficit phosphorus; SP, sufficient phosphorus; Total 110 lentil genotypes: RV, released varieties: EG, exotic germplasm lines: IG, indigenous germplasm lines; ABL, advanced breeding lines: RSA, root surface area; TRL, Total root length; TRV, total root volume; TRT, total root tips;)

10.7717/peerj.12766/supp-7 Supplemental Information 7 Supplementary Table 7. The highly contributing traits, Eigen values, % variance, and cumulative % variance in two phosphorus conditions indicated by principal component analysis. DP, deficit phosphorus; SP, sufficient phosphorus; PCI, principal component one; PCII, principal component two; PCIII, principal component three; TRL, total root length; PRL, primary root length; TSA, total root surface area; TRV, total root volume; TRF, total root forks; RAD, root average diameter; TRT, total root tips.

10.7717/peerj.12766/supp-8 Supplemental Information 8 Supplementary Table 8. Top eight and bottom eight lentil genotypes based on the CPEM values under sufficient and deficit phosphorus constraints. Contrasting genotypes identified using Comprehensive phosphorus efficiency measurement value. TSA, total root surface area; PRL, primary root length; RAD, root average diameter; TRL, total root length; TRF, total root forks: TRT, total root tips; TRV, total root volume. DP, deficit phosphorus: SP, sufficient phosphorus.

10.7717/peerj.12766/supp-9 Supplemental Information 9 Supplementary Table 9. The highly contributing traits, Eigen values, % variance, and cumulative % variance in 2 phosphorus conditions indicated by principal component analysis. DP, deficit phosphorus; SP, sufficient phosphorus; PCI, principal component one; PCII, principal component two; PCIII, principal component three; TRL, total root length; PRL, primary root length; TSA, total root surface area; TRV, total root volume; TRF, total root forks; RAD, root average diameter; TRT, total root tips.

10.7717/peerj.12766/supp-10 Supplemental Information 10 Supplementary Table 10. Top eight and bottom eight lentil genotypes based on the CPEM values under sufficient and deficit phosphorus constraints. Contrasting genotypes identified using Comprehensive phosphorus efficiency measurement value. TSA, total root surface area; PRL, primary root length; RAD, root average diameter; TRL, total root length; TRF, total root forks: TRT, total root tips; TRV, total root volume. DP, deficit phosphorus: SP, sufficient phosphorus.

10.7717/peerj.12766/supp-11 Supplemental Information 11 Supplementary Figure 1. The PCA biplots and distribution of lentil genotypes under SP and DP conditions in lentil respectively. (Refer the supplementary table 1 for genotype numbers.)

10.7717/peerj.12766/supp-12 Supplemental Information 12 Supplementary Figure 2. The biplots of first three components (PC I, II, and III) under SPand DP conditions in lentil. (Where, A:TRL-total root length; B:PRL-primary root length; C:TSA -total root surface area -; D:TRV, total root volume; E: RAD- root average diameter;F: TRF-total root forks; G: TRT-total root tips.)

10.7717/peerj.12766/supp-13 Supplemental Information 13 Supplementary Figure 3. The loadings factor (trait wise) of first three components (PC I, II, and III) under SP and DP conditions in lentil. (Where, TRL-total root length; PRL-primary root length; TSA -total root surface area -; total root volume-TRV; RAD- root average diameter; TRF-total root forks; TRT-total root tips.)

10.7717/peerj.12766/supp-14 Supplemental Information 14 Supplementary Figure 4. Variations in root system architectural traits between lentil genotypes grown under contrasting phosphorus levels. Names of the genotypes

10.7717/peerj.12766/supp-15 Supplemental Information 15 Supplementary Figure 5. The trait-wise classifications of lentil genotypes into highly inefficient to highly efficient based on phosphorus efficiency coefficient values. **(**Where, TRL-total root length; PRL-primary root length; TSA -total root surface area -; total root volume-TRV; RAD- root average diameter; TRF-total root forks; TRT-total root tips. Lentil genotypes classified into highly efficient to highly inefficient are represented by groups in different colors. For classes 1, 2, 3, 4, and 5, N = 11, 15, 30, 45, and 9 respectively for each class.)

10.7717/peerj.12766/supp-16 Supplemental Information 16 Raw data tables. Raw data tables of main root traits and root diameter classes

## Additional Information and Declarations

### Data Availability

The following information was supplied regarding data availability: The raw measurements are available in the Tables, Supplementary Tables and Figures.
