# Supplementary material for: Genotypic variation in root architectural traits under contrasting phosphorus levels in Mediterranean and Indian origin lentil genotypes
Source: PeerJ. 2022 Mar 10;10:e12766. doi: 10.7717/peerj.12766 (PMC8918163; doi:10.7717/peerj.12766)
Supplement: Supplemental Information 1 [file peerj-10-12766-s001.doc]

Supplementary Table 1. List of diverse lentil germplasm used in the study

| **S.NO.** | **Name** | **CLASS** | **Description** |
| --- | --- | --- | --- |
| 1 | HM-1 | RV | Released Variety |
| 2 | L 4649 | ABL | Advanced Breeding Line |
| 3 | IG 69568 | MOLG | Mediterranean Origin Lentil Germplasm |
| 4 | IC 321808 | IOLG | Indian Origin Lentil Germplasm |
| 5 | IC 560135 | IOLG | Indian Origin Lentil Germplasm |
| 6 | IG 136607 | MOLG | Mediterranean Origin Lentil Germplasm |
| 7 | IG-Y- 50 | ABL | Advanced Breeding Line |
| 8 | IG 134349 | MOLG | Mediterranean Origin Lentil Germplasm |
| 9 | LL 699 | ABL | Advanced Breeding Line |
| 10 | IC 268238 | IOLG | Indian Origin Lentil Germplasm |
| 11 | ILWL-95 | MOLG | Mediterranean Origin Lentil Germplasm |
| 12 | ILWL-15 | MOLG | Mediterranean Origin Lentil Germplasm |
| 13 | IG 73798 | MOLG | Mediterranean Origin Lentil Germplasm |
| 14 | EC 78472 | MOLG | Mediterranean Origin Lentil Germplasm |
| 15 | L5126 | ABL | Advanced Breeding Line |
| 16 | Fasciated mutant | IOLG | Indian Origin Lentil Germplasm |
| 17 | L 4618 | ABL | Advanced Breeding Line |
| 18 | IC 560181 | IOLG | Indian Origin Lentil Germplasm |
| 19 | IG 129185 | MOLG | Mediterranean Origin Lentil Germplasm |
| 20 | IG 49 | MOLG | Mediterranean Origin Lentil Germplasm |
| 21 | IG 112078 | MOLG | Mediterranean Origin Lentil Germplasm |
| 22 | IG 73920 | MOLG | Mediterranean Origin Lentil Germplasm |
| 23 | P 3233 | MOLG | Mediterranean Origin Lentil Germplasm |
| 24 | IG 560183 | MOLG | Mediterranean Origin Lentil Germplasm |
| 25 | IPL 406 | RV | Released Variety |
| 26 | IG 560206 | MOLG | Mediterranean Origin Lentil Germplasm |
| 27 | L 4076 | RV | Released Variety |
| 28 | IG 134656 | MOLG | Mediterranean Origin Lentil Germplasm |
| 29 | IG 560185 | MOLG | Mediterranean Origin Lentil Germplasm |
| 30 | L 4698 | ABL | Advanced Breeding Line |
| 31 | PL 97 | RV | Released Variety |
| 32 | P 2208 | MOLG | Mediterranean Origin Lentil Germplasm |
| 33 | ILL 10821 | MOLG | Mediterranean Origin Lentil Germplasm |
| 34 | IG 936 | MOLG | Mediterranean Origin Lentil Germplasm |
| 35 | IPL 321 | RV | Released Variety |
| 36 | P 2116 | MOLG | Mediterranean Origin Lentil Germplasm |
| 37 | IG 334 | MOLG | Mediterranean Origin Lentil Germplasm |
| 38 | IG 560157 | MOLG | Mediterranean Origin Lentil Germplasm |
| 39 | IG 568229 | MOLG | Mediterranean Origin Lentil Germplasm |
| 40 | IG 130033 | MOLG | Mediterranean Origin Lentil Germplasm |
| 41 | IG 112131 | MOLG | Mediterranean Origin Lentil Germplasm |
| 42 | LC 300-19 | IOLG | Indian Origin Lentil Germplasm |
| 43 | Global Mutant | ABL | Advanced Breeding Line |
| 44 | ILL10832 | MOLG | Mediterranean Origin Lentil Germplasm |
| 45 | EC 78518 | MOLG | Mediterranean Origin Lentil Germplasm |
| 46 | IC 560162 | IOLG | Indian Origin Lentil Germplasm |
| 47 | P 2130 | MOLG | Mediterranean Origin Lentil Germplasm |
| 48 | L 4603 | ABL | Advanced Breeding Line |
| 49 | P 8116 | MOLG | Mediterranean Origin Lentil Germplasm |
| 50 | L 4705 | ABL | Advanced Breeding Line |
| 51 | IG 56968 | MOLG | Mediterranean Origin Lentil Germplasm |
| 52 | IG 129302 | MOLG | Mediterranean Origin Lentil Germplasm |
| 53 | P 3226 | MOLG | Mediterranean Origin Lentil Germplasm |
| 54 | P 560183 | MOLG | Mediterranean Origin Lentil Germplasm |
| 55 | IPL 406-1 | RV | Released Variety |
| 56 | P 560206 | MOLG | Mediterranean Origin Lentil Germplasm |
| 57 | L 4076-1 | RV | Released Variety |
| 58 | IG 134656 | MOLG | Mediterranean Origin Lentil Germplasm |
| 59 | P 560185 | MOLG | Mediterranean Origin Lentil Germplasm |
| 60 | PL 97-1 | RV | Released Variety |
| 61 | ILWL 147 | MOLG | Mediterranean Origin Lentil Germplasm |
| 62 | IG 129313 | MOLG | Mediterranean Origin Lentil Germplasm |
| 63 | LC 282-1077 | IOLG | Indian Origin Lentil Germplasm |
| 64 | IC 560297 | IOLG | Indian Origin Lentil Germplasm |
| 65 | IC 27986 | IOLG | Indian Origin Lentil Germplasm |
| 66 | IC 346092 | IOLG | Indian Origin Lentil Germplasm |
| 67 | P 43120 | MOLG | Mediterranean Origin Lentil Germplasm |
| 68 | P 43103 | MOLG | Mediterranean Origin Lentil Germplasm |
| 69 | P 16214 | MOLG | Mediterranean Origin Lentil Germplasm |
| 70 | P 16213 | MOLG | Mediterranean Origin Lentil Germplasm |
| 71 | L 11-280 | ABL | Advanced Breeding Line |
| 72 | L 11-279 | ABL | Advanced Breeding Line |
| 73 | L 11-248 | ABL | Advanced Breeding Line |
| 74 | L 11-244 | ABL | Advanced Breeding Line |
| 75 | L 11-243 | ABL | Advanced Breeding Line |
| 76 | L 11-234 | ABL | Advanced Breeding Line |
| 77 | L 11-231 | ABL | Advanced Breeding Line |
| 78 | PLL-18-1 | ABL | Advanced Breeding Line |
| 79 | PLL 18-2 | ABL | Advanced Breeding Line |
| 80 | PLL 18-5 | ABL | Advanced Breeding Line |
| 81 | PLL 18-7 | ABL | Advanced Breeding Line |
| 82 | PLL 18-9 | ABL | Advanced Breeding Line |
| 83 | PLL 18-11 | ABL | Advanced Breeding Line |
| 84 | PLL 18-12 | ABL | Advanced Breeding Line |
| 85 | PLL 18-14 | ABL | Advanced Breeding Line |
| 86 | PLS-18-21 | ABL | Advanced Breeding Line |
| 87 | PLS -18-23 | ABL | Advanced Breeding Line |
| 88 | PLS 18-25 | ABL | Advanced Breeding Line |
| 89 | PLS 18-32 | ABL | Advanced Breeding Line |
| 90 | PLS 18-33 | ABL | Advanced Breeding Line |
| 91 | PLS 18-36 | ABL | Advanced Breeding Line |
| 92 | PLS 18-44 | ABL | Advanced Breeding Line |
| 93 | PLS 18-01 | ABL | Advanced Breeding Line |
| 94 | PLS 18-48 | ABL | Advanced Breeding Line |
| 95 | PLS 18-50 | ABL | Advanced Breeding Line |
| 96 | PLS 18-52 | ABL | Advanced Breeding Line |
| 97 | PLS 18-53 | ABL | Advanced Breeding Line |
| 98 | PLS 18-57 | ABL | Advanced Breeding Line |
| 99 | PLS 18-58 | ABL | Advanced Breeding Line |
| 100 | PLS 18-60 | ABL | Advanced Breeding Line |
| 101 | PLS 18-62 | ABL | Advanced Breeding Line |
| 102 | PLS 18-64 | ABL | Advanced Breeding Line |
| 103 | PLS 18-65 | ABL | Advanced Breeding Line |
| 104 | PLS 18-66 | ABL | Advanced Breeding Line |
| 105 | PLS 18-67 | ABL | Advanced Breeding Line |
| 106 | PLL 18-22 | ABL | Advanced Breeding Line |
| 107 | PLL 18-23 | ABL | Advanced Breeding Line |
| 108 | PLL 18-24 | ABL | Advanced Breeding Line |
| 109 | PLL 18-25 | ABL | Advanced Breeding Line |
| 110 | PLL 18-26 | ABL | Advanced Breeding Line |
