# Supplementary material for: Genotypic variation in root architectural traits under contrasting phosphorus levels in Mediterranean and Indian origin lentil genotypes
Source: PeerJ. 2022 Mar 10;10:e12766. doi: 10.7717/peerj.12766 (PMC8918163; doi:10.7717/peerj.12766)
Supplement: Supplemental Information 2 [file peerj-10-12766-s002.doc]

Supplementary Table 2. Various root traits, their units and how measured in the study

| **S.No.** | **Traits** | **Trait Descriptions** | **Units** | **Measured by** |
| --- | --- | --- | --- | --- |
| 1 | PRL | Primary Root Length (PRL) | cm | Manually |
| 2 | TRL | Total Root Length (TRL) | cm | WinRhizo^TM^ |
| 3 | TSA | Total Surface Area (TSA) | cm^2^ | WinRhizo^TM^ |
| 4 | RAD | Root Average Diameter (RAD), | mm | WinRhizo^TM^ |
| 5 | TRV | Total Root Volume (TRV) | cm^3^ | WinRhizo^TM^ |
| 6 | TRT | Total Root Tips (TRT) | Numbers | WinRhizo^TM^ |
| 7 | TRF | Total Root Forks (TRF) | Numbers | WinRhizo^TM^ |
| 8 | TRL _1_ | Total Root Length 1(0 to 0.5 mm) | mm | WinRhizo^TM^ |
| 9 | TRL _2_ | Total Root Length 2 (0.5 to 1.0 mm) | mm | WinRhizo^TM^ |
| 10 | TRL _3_ | Total Root Length 3(1.0 to1.5 mm) | mm | WinRhizo^TM^ |
| 11 | TRL _4_ | Total Root Length 4(1.5 to 2.0 mm) | mm | WinRhizo^TM^ |
| 12 | TRL _5_ | Total Root Length 5 (>2.0 mm) | mm | WinRhizo^TM^ |
| 13 | TSA _1_ | Total Surface Area 1(0 to 0.5 mm) | mm | WinRhizo^TM^ |
| 14 | TSA _2_ | Total Surface Area 2(0.5 to 1.0 mm) | mm | WinRhizo^TM^ |
| 15 | TSA _3_ | Total Surface Area 3(1.0 to1.5 mm) | mm | WinRhizo^TM^ |
| 16 | TSA _4_ | Total Surface Area 4(1.5 to 2.0 mm) | mm | WinRhizo^TM^ |
| 17 | TSA _5_ | Total Surface Area 5(>2.0 mm) | mm | WinRhizo^TM^ |
| 18 | TRV _1_ | Total Root Volume 1(0 to 0.5 mm) | mm | WinRhizo^TM^ |
| 19 | TRV _2_ | Total Root Volume 2(0.5 to 1.0 mm) | mm | WinRhizo^TM^ |
| 20 | TRV _3_ | Total Root Volume 3(1.0 to1.5 mm) | mm | WinRhizo^TM^ |
| 21 | TRV _4_ | Total Root Volume 4(1.5 to 2.0 mm) | mm | WinRhizo^TM^ |
| 22 | TRV _5_ | Total Root Volume 5(>2.0 mm) | mm | WinRhizo^TM^ |
| 23 | TRT _1_ | Total Root Tips 1(0 to 0.5 mm) | mm | WinRhizo^TM^ |
| 24 | TRT _2_ | Total Root Tips 2(0.5 to 1.0 mm) | mm | WinRhizo^TM^ |
| 25 | TRT _3_ | Total Root Tips 3(1.0 to1.5 mm) | mm | WinRhizo^TM^ |
| 26 | TRT _4_ | Total Root Tips 4(1.5 to 2.0 mm) | mm | WinRhizo^TM^ |
| 27 | TRT _5_ | Total Root Tips 5(>2.0 mm) | mm | WinRhizo^TM^ |
