# Supplementary material for: Genotypic variation in root architectural traits under contrasting phosphorus levels in Mediterranean and Indian origin lentil genotypes
Source: PeerJ. 2022 Mar 10;10:e12766. doi: 10.7717/peerj.12766 (PMC8918163; doi:10.7717/peerj.12766)
Supplement: Supplemental Information 3 [file peerj-10-12766-s003.docx]

**Supplementary Table 3. Correlations coefficients (Pearson) between evaluated characteristics in regimes of sufficient and deficient phosphorus (sample size = 110).**

| **SP** | **PRL** | **TRL** | **TSA** | **RAD** | **TRV** | **TRT** | **TRF** |
| --- | --- | --- | --- | --- | --- | --- | --- |
| **PRL** | 1 |  |  |  |  |  |  |
| **TRL** | 0.132 | 1 |  |  |  |  |  |
| **TSA** | 0.391*** | 0.608*** | 1 |  |  |  |  |
| **RAD** | -0.025 | -0.576*** | -0.112 | 1 |  |  |  |
| **TRV** | 0.384*** | 0.357*** | 0.681*** | 0.119 | 1 |  |  |
| **TRT** | 0.582*** | 0.177 | 0.470*** | -0.077 | 0.442*** | 1 |  |
| **TRF** | 0.307*** | 0.238** | 0.513*** | 0.131 | 0.760*** | 0.365*** | 1 |
| **DP** | **PRL** | **TRL** | **TSA** | **RAD** | **TRV** | **TRT** | **TRF** |
| **PRL** | 1 |  |  |  |  |  |  |
| **TRL** | 0.108 | 1 |  |  |  |  |  |
| **TSA** | 0.294** | 0.581*** | 1 |  |  |  |  |
| **RAD** | -0.163 | -0.445*** | -0.215** | 1 |  |  |  |
| **TRV** | 0.173 | 0.550*** | 0.665*** | -0.164 | 1 |  |  |
| **TRT** | 0.379*** | 0.415*** | 0.590*** | -0.343*** | 0.502*** | 1 |  |
| **TRF** | 0.101 | 0.549*** | 0.561*** | -0.288** | 0.816*** | 0.493*** | 1 |

(Where ** represents p*<*0.01 and *** represents p*<*0.001 at significance)

Where SP: sufficient phosphorus, DP: deficit phosphorus, TRL: total root length, TRV: total root volume, PRL: primary root length, TRT: total root tips, TSA: total root surface area, TRF: total root forks, RAD: root average diameter.
