# Supplementary material for: Genotypic variation in root architectural traits under contrasting phosphorus levels in Mediterranean and Indian origin lentil genotypes
Source: PeerJ. 2022 Mar 10;10:e12766. doi: 10.7717/peerj.12766 (PMC8918163; doi:10.7717/peerj.12766)
Supplement: Supplemental Information 4 — Where A: TRL (total root length); B: PRL (primary root length); C: RAD (root average diameter); D: TSA (total root surface area); E: TRF(total root forks); F: TRT( total root tips); G: TRV(total root volume). [file peerj-10-12766-s004.docx]

**Supplementary Table 4. The distribution in five root diameter classes (expressed in %) under various groups in sufficient and deficit phosphorus treatments.**

| **Traits** | **Treatment** | **Root Diameter classes in millimeter** | | | | | | | | | | | | | | | | | | | |
| --- | --- | --- | --- | --- | --- | --- | --- | --- | --- | --- | --- | --- | --- | --- | --- | --- | --- | --- | --- | --- | --- |
|  |  | **0 to 0.5** | | | | **0.5 to 1.0** | | | | **1.0 to 1.5** | | |  | **1.5 to 2.0** | | | | **˃2 (mm)** | | | |
|  |  | **RV** | **ABL** | **EG** | **IG** | **RV** | **ABL** | **EG** | **IG** | **RV** | **ABL** | **EG** | **IG** | **RV** | **ABL** | **EG** | **IG** | **RV** | **ABL** | **EG** | **IG** |
| **TRL (%)** | SP | 7.97 | 42.00 | 38.36 | 11.67 | 8.56 | 42.16 | 38.26 | 11.02 | 8.13 | 42.78 | 37.88 | 11.21 | 7.52 | 43.21 | 38.71 | 10.57 | 9.38 | 44.95 | 35.82 | 9.85 |
|  | DP | 7.17 | 43.98 | 37.67 | 11.18 | 7.90 | 39.53 | 41.68 | 10.88 | 8.93 | 37.20 | 41.86 | 12.01 | 9.80 | 33.25 | 44.33 | 12.62 | 9.94 | 32.65 | 44.02 | 13.39 |
| **RSA (%)** | SP | 7.83 | 41.97 | 38.48 | 11.72 | 8.54 | 42.23 | 38.20 | 11.04 | 8.14 | 42.80 | 37.84 | 11.22 | 7.52 | 43.28 | 38.68 | 10.52 | 9.42 | 45.00 | 35.82 | 9.76 |
|  | DP | 7.08 | 44.11 | 37.57 | 11.24 | 7.94 | 39.74 | 41.49 | 10.83 | 8.98 | 37.06 | 41.90 | 12.06 | 9.80 | 33.19 | 44.41 | 12.60 | 9.97 | 32.73 | 43.89 | 13.41 |
| **TRV (%)** | SP | 7.67 | 41.96 | 38.58 | 11.79 | 8.51 | 42.29 | 38.14 | 11.05 | 8.15 | 42.82 | 37.79 | 11.24 | 7.52 | 43.36 | 38.66 | 10.47 | 9.46 | 45.05 | 35.82 | 9.67 |
|  | DP | 7.05 | 43.89 | 37.71 | 11.35 | 7.97 | 39.95 | 41.29 | 10.78 | 9.02 | 36.92 | 41.93 | 12.12 | 9.81 | 33.12 | 44.49 | 12.58 | 10.00 | 32.82 | 43.76 | 13.42 |
| **TRT (%)** | SP | 6.33 | 45.03 | 35.23 | 13.41 | 6.51 | 46.52 | 32.11 | 14.86 | 7.99 | 42.81 | 30.99 | 18.21 | 9.09 | 46.10 | 34.42 | 10.39 | 14.75 | 39.34 | 27.87 | 18.03 |
|  | DP | 6.09 | 47.54 | 33.99 | 12.38 | 7.08 | 44.96 | 35.86 | 12.10 | 9.24 | 42.04 | 38.85 | 9.87 | 8.15 | 40.74 | 42.96 | 8.15 | 6.00 | 38.00 | 50.00 | 6.00 |

DP, deficit phosphorus; SP, sufficient phosphorus; Total 110 lentil genotypes: RV, released varieties: EG, exotic germplasm lines: IG, indigenous germplasm lines; ABL, advanced breeding lines: total root tips (TRT); total root length (TRL); total root volume (TRV); root surface area (RSA).
