# Supplementary material for: Genotypic variation in root architectural traits under contrasting phosphorus levels in Mediterranean and Indian origin lentil genotypes
Source: PeerJ. 2022 Mar 10;10:e12766. doi: 10.7717/peerj.12766 (PMC8918163; doi:10.7717/peerj.12766)
Supplement: Supplemental Information 5 — Where A: TRL (total root length); B: PRL (primary root length); C: RAD (root average diameter); D: TSA (total root surface area); E: TRF (total root forks); F: TRT (total root tips); G: TRV (total root volume). [file peerj-10-12766-s005.docx]

**Supplementary Table 5. Under 2 distinct phosphorus conditions, the H' index (Shannon-Weaver diversity index) in terms of high (H), medium (M), and low (L) diversity groups in different classes of lentil genotypes.**

| **Traits** | **Treatment** | **All 110 Lentil genotypes** | | | | **RV** | | | | **ABL** | | | | **EG** | | | | **IG** | | | |
| --- | --- | --- | --- | --- | --- | --- | --- | --- | --- | --- | --- | --- | --- | --- | --- | --- | --- | --- | --- | --- | --- |
|  |  | **H** | **M** | **L** | **H'** | **H** | **M** | **L** | **H'** | **H** | **M** | **L** | **H'** | **H** | **M** | **L** | **H'** | **H** | **M** | **L** | **H'** |
| **PRL** | SP | 15 | 75 | 20 | 0.84 | 1 | 6 | 1 | 0.74 | 4 | 34 | 11 | 0.79 | 8 | 26 | 8 | 0.93 | 2 | 9 | 0 | 0.47 |
|  | DP | 19 | 74 | 17 | 0.86 | 0 | 6 | 2 | 0.22 | 9 | 32 | 8 | 0.89 | 9 | 26 | 7 | 0.93 | 1 | 10 | 0 | 0.30 |
| **TRL** | SP | 25 | 66 | 19 | 0.95 | 1 | 7 | 0 | 0.38 | 20 | 18 | 11 | 1.07 | 25 | 66 | 19 | 0.04 | 2 | 9 | 0 | 0.47 |
|  | DP | 15 | 83 | 12 | 0.73 | 0 | 7 | 1 | 0.38 | 14 | 28 | 7 | 0.96 | 1 | 37 | 4 | 0.42 | 0 | 11 | 0 | 0.00 |
| **TSA** | SP | 16 | 78 | 16 | 0.80 | 2 | 3 | 3 | 1.08 | 10 | 32 | 7 | 0.88 | 3 | 33 | 6 | 0.66 | 1 | 10 | 0 | 0.30 |
|  | DP | 15 | 75 | 20 | 0.84 | 1 | 7 | 0 | 0.38 | 8 | 28 | 13 | 0.97 | 3 | 32 | 7 | 0.69 | 3 | 8 | 0 | 0.59 |
| **RAD** | SP | 18 | 66 | 26 | 0.94 | 3 | 4 | 1 | 0.97 | 5 | 25 | 19 | 0.94 | 10 | 29 | 3 | 0.79 | 0 | 8 | 3 | 0.59 |
|  | DP | 21 | 70 | 19 | 0.91 | 3 | 5 | 0 | 0.66 | 5 | 27 | 17 | 0.93 | 10 | 30 | 2 | 0.73 | 3 | 8 | 0 | 0.59 |
| **TRV** | SP | 22 | 69 | 19 | 0.92 | 3 | 2 | 3 | 1.08 | 10 | 28 | 11 | 0.98 | 5 | 32 | 5 | 0.71 | 4 | 7 | 0 | 0.66 |
|  | DP | 13 | 85 | 12 | 0.69 | 0 | 8 | 0 | 0.00 | 10 | 34 | 5 | 0.81 | 1 | 34 | 7 | 0.56 | 2 | 9 | 0 | 0.47 |
| **TRT** | SP | 13 | 95 | 2 | 0.45 | 1 | 7 | 0 | 0.38 | 3 | 45 | 1 | 0.33 | 6 | 35 | 1 | 0.52 | 3 | 8 | 0 | 0.59 |
|  | DP | 18 | 84 | 8 | 0.69 | 1 | 5 | 2 | 0.90 | 7 | 37 | 5 | 0.72 | 6 | 35 | 1 | 0.52 | 4 | 7 | 0 | 0.66 |
| **TRF** | SP | 16 | 83 | 11 | 0.72 | 2 | 4 | 2 | 1.04 | 6 | 38 | 5 | 0.69 | 5 | 34 | 3 | 0.61 | 3 | 7 | 1 | 0.86 |
|  | DP | 11 | 99 | 0 | 0.33 | 0 | 8 | 0 | 0.00 | 8 | 41 | 0 | 0.45 | 1 | 41 | 0 | 0.11 | 2 | 9 | 0 | 0.47 |

**(**DP, deficit phosphorus; SP, sufficient phosphorus; Total 110 lentil genotypes: RV, released varieties: EG, exotic germplasm lines: IG, indigenous germplasm lines; ABL, advanced breeding lines: RSA, root surface area; TRL, Total root length; TRV, total root volume; TRT, total root tips;)
