# Supplementary material for: Genotypic variation in root architectural traits under contrasting phosphorus levels in Mediterranean and Indian origin lentil genotypes
Source: PeerJ. 2022 Mar 10;10:e12766. doi: 10.7717/peerj.12766 (PMC8918163; doi:10.7717/peerj.12766)
Supplement: Supplemental Information 6 — (DP, deficit phosphorus; SP, sufficient phosphorus; Total 110 lentil genotypes: RV, released varieties: EG, exotic germplasm lines: IG, indigenous germplasm lines; ABL, advanced breeding lines: RSA, root surface area; TRL, Total root length; TRV, total root volume; TRT, total root tips;) [file peerj-10-12766-s006.docx]

**Supplementary Table 6. Eigen values and % of variance explained by principal components in the study**

|  | **P+ condition(SP)** | |  | **P- condition(DP)** | |  |
| --- | --- | --- | --- | --- | --- | --- |
| **PC** | **Eigen value** | **% variance** | **Cumulative variance** | **Eigen value** | **% variance** | **Cumulative variance** |
| **1** | **176181** | 45.89 | 45.89 | **345987** | 50.90 | 50.90 |
| **2** | **59965.5** | 21.99 | 67.88 | **28591.1** | 15.29 | 66.19 |
| **3** | **36449.4** | 13.32 | 81.20 | **17386.7** | 13.52 | 79.71 |
| **4** | **216.571** | 0.07938 | 81.279 | **168.501** | 0.042968 | 79.75 |
| **5** | **14.3806** | 0.005271 | **81.284651** | **24.9485** | 0.006362 | **79.759** |
| **6** | 0.015109 | 0.0000055379 | 81.284656 | 0.026213 | 0.0000066842 | 79.759007 |
| **7** | 0.00215207 | 0.00000078881 | 81.284657 | 0.002729 | 0.00000069579 | 79.759007 |
