# Supplementary material for: Genotypic variation in root architectural traits under contrasting phosphorus levels in Mediterranean and Indian origin lentil genotypes
Source: PeerJ. 2022 Mar 10;10:e12766. doi: 10.7717/peerj.12766 (PMC8918163; doi:10.7717/peerj.12766)
Supplement: Supplemental Information 7 — DP, deficit phosphorus; SP, sufficient phosphorus; PCI, principal component one; PCII, principal component two; PCIII, principal component three; TRL, total root length; PRL, primary root length; TSA, total root surface area; TRV, total root volume; TRF, total root forks; RAD, root average diameter; TRT, total root tips. [file peerj-10-12766-s007.docx]

**Supplementary Table 6. Loading factors of 7 principal components (Eigen vectors) under sufficient P conditions.**

|  | **PC 1** | **PC 2** | **PC 3** | **PC 4** | **PC 5** | **PC 6** | **PC 7** |
| --- | --- | --- | --- | --- | --- | --- | --- |
| **A** | 0.350 | 0.150 | -0.620 | 0.040 | 0.999 | -0.002 | 0.001 |
| **B** | 0.320 | -0.580 | 0.210 | -0.056 | 0.002 | 0.000 | 0.000 |
| **C** | **0.480** | -0.110 | 0.160 | 0.997 | -0.040 | -0.004 | -0.001 |
| **D** | -0.080 | **0.720** | 0.190 | 0.001 | 0.000 | 0.099 | 0.995 |
| **E** | **0.470** | 0.200 | **0.300** | 0.004 | 0.002 | 0.995 | -0.099 |
| **F** | 0.380 | 0.110 | -0.540 | -0.023 | -0.008 | 0.000 | 0.000 |
| **G** | **0.410** | 0.250 | **0.360** | -0.017 | -0.001 | 0.000 | 0.000 |

**Where A: TRL (total root length); B: PRL (primary root length); C: RAD (root average diameter); D: TSA (total root surface area); E: TRF(total root forks); F: TRT( total root tips); G: TRV(total root volume).**
