# Supplementary material for: Genotypic variation in root architectural traits under contrasting phosphorus levels in Mediterranean and Indian origin lentil genotypes
Source: PeerJ. 2022 Mar 10;10:e12766. doi: 10.7717/peerj.12766 (PMC8918163; doi:10.7717/peerj.12766)
Supplement: Supplemental Information 8 — Contrasting genotypes identified using Comprehensive phosphorus efficiency measurement value. TSA, total root surface area; PRL, primary root length; RAD, root average diameter; TRL, total root length; TRF, total root forks: TRT, total root tips; TRV, total root volume. DP, deficit phosphorus: SP, sufficient phosphorus. [file peerj-10-12766-s008.docx]

**Supplementary Table 8. Loading factors of 7 principal components (Eigen vectors) under deficit P conditions.**

|  | **PC 1** | **PC 2** | **PC 3** | **PC 4** | **PC 5** | **PC 6** | **PC 7** |
| --- | --- | --- | --- | --- | --- | --- | --- |
| **A** | 0.190 | **0.790** | 0.310 | 0.074 | **0.997** | -0.001 | 0.001 |
| **B** | **0.400** | -0.150 | -0.330 | -0.035 | 0.003 | 0.000 | 0.000 |
| **C** | **0.440** | -0.010 | 0.220 | **0.996** | -0.074 | -0.005 | 0.000 |
| **D** | -0.250 | -0.280 | 0.810 | 0.001 | -0.001 | 0.081 | **0.997** |
| **E** | **0.450** | -0.290 | 0.260 | 0.005 | 0.001 | **0.997** | -0.081 |
| **F** | 0.400 | 0.300 | 0.090 | -0.035 | -0.010 | 0.000 | 0.000 |
| **G** | **0.440** | -0.320 | 0.070 | -0.007 | 0.002 | 0.000 | 0.000 |

**Where A: TRL (total root length); B: PRL (primary root length); C: RAD (root average diameter); D: TSA (total root surface area); E: TRF (total root forks); F: TRT (total root tips); G: TRV (total root volume).**
