# Supplementary material for: Genotypic variation in root architectural traits under contrasting phosphorus levels in Mediterranean and Indian origin lentil genotypes
Source: PeerJ. 2022 Mar 10;10:e12766. doi: 10.7717/peerj.12766 (PMC8918163; doi:10.7717/peerj.12766)
Supplement: Supplemental Information 9 — DP, deficit phosphorus; SP, sufficient phosphorus; PCI, principal component one; PCII, principal component two; PCIII, principal component three; TRL, total root length; PRL, primary root length; TSA, total root surface area; TRV, total root volume; TRF, total root forks; RAD, root average diameter; TRT, total root tips. [file peerj-10-12766-s009.docx]

**Supplementary Table 9. The highly contributing traits, Eigen values, % variance, and cumulative % variance in 2 phosphorus conditions indicated by principal component analysis.**

| **Traits** | **SP** | | | **DP** | | |
| --- | --- | --- | --- | --- | --- | --- |
|  | **PC I** | **PC II** | **PC III** | **PC I** | **PC II** | **PC III** |
| **PRL** | 0.35 | 0.15 | -0.62 | 0.19 | **0.79** | **0.31** |
| **TRL** | 0.32 | -0.58 | 0.21 | 0.40 | -0.15 | -0.33 |
| **TSA** | **0.48** | -0.11 | 0.16 | **0.44** | -0.01 | 0.22 |
| **RAD** | -0.08 | **0.72** | 0.19 | -0.25 | -0.28 | **0.81** |
| **TRV** | **0.47** | 0.20 | 0.30 | 0.45 | -0.29 | 0.26 |
| **TRT** | 0.38 | 0.11 | **-0.54** | 0.40 | **0.30** | 0.09 |
| **TRF** | 0.41 | 0.25 | 0.36 | **0.44** | -0.32 | 0.07 |
| **Eigen values** | 3.21 | 1.54 | 1.00 | 3.56 | 1.07 | 0.95 |
| **% Variance** | 45.89 | 21.99 | 14.32 | 50.90 | 15.29 | 13.52 |
| **Cumulative % Variance** | 45.89 | 67.88 | 82.22 | 50.90 | 66.18 | 79.70 |
| **Highly contributing ones** | TSA,TRV | RAD,TRT | TRT,TRV | TRV,TSA | PRL,TRT | RAD,PRL |

DP, deficit phosphorus; SP, sufficient phosphorus; PCI, principal component one; PCII, principal component two; PCIII, principal component three; TRL, total root length; PRL, primary root length; TSA, total root surface area; TRV, total root volume; TRF, total root forks; RAD, root average diameter; TRT, total root tips.
