# Supplementary material for: Genotypic variation in root architectural traits under contrasting phosphorus levels in Mediterranean and Indian origin lentil genotypes
Source: PeerJ. 2022 Mar 10;10:e12766. doi: 10.7717/peerj.12766 (PMC8918163; doi:10.7717/peerj.12766)
Supplement: Supplemental Information 10 — Contrasting genotypes identified using Comprehensive phosphorus efficiency measurement value. TSA, total root surface area; PRL, primary root length; RAD, root average diameter; TRL, total root length; TRF, total root forks: TRT, total root tips; TRV, total root volume. DP, deficit phosphorus: SP, sufficient phosphorus. [file peerj-10-12766-s010.docx]

**Supplementary Table 10. Top eight and bottom eight lentil genotypes based on the CPEM values under sufficient and deficit phosphorus constraints.**

| **Top eight genotypes** | | **Root architectural traits** | | | | | | | | | | | | | | |
| --- | --- | --- | --- | --- | --- | --- | --- | --- | --- | --- | --- | --- | --- | --- | --- | --- |
| **Genotypes** | **Class** | **CPEM** | **PRL** | | **TRL** | | **TSA** | | **RAD** | | **TRV** | | **TRT** | | **TRF** | |
|  |  |  | **SP** | **DP** | **SP** | **DP** | **SP** | **DP** | **SP** | **DP** | **SP** | **DP** | **SP** | **DP** | **SP** | **DP** |
| PLL 18-9 | ABL | 3.412 | 20.00 | 38.33 | 590.21 | 468.51 | 32.15 | 46.77 | 0.24 | 0.42 | 0.34 | 1.51 | 148.67 | 374.33 | 373.33 | 2941.00 |
| PLS 18-01 | ABL | 2.267 | 12.67 | 30.33 | 734.01 | 632.77 | 62.44 | 40.29 | 0.26 | 0.38 | 0.46 | 1.61 | 232.00 | 629.33 | 442.67 | 3125.33 |
| PLL 18-25 | ABL | 2.264 | 20.00 | 28.67 | 590.21 | 363.68 | 32.15 | 27.50 | 0.24 | 0.55 | 0.34 | 0.41 | 148.67 | 319.67 | 373.33 | 379.33 |
| PLS 18-23 | ABL | 2.202 | 19.00 | 28.33 | 669.86 | 462.48 | 35.07 | 32.55 | 0.23 | 0.38 | 0.47 | 1.50 | 241.33 | 441.00 | 515.33 | 1253.00 |
| PLL 18-7 | ABL | 1.901 | 12.67 | 29.33 | 657.49 | 470.33 | 23.14 | 34.03 | 0.25 | 0.37 | 0.24 | 0.74 | 82.33 | 360.67 | 274.33 | 821.67 |
| IPL 406 | RV | 1.900 | 15.67 | 35.00 | 335.10 | 163.40 | 13.37 | 52.86 | 0.45 | 0.53 | 0.19 | 0.64 | 87.33 | 424.00 | 202.33 | 450.67 |
| PLS 406-1 | ABL | 1.662 | 19.67 | 28.33 | 814.48 | 751.43 | 141.17 | 115.02 | 0.29 | 0.35 | 0.36 | 1.13 | 342.33 | 520.00 | 449.00 | 1360.33 |
| L 4698 | ABL | 1.506 | 23.33 | 33.00 | 292.44 | 217.00 | 40.37 | 57.58 | 0.38 | 0.65 | 0.41 | 0.76 | 185.33 | 157.33 | 548.33 | 787.00 |
| **Bottom eight** | | **Root architectural traits** | | | | | | | | | | | | | | |
| **Genotypes** | **Class** | **CPEM** | **PRL** | | **TRL** | | **TSA** | | **RAD** | | **TRV** | | **TRT** | | **TRF** | |
|  |  |  | **SP** | **DP** | **SP** | **DP** | **SP** | **DP** | **SP** | **DP** | **SP** | **DP** | **SP** | **DP** | **SP** | **DP** |
| IG 112131 | EG | 0.410 | 28.00 | 32.33 | 279.40 | 140.20 | 42.43 | 18.87 | 0.43 | 0.43 | 0.42 | 0.16 | 267.00 | 170.67 | 368.67 | 246.67 |
| P 560206 | EG | 0.428 | 19.00 | 33.33 | 355.73 | 245.77 | 59.57 | 62.76 | 0.48 | 0.45 | 0.67 | 0.25 | 275.33 | 695.00 | 547.33 | 1176.00 |
| IG 334 | EG | 0.442 | 22.33 | 30.33 | 285.90 | 154.99 | 42.93 | 33.89 | 0.45 | 0.46 | 0.63 | 0.26 | 374.33 | 259.33 | 775.00 | 198.00 |
| L 11-231 | ABL | 0.443 | 19.67 | 34.00 | 199.09 | 137.75 | 26.46 | 17.40 | 0.43 | 0.43 | 0.37 | 0.19 | 238.33 | 133.33 | 456.67 | 152.67 |
| PLS 18-67 | ABL | 0.453 | 20.67 | 31.00 | 234.37 | 164.23 | 26.39 | 12.77 | 0.40 | 0.41 | 0.39 | 0.11 | 232.33 | 132.00 | 895.67 | 215.33 |
| PLS 18-57 | ABL | 0.473 | 20.00 | 25.67 | 219.79 | 175.18 | 35.39 | 23.98 | 0.46 | 0.47 | 0.47 | 0.33 | 170.33 | 156.00 | 364.67 | 429.67 |
| L 4603 | ABL | 0.477 | 22.33 | 32.33 | 486.00 | 334.57 | 89.26 | 47.98 | 0.43 | 0.46 | 1.23 | 0.57 | 575.33 | 270.67 | 2541.33 | 872.00 |
| L 11-234 | ABL | 0.479 | 20.67 | 30.33 | 194.37 | 46.86 | 26.39 | 6.31 | 0.40 | 0.43 | 0.39 | 0.06 | 232.33 | 98.67 | 895.67 | 52.33 |

Contrasting genotypes identified using Comprehensive phosphorus efficiency measurement value. TSA, total root surface area; PRL, primary root length; RAD, root average diameter; TRL, total root length; TRF, total root forks: TRT, total root tips; TRV, total root volume. DP, deficit phosphorus: SP, sufficient phosphorus.
