# Supplementary figures and images for: Genotypic variation in root architectural traits under contrasting phosphorus levels in Mediterranean and Indian origin lentil genotypes
Source: PeerJ. 2022 Mar 10;10:e12766. doi: 10.7717/peerj.12766 (PMC8918163; doi:10.7717/peerj.12766)

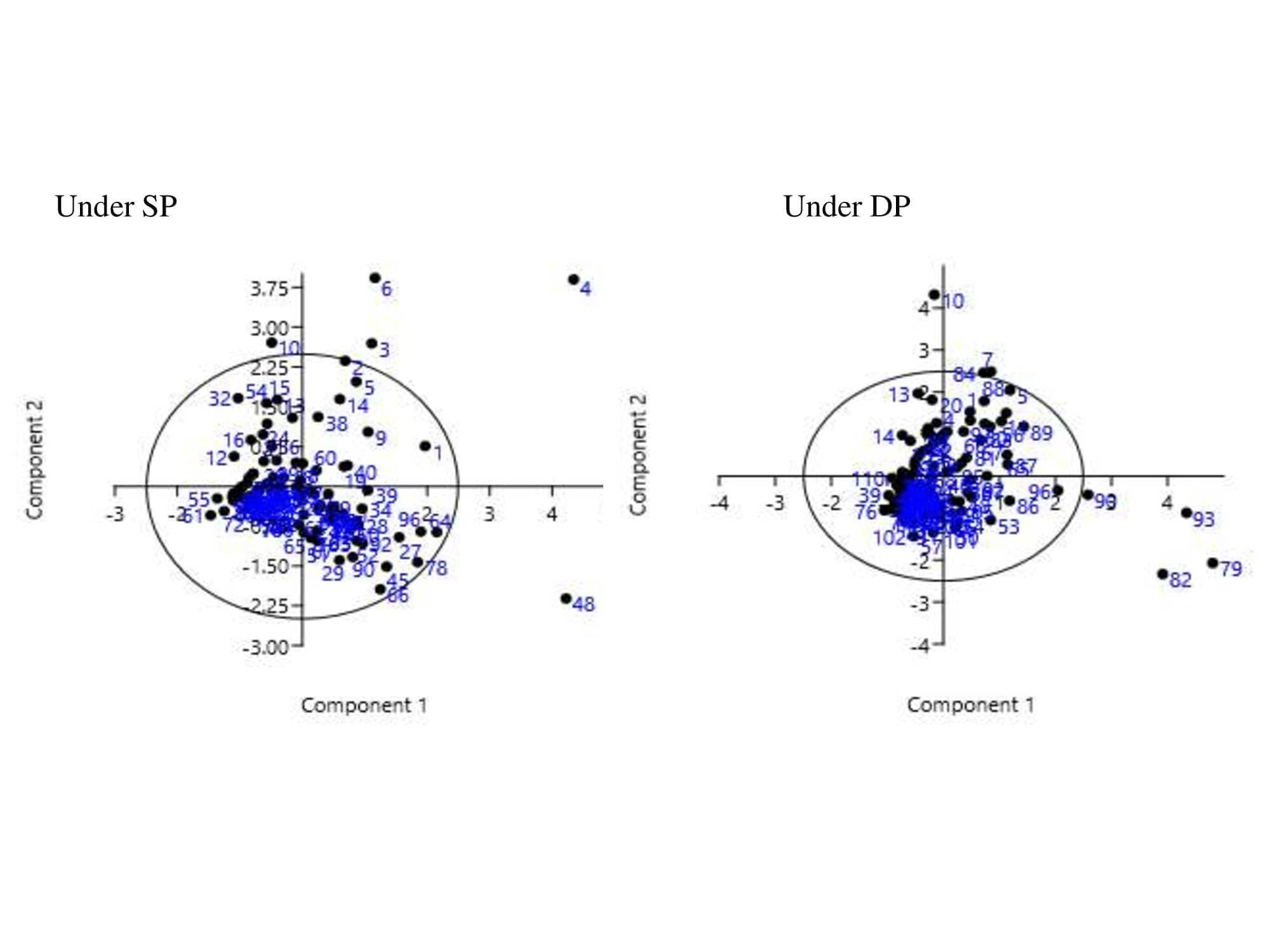

Supplement: Supplemental Information 11 — (Refer the supplementary table 1 for genotype numbers.) [file peerj-10-12766-s011.jpg]

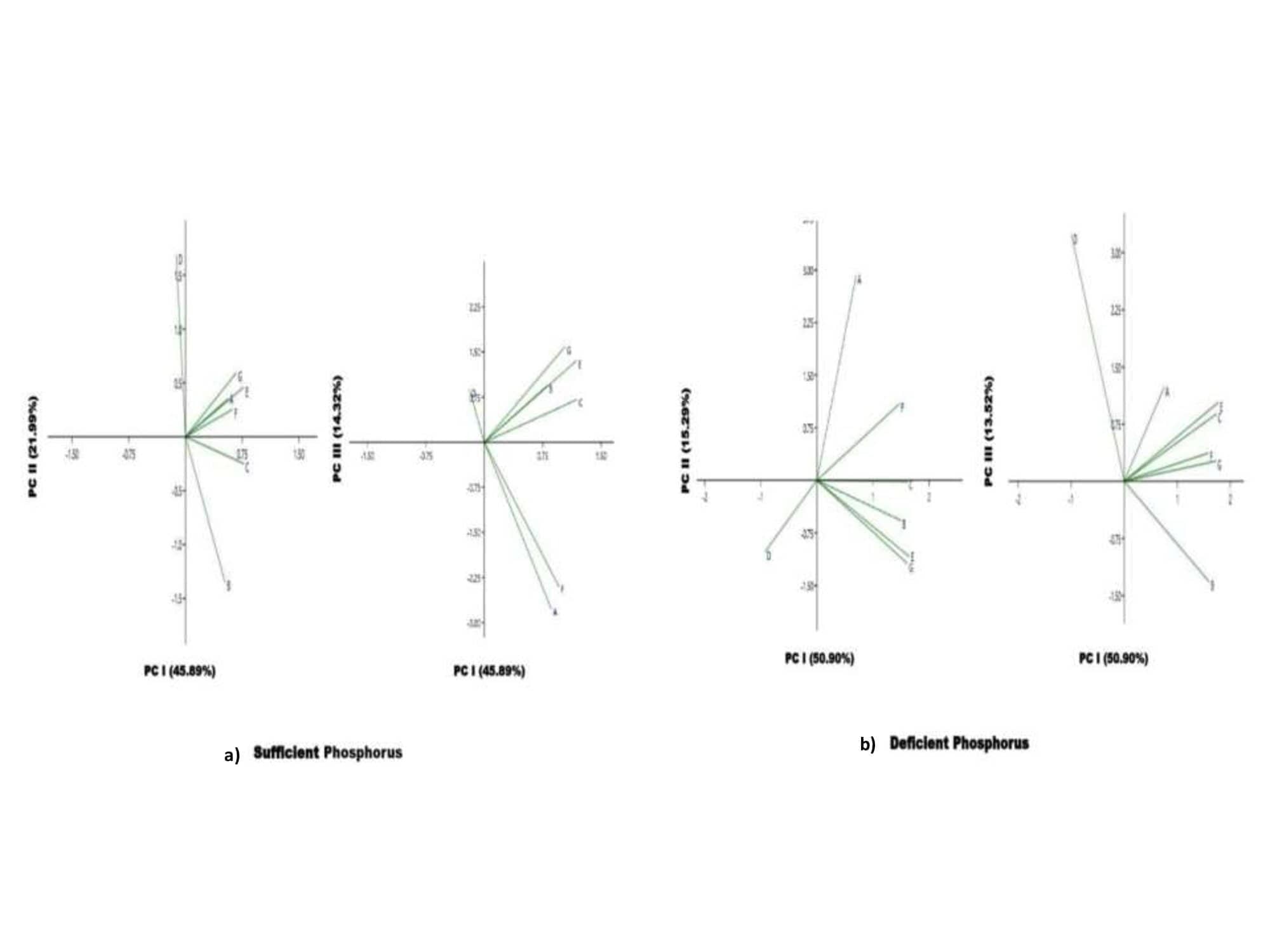

Supplement: Supplemental Information 12 — (Where, A:TRL-total root length; B:PRL-primary root length; C:TSA -total root surface area -; D:TRV, total root volume; E: RAD- root average diameter;F: TRF-total root forks; G: TRT-total root tips.) [file peerj-10-12766-s012.jpg]

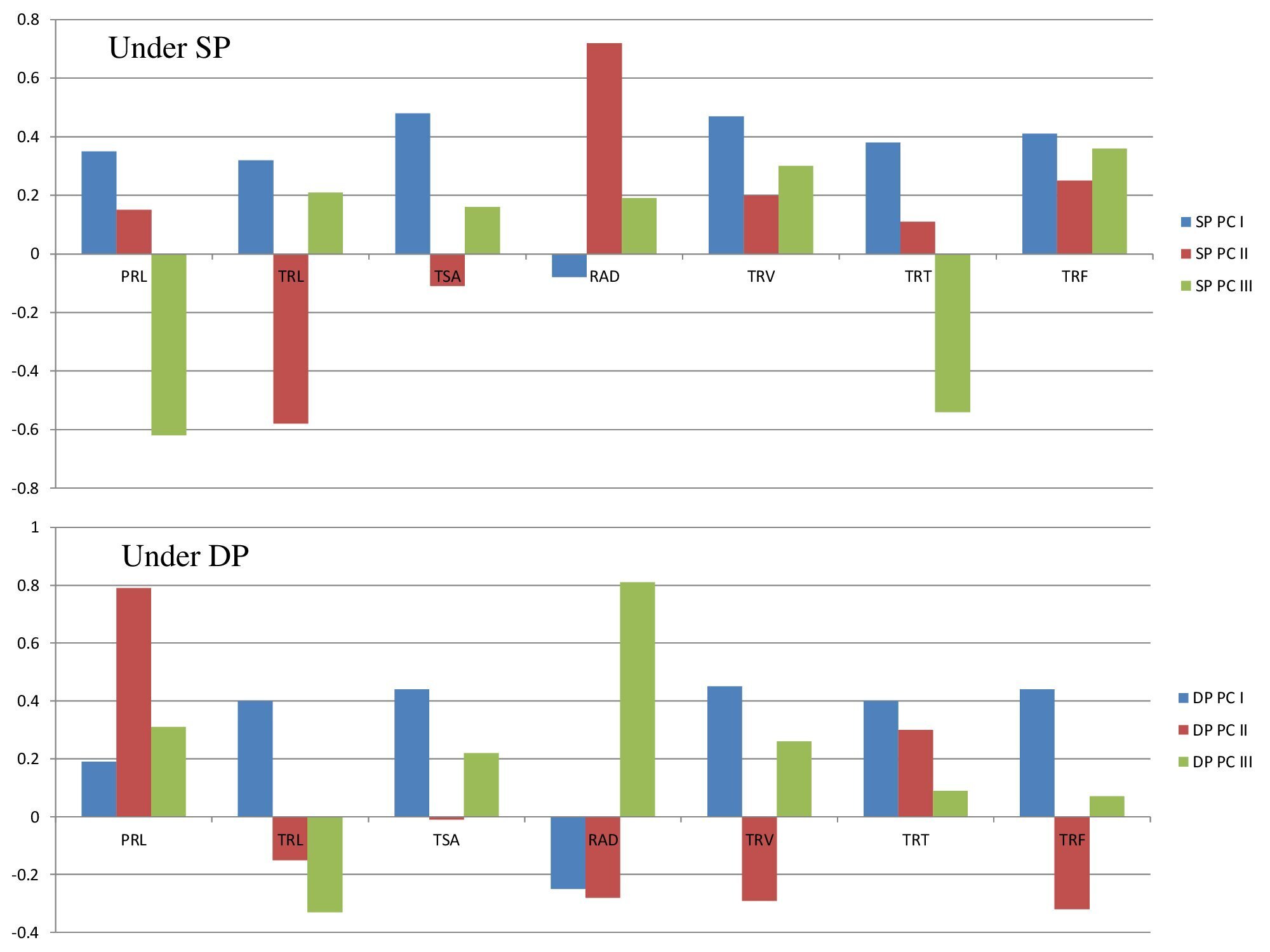

Supplement: Supplemental Information 13 — (Where, TRL-total root length; PRL-primary root length; TSA -total root surface area -; total root volume-TRV; RAD- root average diameter; TRF-total root forks; TRT-total root tips.) [file peerj-10-12766-s013.jpg]

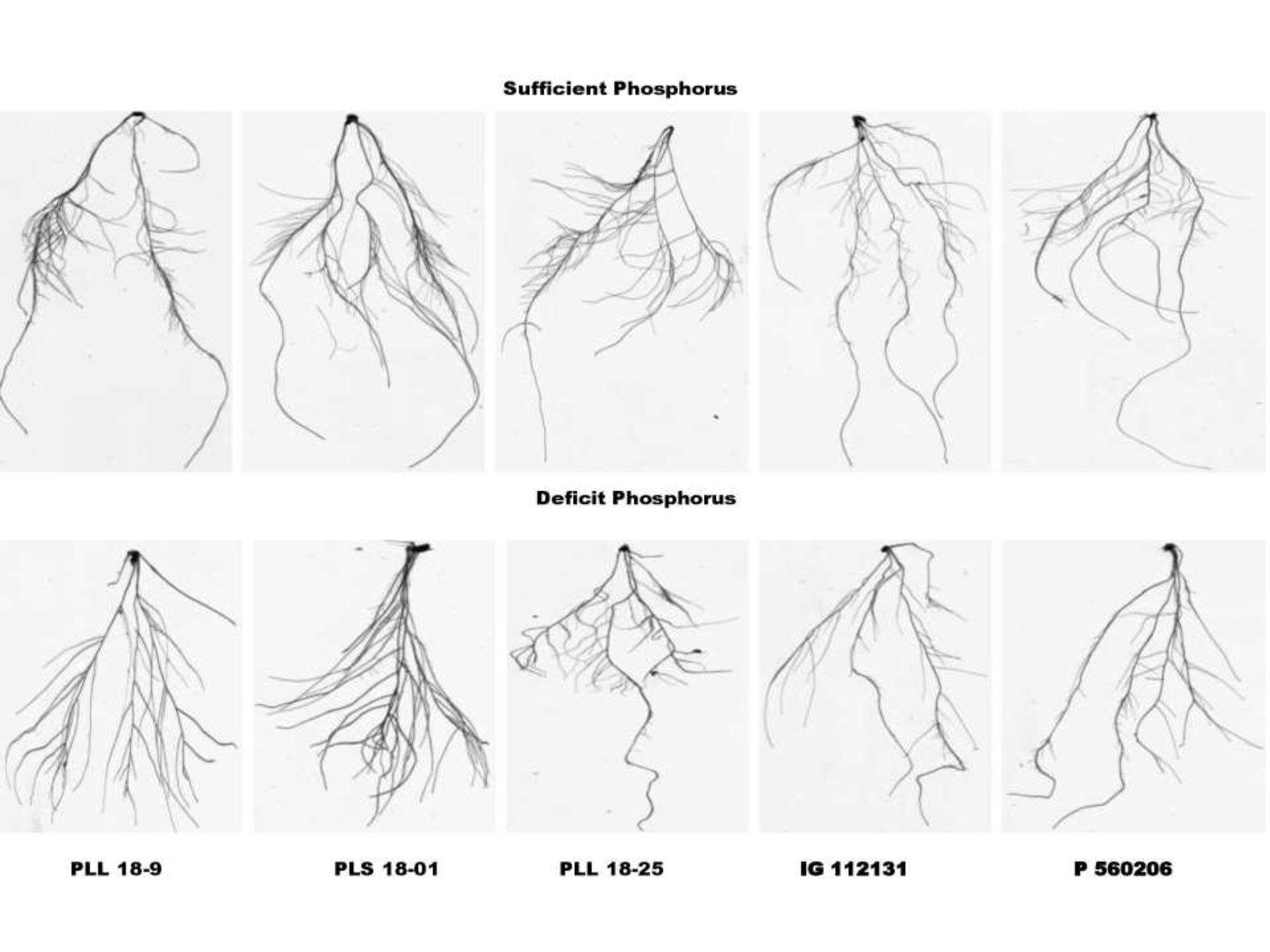

Supplement: Supplemental Information 14 — Names of the genotypes [file peerj-10-12766-s014.jpg]

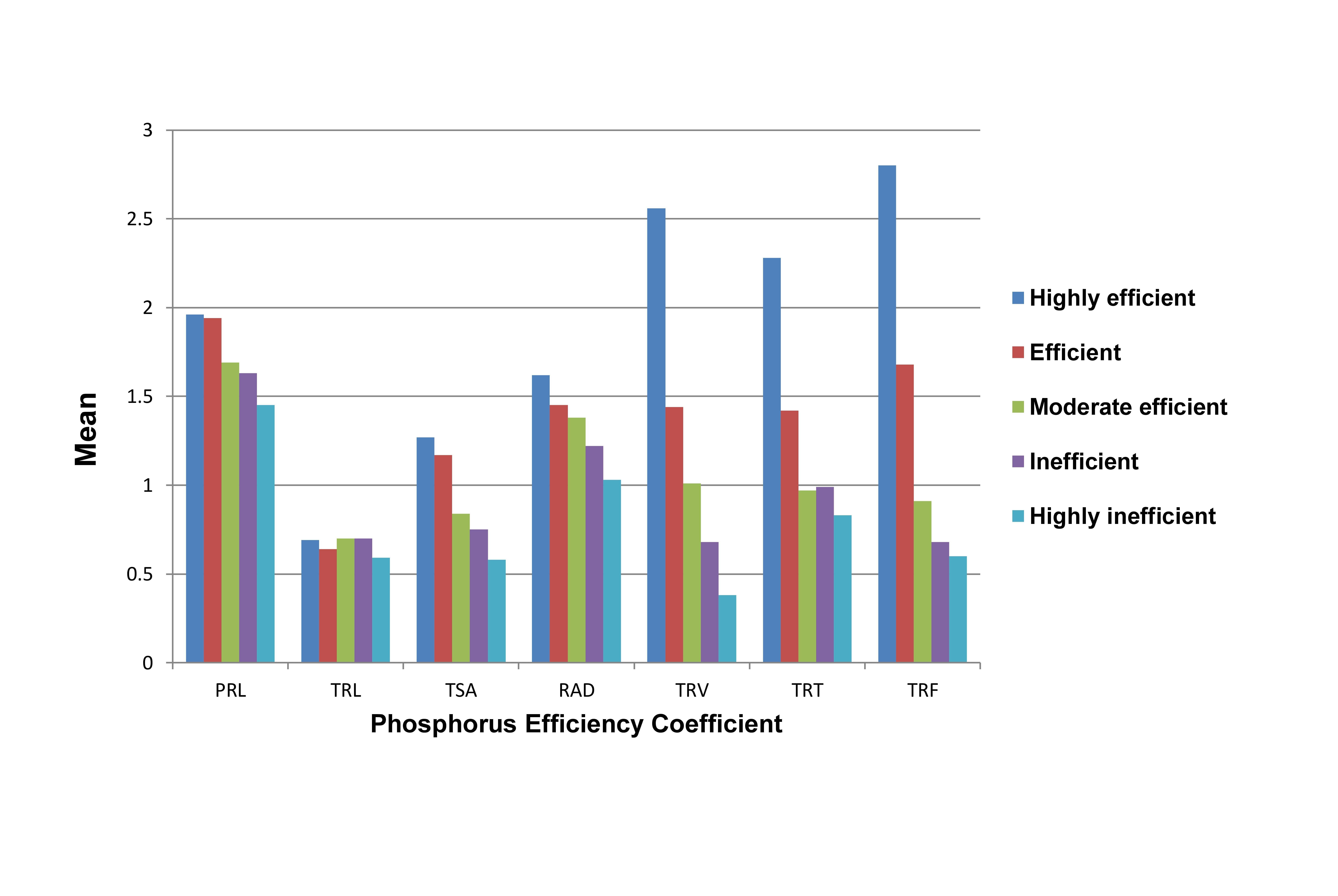

Supplement: Supplemental Information 15 — (Where, TRL-total root length; PRL-primary root length; TSA -total root surface area -; total root volume-TRV; RAD- root average diameter; TRF-total root forks; TRT-total root tips. Lentil genotypes classified into highly efficient to highly inefficient are represented by groups in different colors. For classes 1, 2, 3, 4, and 5, N = 11, 15, 30, 45, and 9 respectively for each class.) [file peerj-10-12766-s015.jpg]
