# Supplementary material for: Genotypic variation in root architectural traits under contrasting phosphorus levels in Mediterranean and Indian origin lentil genotypes
Source: PeerJ. 2022 Mar 10;10:e12766. doi: 10.7717/peerj.12766 (PMC8918163; doi:10.7717/peerj.12766)
Supplement: Supplemental Information 16 — Raw data tables of main root traits and root diameter classes [file peerj-10-12766-s016.docx]

Raw data of main root traits under P + conditions:

| Genotypes | Name | PRL | TRL | TSA | RAD | TRV | TRT | TRF |
| --- | --- | --- | --- | --- | --- | --- | --- | --- |
| 1 | HM-1 | 28.67 | 673.77 | 86.32 | 0.33 | 0.96 | 823.33 | 1368.67 |
| 2 | L 4649 | 25.67 | 519.93 | 73.08 | 0.31 | 0.77 | 993.33 | 735.33 |
| 3 | IG 69568 | 31.33 | 649.09 | 85.03 | 0.33 | 0.65 | 1122.33 | 855.67 |
| 4 | IC 321808 | 31.00 | 654.39 | 98.95 | 0.33 | 1.08 | 1937.00 | 2002.33 |
| 5 | IC 560135 | 23.00 | 645.78 | 42.72 | 0.35 | 0.49 | 918.67 | 828.33 |
| 6 | IG 136607 | 35.33 | 475.12 | 59.91 | 0.30 | 0.62 | 1434.33 | 779.33 |
| 7 | IG-Y- 50 | 27.67 | 446.43 | 69.12 | 0.34 | 0.71 | 350.33 | 787.67 |
| 8 | IG 134349 | 29.00 | 345.26 | 51.28 | 0.34 | 0.61 | 464.00 | 672.33 |
| 9 | LL 699 | 21.00 | 522.27 | 79.91 | 0.36 | 0.80 | 750.67 | 1003.67 |
| 10 | IC 268238 | 19.00 | 269.49 | 51.71 | 0.33 | 0.53 | 908.00 | 272.33 |
| 11 | ILWL-95 | 21.00 | 372.44 | 28.77 | 0.32 | 0.34 | 272.67 | 517.67 |
| 12 | ILWL-15 | 14.00 | 120.57 | 14.25 | 0.28 | 0.15 | 341.00 | 256.67 |
| 13 | IG 73798 | 28.67 | 401.36 | 66.87 | 0.33 | 0.76 | 624.00 | 519.67 |
| 14 | EC 78472 | 27.00 | 326.03 | 59.59 | 0.36 | 0.77 | 841.67 | 792.00 |
| 15 | L5126 | 21.33 | 435.66 | 35.71 | 0.30 | 0.34 | 657.33 | 390.00 |
| 16 | Fasciated mutant | 22.67 | 306.44 | 33.27 | 0.36 | 0.40 | 431.33 | 312.00 |
| 17 | L 4618 | 25.00 | 420.18 | 63.29 | 0.35 | 0.90 | 345.67 | 1004.00 |
| 18 | IC 560181 | 27.00 | 396.69 | 49.27 | 0.30 | 0.51 | 443.00 | 622.67 |
| 19 | IG 129185 | 22.00 | 386.99 | 52.95 | 0.32 | 0.58 | 556.33 | 930.00 |
| 20 | IG 49 | 29.67 | 388.14 | 35.32 | 0.35 | 0.45 | 364.00 | 420.33 |
| 21 | IG 112078 | 18.00 | 301.17 | 28.48 | 0.31 | 0.68 | 488.33 | 378.00 |
| 22 | IG 73920 | 23.67 | 415.93 | 51.90 | 0.32 | 0.56 | 320.67 | 717.67 |
| 23 | P 3233 | 15.67 | 287.23 | 46.89 | 0.40 | 0.53 | 317.67 | 579.00 |
| 24 | IG 560183 | 21.33 | 436.94 | 37.19 | 0.33 | 0.34 | 527.00 | 369.33 |
| 25 | IPL 406 | 17.00 | 290.11 | 25.34 | 0.52 | 0.28 | 140.00 | 300.67 |
| 26 | IG 560206 | 20.00 | 305.70 | 53.84 | 0.32 | 0.58 | 251.00 | 589.33 |
| 27 | L 4076 | 20.33 | 457.57 | 73.28 | 0.34 | 0.83 | 392.67 | 1392.67 |
| 28 | IG 134656 | 23.00 | 457.04 | 59.15 | 0.35 | 0.67 | 334.33 | 1115.67 |
| 29 | IG 560185 | 21.67 | 305.55 | 25.20 | 0.35 | 0.33 | 155.67 | 1078.33 |
| 30 | L 4698 | 23.33 | 292.44 | 40.37 | 0.38 | 0.41 | 185.33 | 548.33 |
| 31 | PL 97 | 22.33 | 503.07 | 65.46 | 0.33 | 0.73 | 316.67 | 1014.33 |
| 32 | P 2208 | 21.00 | 507.35 | 84.35 | 0.36 | 0.34 | 547.33 | 133.00 |
| 33 | ILL 10821 | 20.00 | 388.73 | 64.49 | 0.39 | 0.76 | 306.67 | 1081.67 |
| 34 | IG 936 | 19.67 | 286.28 | 32.21 | 0.32 | 0.35 | 439.67 | 1130.67 |
| 35 | IPL 321 | 20.00 | 362.14 | 67.19 | 0.37 | 0.71 | 348.00 | 947.00 |
| 36 | P 2116 | 21.33 | 382.55 | 47.87 | 0.40 | 0.66 | 447.33 | 444.67 |
| 37 | IG 334 | 22.33 | 285.90 | 42.93 | 0.45 | 0.63 | 374.33 | 775.00 |
| 38 | IG 560157 | 18.00 | 660.17 | 106.33 | 0.39 | 0.37 | 661.00 | 648.67 |
| 39 | IG 568229 | 13.67 | 382.31 | 56.49 | 0.36 | 0.66 | 519.67 | 1121.67 |
| 40 | IG 130033 | 25.67 | 492.23 | 35.31 | 0.38 | 0.42 | 557.00 | 939.67 |
| 41 | IG 112131 | 28.00 | 279.40 | 42.43 | 0.43 | 0.42 | 267.00 | 368.67 |
| 42 | LC 300-19 | 17.33 | 459.39 | 67.04 | 0.38 | 0.84 | 319.67 | 842.33 |
| 43 | Global Mutant | 21.00 | 162.47 | 23.56 | 0.32 | 0.23 | 258.00 | 381.67 |
| 44 | ILL10832 | 23.33 | 164.25 | 23.07 | 0.38 | 0.30 | 216.67 | 778.33 |
| 45 | EC 78518 | 20.00 | 421.00 | 61.39 | 0.43 | 0.84 | 238.67 | 1371.33 |
| 46 | IC 560162 | 23.33 | 477.25 | 47.04 | 0.41 | 0.49 | 284.00 | 937.00 |
| 47 | P 2130 | 23.67 | 391.30 | 41.23 | 0.37 | 0.45 | 340.67 | 669.00 |
| 48 | L 4603 | 22.33 | 486.00 | 89.26 | 0.43 | 1.23 | 575.33 | 2541.33 |
| 49 | P 8116 | 13.67 | 322.78 | 36.03 | 0.42 | 0.65 | 303.00 | 932.33 |
| 50 | L 4705 | 18.67 | 290.28 | 56.85 | 0.44 | 0.66 | 372.67 | 1117.33 |
| 51 | IG 56968 | 14.00 | 273.90 | 45.01 | 0.45 | 0.60 | 182.67 | 912.67 |
| 52 | IG 129302 | 18.00 | 277.41 | 44.97 | 0.41 | 0.76 | 289.00 | 1153.67 |
| 53 | P 3226 | 17.33 | 263.97 | 27.44 | 0.41 | 0.54 | 184.00 | 553.00 |
| 54 | P 560183 | 12.33 | 189.29 | 22.46 | 0.38 | 0.27 | 646.67 | 356.67 |
| 55 | IPL 406-1 | 15.67 | 335.10 | 13.37 | 0.45 | 0.19 | 87.33 | 202.33 |
| 56 | P 560206 | 19.00 | 355.73 | 59.57 | 0.48 | 0.67 | 275.33 | 547.33 |
| 57 | L 4076-1 | 13.33 | 239.84 | 22.36 | 0.41 | 0.29 | 212.33 | 342.67 |
| 58 | IG 134656 | 21.33 | 513.17 | 45.42 | 0.43 | 0.65 | 208.00 | 429.00 |
| 59 | P 560185 | 14.67 | 212.32 | 28.56 | 0.39 | 0.37 | 371.67 | 595.00 |
| 60 | PL 97-1 | 19.67 | 337.37 | 61.16 | 0.46 | 0.92 | 472.00 | 770.67 |
| 61 | ILWL 147 | 12.67 | 71.21 | 5.35 | 0.32 | 0.07 | 33.33 | 221.00 |
| 62 | IG 129313 | 20.33 | 384.84 | 57.64 | 0.43 | 0.64 | 329.00 | 936.67 |
| 63 | LC 282-1077 | 19.67 | 296.40 | 40.32 | 0.41 | 0.53 | 256.00 | 776.33 |
| 64 | IC 560297 | 21.00 | 409.01 | 62.94 | 0.41 | 0.80 | 522.67 | 1625.00 |
| 65 | IC 27986 | 16.67 | 410.36 | 35.08 | 0.37 | 0.49 | 164.33 | 793.67 |
| 66 | IC 346092 | 16.33 | 326.75 | 52.45 | 0.47 | 0.83 | 137.67 | 1382.67 |
| 67 | P 43120 | 21.00 | 445.97 | 64.74 | 0.52 | 0.62 | 153.67 | 843.67 |
| 68 | P 43103 | 20.00 | 219.79 | 35.39 | 0.46 | 0.47 | 170.33 | 364.67 |
| 69 | P 16214 | 12.67 | 172.36 | 28.23 | 0.40 | 0.37 | 166.67 | 433.00 |
| 70 | P 16213 | 16.67 | 173.27 | 25.36 | 0.39 | 0.28 | 150.00 | 518.33 |
| 71 | L 11-280 | 12.00 | 212.38 | 32.89 | 0.40 | 0.48 | 226.67 | 555.33 |
| 72 | L 11-279 | 19.33 | 158.67 | 15.09 | 0.31 | 0.33 | 75.33 | 288.00 |
| 73 | L 11-248 | 17.67 | 196.46 | 24.17 | 0.33 | 0.26 | 167.33 | 518.33 |
| 74 | L 11-244 | 17.67 | 208.26 | 13.68 | 0.34 | 0.15 | 165.00 | 307.67 |
| 75 | L 11-243 | 19.00 | 284.51 | 43.70 | 0.37 | 0.23 | 296.00 | 388.00 |
| 76 | L 11-234 | 20.67 | 194.37 | 26.39 | 0.40 | 0.39 | 232.33 | 895.67 |
| 77 | L 11-231 | 19.67 | 199.09 | 26.46 | 0.43 | 0.37 | 238.33 | 456.67 |
| 78 | PLL-18-1 | 14.67 | 850.32 | 68.75 | 0.27 | 0.75 | 283.67 | 1508.67 |
| 79 | PLL 18-2 | 21.00 | 824.35 | 77.08 | 0.31 | 0.95 | 338.33 | 831.67 |
| 80 | PLL 18-5 | 21.33 | 921.95 | 87.89 | 0.28 | 0.77 | 289.00 | 880.00 |
| 81 | PLL 18-7 | 12.67 | 657.49 | 23.14 | 0.25 | 0.24 | 82.33 | 274.33 |
| 82 | PLL 18-9 | 20.00 | 590.21 | 32.15 | 0.24 | 0.34 | 148.67 | 373.33 |
| 83 | PLL 18-11 | 12.00 | 781.50 | 30.73 | 0.30 | 0.40 | 147.33 | 289.67 |
| 84 | PLL 18-12 | 22.67 | 675.74 | 79.94 | 0.26 | 0.67 | 267.00 | 813.33 |
| 85 | PLL 18-14 | 18.00 | 795.48 | 48.31 | 0.26 | 0.46 | 242.33 | 521.33 |
| 86 | PLS-18-21 | 17.67 | 623.66 | 43.37 | 0.30 | 0.44 | 241.00 | 451.00 |
| 87 | PLS -18-23 | 19.00 | 669.86 | 35.07 | 0.23 | 0.47 | 241.33 | 515.33 |
| 88 | PLS 18-25 | 15.67 | 851.11 | 47.39 | 0.25 | 0.52 | 259.00 | 455.67 |
| 89 | PLS 18-32 | 19.67 | 814.48 | 141.17 | 0.29 | 0.36 | 342.33 | 449.00 |
| 90 | PLS 18-33 | 15.00 | 789.98 | 57.62 | 0.28 | 0.56 | 139.67 | 1101.00 |
| 91 | PLS 18-36 | 21.00 | 679.22 | 35.65 | 0.25 | 0.28 | 147.33 | 404.33 |
| 92 | PLS 18-44 | 26.00 | 786.13 | 72.94 | 0.30 | 0.53 | 225.00 | 1139.00 |
| 93 | PLS 18-01 | 12.67 | 734.01 | 62.44 | 0.26 | 0.46 | 232.00 | 442.67 |
| 94 | PLS 18-48 | 18.00 | 439.87 | 36.08 | 0.25 | 0.35 | 164.67 | 436.33 |
| 95 | PLS 18-50 | 12.00 | 462.98 | 44.74 | 0.26 | 0.35 | 210.00 | 420.67 |
| 96 | PLS 18-52 | 21.67 | 755.67 | 55.82 | 0.26 | 0.87 | 436.00 | 1485.00 |
| 97 | PLS 18-53 | 16.33 | 720.98 | 74.19 | 0.27 | 0.74 | 336.33 | 644.67 |
| 98 | PLS 18-57 | 20.00 | 219.79 | 35.39 | 0.46 | 0.47 | 170.33 | 364.67 |
| 99 | PLS 18-58 | 12.67 | 205.69 | 28.23 | 0.40 | 0.37 | 166.67 | 433.00 |
| 100 | PLS 18-60 | 16.67 | 173.27 | 26.36 | 0.39 | 0.28 | 150.00 | 518.33 |
| 101 | PLS 18-62 | 12.00 | 232.38 | 32.89 | 0.40 | 0.48 | 226.67 | 555.33 |
| 102 | PLS 18-64 | 17.67 | 266.46 | 24.17 | 0.33 | 0.26 | 167.33 | 518.33 |
| 103 | PLS 18-65 | 17.67 | 308.26 | 13.68 | 0.34 | 0.15 | 165.00 | 307.67 |
| 104 | PLS 18-66 | 19.00 | 284.51 | 43.70 | 0.37 | 0.23 | 296.00 | 388.00 |
| 105 | PLS 18-67 | 20.67 | 234.37 | 26.39 | 0.40 | 0.39 | 232.33 | 895.67 |
| 106 | PLL 18-22 | 19.67 | 199.09 | 29.46 | 0.43 | 0.37 | 238.33 | 456.67 |
| 107 | PLL 18-23 | 21.33 | 728.62 | 87.89 | 0.28 | 0.77 | 289.00 | 880.00 |
| 108 | PLL 18-24 | 12.67 | 624.15 | 43.14 | 0.25 | 0.24 | 82.33 | 274.33 |
| 109 | PLL 18-25 | 20.00 | 590.21 | 32.15 | 0.24 | 0.34 | 148.67 | 373.33 |
| 110 | PLL 18-26 | 12.00 | 714.84 | 30.73 | 0.30 | 0.40 | 147.33 | 289.67 |

Raw data of main root traits under P - conditions:

| Genotypes | Name | PRL | TRL | TSA | RAD | TRV | TRT | TRF |
| --- | --- | --- | --- | --- | --- | --- | --- | --- |
| 1 | HM-1 | 33.00 | 408.80 | 56.35 | 0.43 | 0.63 | 588.00 | 827.33 |
| 2 | L 4649 | 41.67 | 393.40 | 56.17 | 0.43 | 0.63 | 413.67 | 548.67 |
| 3 | IG 69568 | 39.00 | 430.90 | 43.94 | 0.41 | 0.45 | 351.00 | 475.00 |
| 4 | IC 321808 | 45.00 | 430.75 | 57.91 | 0.44 | 0.54 | 434.67 | 603.00 |
| 5 | IC 560135 | 33.00 | 449.40 | 90.46 | 0.47 | 1.13 | 764.33 | 1219.33 |
| 6 | IG 136607 | 44.00 | 306.42 | 34.42 | 0.42 | 0.26 | 431.33 | 441.00 |
| 7 | IG-Y- 50 | 43.00 | 404.19 | 53.48 | 0.42 | 0.88 | 814.00 | 1013.00 |
| 8 | IG 134349 | 35.00 | 268.10 | 33.20 | 0.43 | 0.40 | 511.67 | 504.33 |
| 9 | LL 699 | 40.33 | 446.41 | 16.94 | 0.42 | 0.27 | 255.67 | 329.33 |
| 10 | IC 268238 | 29.33 | 215.27 | 33.69 | 0.45 | 0.34 | 1072.67 | 386.00 |
| 11 | ILWL-95 | 50.67 | 220.89 | 64.65 | 0.42 | 0.65 | 753.00 | 1221.00 |
| 12 | ILWL-15 | 26.67 | 76.64 | 10.32 | 0.46 | 0.14 | 165.67 | 231.67 |
| 13 | IG 73798 | 42.33 | 361.16 | 39.47 | 0.37 | 0.44 | 551.00 | 280.67 |
| 14 | EC 78472 | 42.00 | 285.88 | 33.77 | 0.39 | 0.26 | 363.67 | 157.33 |
| 15 | L5126 | 40.33 | 343.76 | 51.57 | 0.40 | 0.42 | 336.00 | 239.00 |
| 16 | Fasciated mutant | 36.67 | 249.09 | 33.77 | 0.43 | 0.51 | 252.33 | 316.00 |
| 17 | L 4618 | 40.00 | 320.29 | 47.91 | 0.46 | 0.63 | 444.67 | 414.33 |
| 18 | IC 560181 | 34.33 | 269.61 | 20.76 | 0.41 | 0.24 | 217.33 | 243.00 |
| 19 | IG 129185 | 35.67 | 277.57 | 36.41 | 0.46 | 0.40 | 273.33 | 309.67 |
| 20 | IG 49 | 41.00 | 228.27 | 36.86 | 0.43 | 0.44 | 614.67 | 442.00 |
| 21 | IG 112078 | 32.00 | 216.54 | 33.20 | 0.48 | 0.46 | 148.67 | 347.00 |
| 22 | IG 73920 | 34.67 | 227.49 | 33.72 | 0.46 | 0.44 | 147.67 | 567.00 |
| 23 | P 3233 | 22.33 | 173.57 | 36.36 | 0.56 | 0.52 | 228.67 | 371.00 |
| 24 | IG 560183 | 29.67 | 325.69 | 22.17 | 0.53 | 0.34 | 134.00 | 387.67 |
| 25 | IPL 406 | 32.67 | 60.68 | 45.49 | 0.48 | 0.49 | 167.00 | 257.33 |
| 26 | IG 560206 | 31.67 | 271.83 | 36.98 | 0.45 | 0.67 | 167.67 | 749.67 |
| 27 | L 4076 | 25.67 | 281.85 | 23.98 | 0.47 | 0.33 | 156.00 | 429.67 |
| 28 | IG 134656 | 31.67 | 252.03 | 22.69 | 0.50 | 0.27 | 147.33 | 214.67 |
| 29 | IG 560185 | 33.67 | 225.04 | 34.63 | 0.50 | 0.46 | 193.00 | 547.33 |
| 30 | L 4698 | 33.00 | 217.00 | 57.58 | 0.65 | 0.76 | 157.33 | 787.00 |
| 31 | PL 97 | 34.33 | 262.22 | 43.25 | 0.46 | 0.30 | 34.33 | 371.00 |
| 32 | P 2208 | 31.67 | 397.71 | 17.51 | 0.56 | 0.25 | 76.67 | 169.67 |
| 33 | ILL 10821 | 29.00 | 167.40 | 25.34 | 0.50 | 0.32 | 165.33 | 371.33 |
| 34 | IG 936 | 31.00 | 194.23 | 12.77 | 0.41 | 0.11 | 132.00 | 215.33 |
| 35 | IPL 321 | 33.00 | 173.10 | 26.45 | 0.49 | 0.27 | 218.67 | 290.67 |
| 36 | P 2116 | 32.00 | 320.51 | 48.05 | 0.45 | 0.56 | 335.33 | 888.33 |
| 37 | IG 334 | 30.33 | 154.99 | 33.89 | 0.46 | 0.26 | 259.33 | 198.00 |
| 38 | IG 560157 | 28.67 | 563.68 | 27.50 | 0.55 | 0.41 | 319.67 | 379.33 |
| 39 | IG 568229 | 24.67 | 199.12 | 17.78 | 0.48 | 0.14 | 109.67 | 72.33 |
| 40 | IG 130033 | 30.00 | 412.93 | 16.79 | 0.46 | 0.30 | 130.33 | 175.00 |
| 41 | IG 112131 | 32.33 | 140.20 | 18.87 | 0.43 | 0.16 | 170.67 | 246.67 |
| 42 | LC 300-19 | 29.00 | 199.26 | 24.62 | 0.45 | 0.22 | 247.33 | 467.00 |
| 43 | Global Mutant | 30.00 | 122.00 | 15.37 | 0.46 | 0.23 | 209.00 | 155.00 |
| 44 | ILL10832 | 40.33 | 122.28 | 27.63 | 0.44 | 0.34 | 126.33 | 268.67 |
| 45 | EC 78518 | 43.00 | 331.74 | 44.33 | 0.45 | 0.47 | 310.33 | 655.33 |
| 46 | IC 560162 | 36.00 | 404.37 | 25.69 | 0.43 | 0.32 | 144.33 | 362.67 |
| 47 | P 2130 | 34.33 | 324.87 | 28.00 | 0.50 | 0.44 | 183.33 | 262.00 |
| 48 | L 4603 | 32.33 | 334.57 | 47.98 | 0.46 | 0.57 | 270.67 | 872.00 |
| 49 | P 8116 | 26.33 | 218.29 | 28.68 | 0.51 | 0.54 | 243.67 | 734.67 |
| 50 | L 4705 | 30.67 | 224.46 | 17.17 | 0.54 | 0.26 | 175.67 | 180.33 |
| 51 | IG 56968 | 29.33 | 212.97 | 44.42 | 0.44 | 0.47 | 260.00 | 804.67 |
| 52 | IG 129302 | 24.00 | 128.92 | 16.44 | 0.45 | 0.15 | 200.67 | 166.67 |
| 53 | P 3226 | 33.00 | 226.43 | 43.92 | 0.52 | 0.15 | 257.33 | 1143.33 |
| 54 | P 560183 | 27.33 | 151.64 | 34.61 | 0.53 | 0.54 | 251.00 | 846.33 |
| 55 | IPL 406-1 | 35.00 | 163.40 | 52.86 | 0.53 | 0.64 | 424.00 | 450.67 |
| 56 | P 560206 | 33.33 | 245.77 | 62.76 | 0.45 | 0.25 | 695.00 | 1176.00 |
| 57 | L 4076-1 | 29.67 | 141.31 | 42.15 | 0.53 | 0.35 | 90.00 | 565.33 |
| 58 | IG 134656 | 30.33 | 254.81 | 46.04 | 0.52 | 0.56 | 117.00 | 429.67 |
| 59 | P 560185 | 31.00 | 160.57 | 23.58 | 0.56 | 0.32 | 179.67 | 233.67 |
| 60 | PL 97-1 | 23.33 | 223.81 | 53.63 | 0.52 | 0.70 | 215.00 | 496.00 |
| 61 | ILWL 147 | 25.67 | 48.36 | 9.43 | 0.42 | 0.10 | 127.00 | 169.33 |
| 62 | IG 129313 | 33.67 | 357.42 | 22.47 | 0.56 | 0.26 | 132.33 | 308.67 |
| 63 | LC 282-1077 | 31.00 | 205.88 | 27.58 | 0.55 | 0.33 | 156.67 | 367.67 |
| 64 | IC 560297 | 29.33 | 175.41 | 34.66 | 0.57 | 0.45 | 236.00 | 458.33 |
| 65 | IC 27986 | 38.33 | 245.47 | 65.85 | 0.53 | 0.87 | 567.33 | 1259.33 |
| 66 | IC 346092 | 39.33 | 277.84 | 43.86 | 0.50 | 0.55 | 541.33 | 789.33 |
| 67 | P 43120 | 42.00 | 361.93 | 75.49 | 0.58 | 1.10 | 514.33 | 961.67 |
| 68 | P 43103 | 31.00 | 184.56 | 36.23 | 0.51 | 0.46 | 350.33 | 511.67 |
| 69 | P 16214 | 27.67 | 149.54 | 25.00 | 0.49 | 0.31 | 346.00 | 325.33 |
| 70 | P 16213 | 28.33 | 105.78 | 26.04 | 0.46 | 0.30 | 280.33 | 231.00 |
| 71 | L 11-280 | 23.00 | 141.19 | 17.12 | 0.48 | 0.18 | 79.33 | 281.67 |
| 72 | L 11-279 | 28.33 | 118.09 | 25.37 | 0.41 | 0.22 | 168.67 | 414.00 |
| 73 | L 11-248 | 26.00 | 100.56 | 14.56 | 0.45 | 0.16 | 93.33 | 245.00 |
| 74 | L 11-244 | 32.00 | 90.16 | 11.68 | 0.43 | 0.12 | 96.67 | 97.33 |
| 75 | L 11-243 | 41.00 | 136.54 | 17.46 | 0.45 | 0.32 | 127.00 | 138.67 |
| 76 | L 11-234 | 30.33 | 46.86 | 6.31 | 0.43 | 0.06 | 98.67 | 52.33 |
| 77 | L 11-231 | 34.00 | 137.75 | 17.40 | 0.43 | 0.19 | 133.33 | 152.67 |
| 78 | PLL-18-1 | 25.67 | 744.34 | 66.76 | 0.44 | 0.78 | 177.67 | 700.33 |
| 79 | PLL 18-2 | 33.67 | 479.06 | 65.96 | 0.41 | 1.71 | 545.67 | 3449.33 |
| 80 | PLL 18-5 | 31.00 | 754.31 | 85.50 | 0.36 | 0.83 | 431.00 | 954.33 |
| 81 | PLL 18-7 | 29.33 | 470.33 | 34.03 | 0.37 | 0.74 | 360.67 | 821.67 |
| 82 | PLL 18-9 | 38.33 | 468.51 | 46.77 | 0.42 | 1.51 | 374.33 | 2941.00 |
| 83 | PLL 18-11 | 39.33 | 472.05 | 47.03 | 0.38 | 0.75 | 550.33 | 1039.33 |
| 84 | PLL 18-12 | 42.00 | 421.05 | 54.81 | 0.35 | 0.57 | 784.00 | 936.67 |
| 85 | PLL 18-14 | 31.00 | 403.52 | 41.36 | 0.35 | 0.88 | 232.00 | 905.67 |
| 86 | PLS-18-21 | 27.67 | 358.87 | 60.68 | 0.38 | 0.52 | 333.00 | 1310.00 |
| 87 | PLS -18-23 | 28.33 | 462.48 | 32.55 | 0.38 | 1.50 | 441.00 | 1253.00 |
| 88 | PLS 18-25 | 23.00 | 734.66 | 40.59 | 0.35 | 0.56 | 536.33 | 934.00 |
| 89 | PLS 18-32 | 28.33 | 751.43 | 115.02 | 0.35 | 1.13 | 520.00 | 1360.33 |
| 90 | PLS 18-33 | 26.00 | 629.72 | 38.64 | 0.37 | 0.45 | 451.67 | 2092.33 |
| 91 | PLS 18-36 | 32.00 | 314.40 | 38.65 | 0.36 | 0.39 | 155.67 | 549.33 |
| 92 | PLS 18-44 | 41.00 | 696.84 | 69.05 | 0.39 | 0.64 | 240.00 | 1026.33 |
| 93 | PLS 18-01 | 30.33 | 632.77 | 40.29 | 0.38 | 1.61 | 629.33 | 3125.33 |
| 94 | PLS 18-02 | 34.00 | 320.50 | 32.69 | 0.42 | 0.34 | 248.33 | 481.67 |
| 95 | PLS 18-03 | 25.67 | 372.51 | 46.87 | 0.38 | 0.55 | 363.33 | 782.67 |
| 96 | PLS 18-04 | 33.67 | 729.28 | 61.83 | 0.38 | 0.63 | 349.67 | 1766.33 |
| 97 | PLS 18-05 | 31.00 | 661.55 | 36.51 | 0.39 | 0.46 | 448.33 | 812.33 |
| 98 | PLS 18-57 | 25.67 | 175.18 | 23.98 | 0.47 | 0.33 | 156.00 | 429.67 |
| 99 | PLS 18-58 | 31.67 | 125.36 | 22.69 | 0.50 | 0.27 | 147.33 | 214.67 |
| 100 | PLS 18-60 | 33.67 | 105.04 | 24.63 | 0.50 | 0.46 | 193.00 | 547.33 |
| 101 | PLS 18-62 | 33.00 | 177.00 | 27.58 | 0.65 | 0.76 | 157.33 | 787.00 |
| 102 | PLS 18-64 | 34.33 | 118.89 | 13.25 | 0.46 | 0.30 | 34.33 | 371.00 |
| 103 | PLS 18-65 | 31.67 | 224.37 | 11.18 | 0.56 | 0.25 | 76.67 | 169.67 |
| 104 | PLS 18-66 | 29.00 | 157.40 | 25.34 | 0.50 | 0.32 | 165.33 | 371.33 |
| 105 | PLS 18-67 | 31.00 | 164.23 | 12.77 | 0.41 | 0.11 | 132.00 | 215.33 |
| 106 | PLL 18-22 | 33.00 | 156.43 | 26.45 | 0.49 | 0.27 | 218.67 | 290.67 |
| 107 | PLL 18-23 | 32.00 | 320.51 | 48.05 | 0.45 | 0.56 | 335.33 | 888.33 |
| 108 | PLL 18-24 | 30.33 | 154.99 | 33.89 | 0.46 | 0.26 | 259.33 | 198.00 |
| 109 | PLL 18-25 | 28.67 | 363.68 | 27.50 | 0.55 | 0.41 | 319.67 | 379.33 |
| 110 | PLL 18-26 | 24.67 | 399.12 | 17.78 | 0.48 | 0.14 | 109.67 | 72.33 |

Raw data of root diameter classes (Total Root Length and Total Surface Area) under P + conditions:

|  | Total Root Length | | | | | Total Surface Area | | | | |
| --- | --- | --- | --- | --- | --- | --- | --- | --- | --- | --- |
| Genotypes | TRL1 (0-0.5mm) | TRL2 (0.5-1.0 mm) | TRL3 (1.0-1.5mm) | TRL4 (1.5-2.0mm) | TRL5 (>2mm) | TSA1 (0-0.5mm) | TSA2 (0.5-1.0 mm) | TSA3 (1.0-1.5mm) | TSA4 (1.5-2.0mm) | TSA5 (>2mm) |
| 1 | 594.24 | 153.59 | 14.17 | 6.53 | 2.08 | 52.17 | 30.29 | 5.28 | 3.49 | 1.44 |
| 2 | 466.56 | 73.64 | 9.63 | 1.77 | 1.10 | 46.17 | 14.43 | 3.70 | 0.94 | 0.79 |
| 3 | 425.11 | 108.40 | 8.52 | 3.84 | 1.69 | 39.59 | 20.58 | 3.26 | 2.04 | 1.18 |
| 4 | 571.20 | 128.40 | 13.05 | 5.75 | 1.22 | 53.40 | 25.03 | 4.93 | 3.08 | 0.85 |
| 5 | 308.47 | 59.78 | 9.67 | 4.08 | 1.67 | 28.91 | 12.31 | 3.67 | 2.16 | 1.16 |
| 6 | 348.67 | 48.50 | 8.48 | 2.00 | 0.48 | 33.66 | 9.58 | 3.17 | 1.05 | 0.33 |
| 7 | 443.63 | 111.84 | 12.97 | 5.57 | 4.00 | 40.70 | 21.93 | 4.83 | 3.07 | 2.81 |
| 8 | 312.63 | 50.26 | 9.53 | 4.08 | 1.66 | 31.10 | 10.40 | 3.70 | 2.16 | 1.15 |
| 9 | 392.96 | 106.95 | 11.20 | 5.32 | 2.95 | 36.56 | 21.11 | 4.24 | 2.89 | 2.06 |
| 10 | 333.61 | 56.72 | 5.88 | 2.40 | 0.46 | 30.64 | 11.40 | 2.26 | 1.27 | 0.32 |
| 11 | 165.63 | 26.40 | 1.74 | 0.18 | 0.10 | 15.54 | 5.20 | 0.63 | 0.11 | 0.07 |
| 12 | 116.40 | 12.27 | 0.94 | 0.35 | 0.34 | 10.85 | 2.50 | 0.35 | 0.19 | 0.24 |
| 13 | 346.36 | 70.58 | 9.89 | 5.09 | 1.14 | 31.23 | 14.05 | 3.70 | 2.76 | 0.82 |
| 14 | 326.51 | 81.96 | 10.75 | 3.32 | 1.17 | 28.43 | 16.16 | 4.02 | 1.78 | 0.82 |
| 15 | 251.61 | 36.72 | 7.60 | 2.22 | 0.47 | 23.00 | 7.49 | 2.90 | 1.16 | 0.32 |
| 16 | 168.18 | 46.59 | 5.58 | 1.80 | 0.54 | 17.61 | 9.04 | 2.12 | 0.95 | 0.36 |
| 17 | 372.03 | 101.40 | 9.40 | 4.34 | 1.56 | 35.19 | 19.63 | 3.61 | 2.32 | 1.10 |
| 18 | 276.69 | 43.64 | 4.94 | 2.11 | 1.26 | 24.61 | 8.77 | 1.92 | 1.16 | 0.89 |
| 19 | 313.25 | 57.88 | 8.37 | 4.92 | 1.31 | 27.98 | 11.58 | 3.20 | 2.63 | 0.91 |
| 20 | 205.00 | 47.97 | 6.08 | 2.01 | 0.22 | 18.58 | 9.37 | 2.32 | 1.03 | 0.15 |
| 21 | 233.83 | 57.41 | 5.11 | 1.05 | 0.33 | 21.64 | 11.24 | 1.90 | 0.57 | 0.23 |
| 22 | 307.95 | 62.77 | 7.18 | 2.29 | 1.26 | 28.84 | 12.29 | 2.73 | 1.24 | 0.90 |
| 23 | 255.80 | 76.49 | 10.58 | 5.12 | 1.82 | 23.61 | 15.29 | 4.06 | 2.74 | 1.26 |
| 24 | 432.85 | 88.08 | 9.57 | 5.16 | 1.14 | 42.13 | 17.46 | 3.61 | 2.77 | 0.77 |
| 25 | 147.77 | 63.14 | 8.09 | 3.64 | 0.68 | 14.19 | 12.17 | 3.07 | 1.91 | 0.47 |
| 26 | 278.44 | 55.97 | 6.18 | 3.21 | 0.89 | 25.61 | 11.13 | 2.36 | 1.71 | 0.62 |
| 27 | 262.12 | 75.36 | 8.92 | 3.01 | 1.00 | 23.45 | 15.15 | 3.36 | 1.61 | 0.70 |
| 28 | 321.94 | 76.33 | 7.16 | 5.84 | 1.41 | 30.26 | 15.12 | 2.66 | 3.14 | 1.01 |
| 29 | 191.53 | 46.11 | 8.18 | 1.24 | 0.54 | 17.45 | 9.21 | 3.11 | 0.64 | 0.39 |
| 30 | 292.62 | 77.32 | 11.52 | 5.30 | 1.98 | 26.00 | 15.25 | 4.31 | 2.83 | 1.37 |
| 31 | 311.42 | 57.26 | 10.35 | 1.23 | 1.52 | 28.98 | 11.46 | 3.86 | 0.66 | 1.05 |
| 32 | 240.22 | 55.11 | 7.96 | 2.36 | 0.96 | 23.30 | 10.92 | 3.07 | 1.28 | 0.67 |
| 33 | 287.15 | 85.33 | 11.78 | 4.53 | 1.25 | 28.20 | 16.86 | 4.45 | 2.43 | 0.86 |
| 34 | 228.79 | 47.28 | 7.07 | 2.27 | 0.35 | 20.27 | 9.66 | 2.63 | 1.22 | 0.24 |
| 35 | 306.82 | 68.00 | 11.77 | 4.44 | 1.79 | 29.51 | 13.98 | 4.45 | 2.41 | 1.32 |
| 36 | 219.00 | 79.45 | 9.13 | 4.35 | 1.51 | 20.77 | 15.42 | 3.42 | 2.36 | 1.09 |
| 37 | 267.57 | 89.97 | 14.65 | 7.32 | 3.15 | 25.51 | 17.95 | 5.56 | 3.94 | 2.20 |
| 38 | 392.48 | 107.65 | 16.49 | 4.99 | 1.95 | 38.00 | 22.11 | 6.06 | 2.66 | 1.36 |
| 39 | 267.92 | 62.29 | 10.86 | 4.07 | 1.04 | 24.30 | 12.95 | 4.15 | 2.17 | 0.74 |
| 40 | 366.24 | 97.85 | 16.22 | 6.97 | 2.07 | 33.35 | 19.77 | 6.09 | 3.70 | 1.45 |
| 41 | 229.54 | 92.16 | 13.04 | 4.45 | 2.54 | 19.77 | 18.42 | 4.93 | 2.41 | 1.77 |
| 42 | 292.89 | 80.32 | 9.99 | 4.70 | 2.10 | 27.66 | 16.07 | 3.80 | 2.51 | 1.46 |
| 43 | 178.39 | 36.95 | 5.12 | 1.27 | 0.58 | 16.52 | 7.60 | 1.91 | 0.68 | 0.40 |
| 44 | 216.55 | 49.62 | 11.32 | 4.91 | 2.24 | 19.51 | 10.29 | 4.21 | 2.61 | 1.60 |
| 45 | 224.26 | 76.08 | 13.81 | 5.49 | 2.28 | 20.83 | 15.68 | 5.22 | 2.98 | 1.60 |
| 46 | 236.08 | 66.77 | 16.14 | 5.15 | 0.85 | 20.86 | 13.82 | 6.07 | 2.71 | 0.60 |
| 47 | 194.84 | 52.00 | 5.99 | 3.01 | 1.10 | 18.28 | 10.34 | 2.28 | 1.63 | 0.81 |
| 48 | 275.42 | 75.84 | 19.34 | 9.23 | 3.20 | 24.56 | 15.94 | 7.37 | 5.00 | 2.26 |
| 49 | 153.27 | 53.11 | 9.66 | 4.30 | 2.36 | 13.06 | 10.85 | 3.67 | 2.30 | 1.66 |
| 50 | 189.75 | 75.11 | 13.47 | 7.67 | 2.61 | 16.74 | 15.77 | 5.13 | 4.18 | 1.83 |
| 51 | 130.90 | 47.19 | 9.98 | 5.30 | 1.65 | 11.58 | 9.72 | 3.76 | 2.85 | 1.15 |
| 52 | 167.35 | 48.83 | 9.88 | 5.05 | 2.92 | 14.94 | 10.14 | 3.70 | 2.76 | 2.06 |
| 53 | 194.81 | 71.34 | 12.41 | 7.38 | 4.21 | 16.33 | 14.59 | 4.78 | 3.98 | 3.00 |
| 54 | 161.03 | 54.30 | 12.47 | 5.68 | 2.60 | 14.16 | 11.04 | 4.73 | 3.07 | 1.83 |
| 55 | 90.81 | 36.24 | 9.01 | 3.68 | 2.51 | 8.00 | 7.61 | 3.48 | 1.97 | 1.77 |
| 56 | 172.31 | 59.69 | 11.81 | 6.25 | 2.46 | 15.82 | 12.21 | 4.52 | 3.37 | 1.74 |
| 57 | 153.52 | 57.60 | 13.28 | 6.47 | 4.08 | 13.97 | 11.77 | 5.10 | 3.53 | 2.85 |
| 58 | 273.03 | 93.67 | 21.51 | 11.55 | 5.62 | 24.80 | 19.28 | 8.11 | 6.28 | 3.96 |
| 59 | 149.12 | 38.64 | 7.84 | 4.39 | 1.18 | 13.15 | 7.82 | 3.02 | 2.37 | 0.80 |
| 60 | 216.42 | 78.67 | 12.55 | 6.30 | 4.77 | 18.31 | 15.99 | 4.81 | 3.42 | 3.39 |
| 61 | 49.14 | 9.77 | 1.96 | 0.56 | 0.17 | 4.24 | 2.11 | 0.73 | 0.30 | 0.12 |
| 62 | 222.24 | 70.73 | 12.94 | 5.91 | 3.21 | 21.09 | 14.51 | 4.89 | 3.20 | 2.23 |
| 63 | 234.13 | 72.11 | 13.12 | 5.65 | 2.63 | 22.20 | 14.29 | 4.96 | 3.06 | 1.83 |
| 64 | 307.37 | 105.51 | 21.31 | 8.56 | 4.67 | 27.98 | 21.72 | 8.02 | 4.61 | 3.26 |
| 65 | 139.07 | 31.60 | 6.23 | 2.88 | 0.67 | 12.26 | 6.63 | 2.36 | 1.53 | 0.47 |
| 66 | 182.91 | 67.45 | 15.71 | 6.53 | 3.29 | 16.24 | 14.00 | 5.96 | 3.54 | 2.28 |
| 67 | 125.47 | 38.19 | 10.03 | 5.41 | 2.89 | 11.57 | 7.92 | 3.81 | 2.94 | 2.06 |
| 68 | 193.50 | 75.74 | 11.03 | 5.36 | 1.72 | 18.45 | 15.31 | 4.14 | 2.87 | 1.23 |
| 69 | 171.62 | 68.45 | 14.17 | 6.88 | 2.38 | 15.59 | 14.01 | 5.33 | 3.71 | 1.64 |
| 70 | 142.46 | 43.31 | 8.61 | 3.34 | 1.08 | 13.28 | 8.88 | 3.29 | 1.78 | 0.76 |
| 71 | 213.04 | 94.89 | 16.66 | 9.84 | 3.63 | 18.67 | 19.43 | 6.35 | 5.34 | 2.55 |
| 72 | 83.59 | 29.91 | 3.37 | 0.98 | 0.41 | 8.26 | 6.03 | 1.26 | 0.52 | 0.29 |
| 73 | 120.99 | 31.57 | 3.49 | 0.26 | 0.05 | 11.13 | 6.31 | 1.31 | 0.14 | 0.03 |
| 74 | 171.80 | 48.41 | 10.29 | 3.90 | 1.75 | 15.88 | 10.16 | 3.81 | 2.09 | 1.26 |
| 75 | 125.84 | 39.51 | 5.80 | 2.12 | 1.05 | 11.98 | 8.03 | 2.19 | 1.11 | 0.73 |
| 76 | 129.08 | 34.56 | 5.44 | 2.33 | 0.90 | 11.52 | 6.96 | 2.05 | 1.23 | 0.63 |
| 77 | 104.07 | 29.01 | 5.33 | 1.64 | 0.82 | 9.76 | 6.10 | 2.01 | 0.89 | 0.58 |
| 78 | 250.46 | 74.07 | 12.28 | 4.78 | 2.24 | 22.21 | 15.12 | 4.63 | 2.55 | 1.57 |
| 79 | 123.63 | 35.81 | 8.21 | 3.67 | 1.75 | 11.26 | 7.48 | 3.12 | 1.98 | 1.22 |
| 80 | 185.48 | 75.15 | 11.26 | 5.05 | 1.89 | 17.37 | 15.13 | 4.23 | 2.73 | 1.35 |
| 81 | 169.69 | 65.41 | 14.55 | 6.80 | 2.30 | 15.54 | 13.48 | 5.51 | 3.66 | 1.60 |
| 82 | 149.04 | 57.86 | 7.89 | 3.58 | 1.14 | 13.42 | 11.78 | 2.97 | 1.92 | 0.80 |
| 83 | 215.66 | 82.17 | 16.58 | 9.62 | 3.38 | 19.61 | 16.89 | 6.33 | 5.20 | 2.36 |
| 84 | 60.55 | 23.63 | 2.28 | 0.18 | 0.37 | 5.95 | 4.81 | 0.87 | 0.10 | 0.25 |
| 85 | 229.17 | 60.34 | 11.91 | 3.78 | 1.61 | 21.09 | 12.45 | 4.43 | 2.02 | 1.17 |
| 86 | 69.39 | 20.67 | 1.56 | 0.39 | 0.25 | 6.37 | 4.11 | 0.56 | 0.22 | 0.17 |
| 87 | 136.59 | 43.81 | 7.47 | 2.62 | 1.48 | 12.82 | 8.93 | 2.85 | 1.38 | 1.04 |
| 88 | 121.77 | 29.13 | 5.04 | 1.92 | 0.43 | 10.83 | 5.91 | 1.90 | 1.02 | 0.29 |
| 89 | 285.54 | 55.68 | 7.86 | 2.62 | 1.28 | 26.39 | 11.29 | 3.00 | 1.40 | 0.90 |
| 90 | 509.23 | 129.44 | 14.28 | 6.60 | 1.59 | 48.22 | 25.45 | 5.37 | 3.52 | 1.11 |
| 91 | 289.58 | 45.36 | 6.68 | 3.20 | 1.06 | 27.77 | 9.28 | 2.52 | 1.70 | 0.73 |
| 92 | 347.00 | 61.59 | 9.29 | 2.60 | 1.34 | 32.58 | 12.08 | 3.45 | 1.40 | 0.94 |
| 93 | 447.26 | 96.10 | 12.83 | 4.63 | 3.24 | 42.10 | 18.98 | 4.82 | 2.53 | 2.27 |
| 94 | 148.94 | 41.86 | 7.68 | 2.70 | 1.40 | 13.79 | 8.57 | 2.89 | 1.47 | 0.97 |
| 95 | 196.56 | 74.33 | 14.90 | 9.58 | 5.81 | 15.59 | 15.52 | 5.66 | 5.20 | 4.14 |
| 96 | 176.03 | 61.30 | 12.38 | 5.00 | 2.21 | 15.96 | 12.37 | 4.72 | 2.70 | 1.55 |
| 97 | 54.76 | 16.43 | 4.78 | 2.96 | 1.80 | 4.54 | 3.35 | 1.86 | 1.60 | 1.28 |
| 98 | 209.48 | 66.07 | 11.58 | 4.45 | 2.15 | 20.02 | 13.35 | 4.40 | 2.35 | 1.52 |
| 99 | 338.20 | 73.48 | 11.18 | 3.48 | 1.31 | 28.73 | 14.80 | 4.20 | 1.85 | 0.91 |
| 100 | 190.72 | 31.11 | 5.68 | 1.23 | 0.14 | 18.19 | 6.11 | 2.17 | 0.65 | 0.10 |
| 101 | 201.53 | 64.73 | 6.93 | 2.81 | 0.98 | 20.16 | 12.67 | 2.66 | 1.46 | 0.66 |
| 102 | 344.60 | 83.95 | 7.44 | 3.58 | 1.38 | 32.05 | 16.19 | 2.85 | 1.94 | 0.99 |
| 103 | 328.60 | 55.70 | 7.74 | 4.50 | 1.49 | 29.25 | 11.37 | 2.98 | 2.43 | 1.05 |
| 104 | 127.36 | 22.42 | 4.54 | 3.00 | 1.72 | 11.12 | 4.42 | 1.73 | 1.61 | 1.21 |
| 105 | 114.36 | 45.67 | 9.68 | 4.19 | 2.40 | 10.19 | 9.52 | 3.75 | 2.22 | 1.72 |
| 106 | 176.15 | 53.85 | 11.15 | 5.70 | 1.95 | 16.66 | 11.04 | 4.28 | 3.09 | 1.37 |
| 107 | 187.42 | 67.87 | 14.88 | 8.24 | 5.24 | 16.48 | 13.94 | 5.69 | 4.48 | 3.69 |
| 108 | 237.06 | 79.95 | 18.75 | 9.04 | 4.26 | 21.86 | 16.39 | 7.08 | 4.93 | 2.99 |
| 109 | 204.63 | 37.07 | 4.58 | 2.97 | 0.58 | 18.57 | 7.58 | 1.72 | 1.62 | 0.40 |
| 110 | 335.29 | 68.11 | 9.54 | 3.68 | 1.14 | 31.25 | 13.24 | 3.56 | 1.98 | 0.81 |

Raw data of root diameter classes (Total Root Length and Total Surface Area) under P - conditions:

|  | TRL | | | | | TSA | | | | |
| --- | --- | --- | --- | --- | --- | --- | --- | --- | --- | --- |
| Genotypes | L1 (0-0.5mm) | TRL2 (0.5-1.0 mm) | TRL3 (1.0-1.5mm) | TRL4 (1.5-2.0mm) | TRL5 (>2mm) | TSA1 (0-0.5mm) | TSA2 (0.5-1.0 mm) | TSA3 (1.0-1.5mm) | TSA4 (1.5-2.0mm) | TSA5 (>2mm) |
| 1 | 281.23 | 49.17 | 8.54 | 1.83 | 0.53 | 26.25 | 10.28 | 3.18 | 0.99 | 0.36 |
| 2 | 346.15 | 64.93 | 6.95 | 1.45 | 0.45 | 34.23 | 13.09 | 2.63 | 0.75 | 0.32 |
| 3 | 224.96 | 32.37 | 4.69 | 1.65 | 0.34 | 21.28 | 6.85 | 1.79 | 0.87 | 0.23 |
| 4 | 294.42 | 59.45 | 6.66 | 2.40 | 0.48 | 28.77 | 12.22 | 2.55 | 1.26 | 0.34 |
| 5 | 417.58 | 110.82 | 14.37 | 6.02 | 1.48 | 38.62 | 22.05 | 5.41 | 3.17 | 1.04 |
| 6 | 315.59 | 51.25 | 8.85 | 2.47 | 1.15 | 30.35 | 10.46 | 3.29 | 1.30 | 0.80 |
| 7 | 440.96 | 79.86 | 12.35 | 4.60 | 1.67 | 41.88 | 16.15 | 4.68 | 2.46 | 1.19 |
| 8 | 175.17 | 34.83 | 4.67 | 1.62 | 0.69 | 15.97 | 7.10 | 1.78 | 0.85 | 0.47 |
| 9 | 384.60 | 48.56 | 8.64 | 2.91 | 0.50 | 33.91 | 9.86 | 3.25 | 1.59 | 0.36 |
| 10 | 223.48 | 39.39 | 4.71 | 2.63 | 1.09 | 20.61 | 7.93 | 1.78 | 1.41 | 0.78 |
| 11 | 429.21 | 95.14 | 12.39 | 1.81 | 0.40 | 38.41 | 19.03 | 4.64 | 0.97 | 0.28 |
| 12 | 126.95 | 25.81 | 4.11 | 2.42 | 1.05 | 11.96 | 5.20 | 1.55 | 1.30 | 0.74 |
| 13 | 212.41 | 26.22 | 3.73 | 1.37 | 0.62 | 18.84 | 5.17 | 1.36 | 0.72 | 0.43 |
| 14 | 217.69 | 28.79 | 3.81 | 1.50 | 0.56 | 19.78 | 5.55 | 1.47 | 0.79 | 0.40 |
| 15 | 267.60 | 36.00 | 4.28 | 1.03 | 0.51 | 24.18 | 7.27 | 1.57 | 0.58 | 0.36 |
| 16 | 166.60 | 45.28 | 5.38 | 2.06 | 1.49 | 16.63 | 8.62 | 1.98 | 1.15 | 1.04 |
| 17 | 250.96 | 88.28 | 8.11 | 2.63 | 1.72 | 22.64 | 16.91 | 3.02 | 1.41 | 1.21 |
| 18 | 158.24 | 16.23 | 2.87 | 0.83 | 0.80 | 15.08 | 3.27 | 1.10 | 0.44 | 0.56 |
| 19 | 197.25 | 50.85 | 3.30 | 1.81 | 0.69 | 17.76 | 9.39 | 1.24 | 0.99 | 0.50 |
| 20 | 266.63 | 74.50 | 6.01 | 3.41 | 1.60 | 21.78 | 13.80 | 2.30 | 1.84 | 1.13 |
| 21 | 276.39 | 73.64 | 11.52 | 4.56 | 1.29 | 25.57 | 14.92 | 4.35 | 2.45 | 0.92 |
| 22 | 246.61 | 68.75 | 7.04 | 2.51 | 1.26 | 22.66 | 13.61 | 2.60 | 1.33 | 0.88 |
| 23 | 252.67 | 91.01 | 14.71 | 6.28 | 4.05 | 21.15 | 18.67 | 5.51 | 3.40 | 2.85 |
| 24 | 248.05 | 83.65 | 15.02 | 6.77 | 2.67 | 22.64 | 17.27 | 5.71 | 3.63 | 1.90 |
| 25 | 219.40 | 58.68 | 9.89 | 4.81 | 2.54 | 18.67 | 12.07 | 3.71 | 2.57 | 1.80 |
| 26 | 243.89 | 77.45 | 10.14 | 4.29 | 1.90 | 21.93 | 15.27 | 3.86 | 2.33 | 1.34 |
| 27 | 227.52 | 70.51 | 10.52 | 4.00 | 1.54 | 19.53 | 14.10 | 4.06 | 2.13 | 1.08 |
| 28 | 131.56 | 42.71 | 7.16 | 2.88 | 0.67 | 12.85 | 8.62 | 2.70 | 1.52 | 0.47 |
| 29 | 165.30 | 54.86 | 9.11 | 3.20 | 1.31 | 15.86 | 10.87 | 3.46 | 1.72 | 0.94 |
| 30 | 198.25 | 108.43 | 20.17 | 8.56 | 4.27 | 17.43 | 21.79 | 7.64 | 4.65 | 2.98 |
| 31 | 143.74 | 41.54 | 6.21 | 2.07 | 1.05 | 13.93 | 8.17 | 2.38 | 1.14 | 0.72 |
| 32 | 148.57 | 62.97 | 9.09 | 4.66 | 2.36 | 12.50 | 12.36 | 3.44 | 2.56 | 1.62 |
| 33 | 127.83 | 46.21 | 5.51 | 2.66 | 0.81 | 11.48 | 9.07 | 2.07 | 1.42 | 0.56 |
| 34 | 152.96 | 34.15 | 4.05 | 1.60 | 0.56 | 12.83 | 6.82 | 1.49 | 0.89 | 0.40 |
| 35 | 164.37 | 37.90 | 7.86 | 3.57 | 0.91 | 14.60 | 7.87 | 2.94 | 1.93 | 0.63 |
| 36 | 245.94 | 74.23 | 10.09 | 3.77 | 0.82 | 22.92 | 14.56 | 3.78 | 2.00 | 0.57 |
| 37 | 223.36 | 52.49 | 7.90 | 2.87 | 1.06 | 20.16 | 10.46 | 2.97 | 1.52 | 0.71 |
| 38 | 217.47 | 61.79 | 10.18 | 3.31 | 1.26 | 20.21 | 12.53 | 3.84 | 1.79 | 0.89 |
| 39 | 208.85 | 45.87 | 8.63 | 3.37 | 1.04 | 19.56 | 9.31 | 3.23 | 1.79 | 0.72 |
| 40 | 96.47 | 29.06 | 4.13 | 1.99 | 0.38 | 8.84 | 5.60 | 1.58 | 1.07 | 0.27 |
| 41 | 141.41 | 26.47 | 3.72 | 1.06 | 0.62 | 13.64 | 5.18 | 1.40 | 0.58 | 0.42 |
| 42 | 193.44 | 40.34 | 7.11 | 3.16 | 0.92 | 16.90 | 8.06 | 2.80 | 1.74 | 0.65 |
| 43 | 114.87 | 23.06 | 3.42 | 0.61 | 0.01 | 10.88 | 4.61 | 1.25 | 0.31 | 0.01 |
| 44 | 165.83 | 46.72 | 7.22 | 1.54 | 0.45 | 15.32 | 9.15 | 2.77 | 0.83 | 0.33 |
| 45 | 255.18 | 48.58 | 7.76 | 2.41 | 0.65 | 25.05 | 9.76 | 2.96 | 1.29 | 0.44 |
| 46 | 187.73 | 35.20 | 8.97 | 1.58 | 0.58 | 16.90 | 7.15 | 3.34 | 0.86 | 0.40 |
| 47 | 170.23 | 56.61 | 10.76 | 2.64 | 0.74 | 16.34 | 11.49 | 4.02 | 1.39 | 0.52 |
| 48 | 256.48 | 78.44 | 9.10 | 2.93 | 1.52 | 24.52 | 15.58 | 3.40 | 1.55 | 1.10 |
| 49 | 207.89 | 62.98 | 9.94 | 4.17 | 1.55 | 18.73 | 12.96 | 3.79 | 2.26 | 1.08 |
| 50 | 126.69 | 49.77 | 8.44 | 3.89 | 1.15 | 11.39 | 10.29 | 3.19 | 2.09 | 0.78 |
| 51 | 169.97 | 48.25 | 9.30 | 3.39 | 1.39 | 14.25 | 9.78 | 3.57 | 1.85 | 0.96 |
| 52 | 67.16 | 24.94 | 2.55 | 0.37 | 0.32 | 6.15 | 4.91 | 0.96 | 0.21 | 0.23 |
| 53 | 252.85 | 92.01 | 13.92 | 6.55 | 2.51 | 23.70 | 18.79 | 5.26 | 3.55 | 1.73 |
| 54 | 179.57 | 48.88 | 7.62 | 4.24 | 1.74 | 16.84 | 9.97 | 2.88 | 2.33 | 1.21 |
| 55 | 261.88 | 79.09 | 15.90 | 7.15 | 2.77 | 24.08 | 15.92 | 6.12 | 3.84 | 1.96 |
| 56 | 274.08 | 82.60 | 11.86 | 5.00 | 1.57 | 24.13 | 16.74 | 4.52 | 2.65 | 1.10 |
| 57 | 135.48 | 41.02 | 7.28 | 1.93 | 0.42 | 13.61 | 8.14 | 2.71 | 1.02 | 0.29 |
| 58 | 255.66 | 77.69 | 12.43 | 5.35 | 2.06 | 26.83 | 15.75 | 4.67 | 2.90 | 1.42 |
| 59 | 179.10 | 64.73 | 9.84 | 4.04 | 1.00 | 17.84 | 13.00 | 3.75 | 2.19 | 0.69 |
| 60 | 160.81 | 65.58 | 8.36 | 4.87 | 2.29 | 13.82 | 12.92 | 3.15 | 2.64 | 1.61 |
| 61 | 62.79 | 6.48 | 1.47 | 0.62 | 0.75 | 5.51 | 1.35 | 0.54 | 0.34 | 0.52 |
| 62 | 199.18 | 64.87 | 13.55 | 6.60 | 2.83 | 18.70 | 13.26 | 5.12 | 3.55 | 2.00 |
| 63 | 164.26 | 47.96 | 9.94 | 3.66 | 1.73 | 14.54 | 9.74 | 3.76 | 1.98 | 1.23 |
| 64 | 194.83 | 64.54 | 11.66 | 5.26 | 2.83 | 18.04 | 12.90 | 4.44 | 2.84 | 2.00 |
| 65 | 275.33 | 100.55 | 16.14 | 6.90 | 3.55 | 24.07 | 19.71 | 6.08 | 3.71 | 2.48 |
| 66 | 207.79 | 50.61 | 12.39 | 4.45 | 1.27 | 19.40 | 10.41 | 4.74 | 2.34 | 0.86 |
| 67 | 225.05 | 150.14 | 20.47 | 8.45 | 3.10 | 19.93 | 29.09 | 7.75 | 4.67 | 2.17 |
| 68 | 149.37 | 61.58 | 9.46 | 3.64 | 1.80 | 13.65 | 12.17 | 3.51 | 1.89 | 1.24 |
| 69 | 125.74 | 26.68 | 4.58 | 2.06 | 1.05 | 10.88 | 5.64 | 1.74 | 1.10 | 0.71 |
| 70 | 138.81 | 30.07 | 7.06 | 1.91 | 0.66 | 12.25 | 6.23 | 2.68 | 1.01 | 0.46 |
| 71 | 98.38 | 25.55 | 2.92 | 0.52 | 0.25 | 8.65 | 5.27 | 1.08 | 0.28 | 0.17 |
| 72 | 123.49 | 25.03 | 4.39 | 0.82 | 0.12 | 10.44 | 5.24 | 1.63 | 0.44 | 0.09 |
| 73 | 85.38 | 23.38 | 3.36 | 1.38 | 0.16 | 7.43 | 4.71 | 1.29 | 0.75 | 0.10 |
| 74 | 61.43 | 19.28 | 2.28 | 0.32 | 0.12 | 5.30 | 4.00 | 0.85 | 0.17 | 0.08 |
| 75 | 96.28 | 30.64 | 5.22 | 0.66 | 0.22 | 8.21 | 6.59 | 1.89 | 0.35 | 0.16 |
| 76 | 37.83 | 12.98 | 1.21 | 0.02 | 0.00 | 3.26 | 2.73 | 0.45 | 0.01 | 0.00 |
| 77 | 96.27 | 18.57 | 1.29 | 0.53 | 0.50 | 8.89 | 3.88 | 0.48 | 0.28 | 0.36 |
| 78 | 294.48 | 55.52 | 5.72 | 1.48 | 0.53 | 29.04 | 11.39 | 2.14 | 0.77 | 0.38 |
| 79 | 326.51 | 47.84 | 6.48 | 1.86 | 0.36 | 31.70 | 9.77 | 2.50 | 0.98 | 0.23 |
| 80 | 249.34 | 50.98 | 5.43 | 1.91 | 0.20 | 23.88 | 10.57 | 2.04 | 1.00 | 0.13 |
| 81 | 268.62 | 78.96 | 10.05 | 3.89 | 1.44 | 24.61 | 15.65 | 3.81 | 2.06 | 1.01 |
| 82 | 333.25 | 69.88 | 9.42 | 3.21 | 0.58 | 31.65 | 14.23 | 3.53 | 1.67 | 0.41 |
| 83 | 433.50 | 65.55 | 13.27 | 5.07 | 2.38 | 42.11 | 13.50 | 4.99 | 2.72 | 1.68 |
| 84 | 315.80 | 60.58 | 7.42 | 2.06 | 0.43 | 29.50 | 12.14 | 2.83 | 1.08 | 0.30 |
| 85 | 182.88 | 31.18 | 6.20 | 2.41 | 0.80 | 15.65 | 6.35 | 2.36 | 1.28 | 0.55 |
| 86 | 460.60 | 67.90 | 8.53 | 4.12 | 1.17 | 42.32 | 13.63 | 3.19 | 2.24 | 0.84 |
| 87 | 288.79 | 63.13 | 7.94 | 1.06 | 0.15 | 25.78 | 12.46 | 2.99 | 0.58 | 0.11 |
| 88 | 280.08 | 59.91 | 8.47 | 2.65 | 1.22 | 25.46 | 12.21 | 3.17 | 1.42 | 0.85 |
| 89 | 150.82 | 24.13 | 2.10 | 1.17 | 0.38 | 13.64 | 4.70 | 0.78 | 0.63 | 0.27 |
| 90 | 219.32 | 23.53 | 4.94 | 1.51 | 0.44 | 19.51 | 4.62 | 1.84 | 0.79 | 0.31 |
| 91 | 290.66 | 37.97 | 4.79 | 1.68 | 1.03 | 26.10 | 7.51 | 1.81 | 0.91 | 0.73 |
| 92 | 159.83 | 35.80 | 3.76 | 1.29 | 1.12 | 15.42 | 6.98 | 1.37 | 0.73 | 0.78 |
| 93 | 202.73 | 73.41 | 8.06 | 2.84 | 1.65 | 18.77 | 14.00 | 2.99 | 1.54 | 1.15 |
| 94 | 215.97 | 43.89 | 4.19 | 1.17 | 0.98 | 20.23 | 8.55 | 1.57 | 0.64 | 0.67 |
| 95 | 147.56 | 25.97 | 3.44 | 1.90 | 0.87 | 14.14 | 5.00 | 1.32 | 1.04 | 0.63 |
| 96 | 138.81 | 30.07 | 7.06 | 1.91 | 0.66 | 12.25 | 6.23 | 2.68 | 1.01 | 0.46 |
| 97 | 98.38 | 25.55 | 2.92 | 0.52 | 0.25 | 8.65 | 5.27 | 1.08 | 0.28 | 0.17 |
| 98 | 123.49 | 25.03 | 4.39 | 0.82 | 0.12 | 10.44 | 5.24 | 1.63 | 0.44 | 0.09 |
| 99 | 85.38 | 23.38 | 3.36 | 1.38 | 0.16 | 7.43 | 4.71 | 1.29 | 0.75 | 0.10 |
| 100 | 61.43 | 19.28 | 2.28 | 0.32 | 0.12 | 5.30 | 4.00 | 0.85 | 0.17 | 0.08 |
| 101 | 96.28 | 30.64 | 5.22 | 0.66 | 0.22 | 8.21 | 6.59 | 1.89 | 0.35 | 0.16 |
| 102 | 37.83 | 12.98 | 1.21 | 0.02 | 0.00 | 3.26 | 2.73 | 0.45 | 0.01 | 0.00 |
| 103 | 96.27 | 18.57 | 1.29 | 0.53 | 0.50 | 8.89 | 3.88 | 0.48 | 0.28 | 0.36 |
| 104 | 152.68 | 39.08 | 7.31 | 1.81 | 0.26 | 14.94 | 8.10 | 2.73 | 0.95 | 0.18 |
| 105 | 260.76 | 87.38 | 11.28 | 3.30 | 1.79 | 24.75 | 17.48 | 4.23 | 1.76 | 1.28 |
| 106 | 179.08 | 54.97 | 8.43 | 3.08 | 0.87 | 16.69 | 11.19 | 3.20 | 1.67 | 0.62 |
| 107 | 210.81 | 70.20 | 10.78 | 4.59 | 1.77 | 18.65 | 14.41 | 4.09 | 2.47 | 1.22 |
| 108 | 126.04 | 36.10 | 6.89 | 3.14 | 1.06 | 11.63 | 7.41 | 2.63 | 1.71 | 0.73 |
| 109 | 113.41 | 33.21 | 5.53 | 1.69 | 0.98 | 9.48 | 6.55 | 2.09 | 0.93 | 0.68 |
| 110 | 185.45 | 62.97 | 10.15 | 4.74 | 1.91 | 17.40 | 12.93 | 3.84 | 2.57 | 1.32 |

Raw data of root diameter classes (Total Root volume and Total Root Tips) under P + conditions:

|  | Total Root volume | | | | | Total Root Tips | | | | |
| --- | --- | --- | --- | --- | --- | --- | --- | --- | --- | --- |
| Genotypes | TRV1 | TRV2 | TRV3 | TRV4 | TRV5 | TRT1 | TRT2 | TRT3 | TRT4 | TRT5 |
| 1 | 0.41 | 0.50 | 0.16 | 0.15 | 0.08 | 922.33 | 18.67 | 1.67 | 0.67 | 0.33 |
| 2 | 0.40 | 0.23 | 0.12 | 0.04 | 0.05 | 1063.33 | 28.00 | 1.00 | 0.67 | 0.33 |
| 3 | 0.33 | 0.32 | 0.10 | 0.09 | 0.07 | 1127.33 | 26.33 | 1.33 | 0.33 | 0.33 |
| 4 | 0.45 | 0.40 | 0.15 | 0.13 | 0.05 | 1654.67 | 43.33 | 3.00 | 2.33 | 0.33 |
| 5 | 0.24 | 0.21 | 0.11 | 0.09 | 0.06 | 1042.67 | 37.00 | 4.67 | 0.67 | 0.33 |
| 6 | 0.29 | 0.16 | 0.10 | 0.04 | 0.02 | 1201.67 | 29.67 | 2.33 | 0.33 | 0.00 |
| 7 | 0.33 | 0.36 | 0.15 | 0.14 | 0.16 | 474.00 | 10.33 | 1.00 | 0.00 | 0.00 |
| 8 | 0.26 | 0.18 | 0.12 | 0.09 | 0.06 | 589.33 | 5.00 | 2.00 | 1.00 | 0.00 |
| 9 | 0.30 | 0.35 | 0.13 | 0.13 | 0.11 | 801.67 | 13.67 | 1.00 | 0.00 | 0.33 |
| 10 | 0.25 | 0.19 | 0.07 | 0.05 | 0.02 | 833.00 | 20.33 | 0.67 | 0.67 | 0.00 |
| 11 | 0.13 | 0.09 | 0.02 | 0.00 | 0.00 | 248.00 | 8.00 | 0.00 | 0.00 | 0.00 |
| 12 | 0.09 | 0.04 | 0.01 | 0.01 | 0.01 | 300.00 | 7.33 | 0.33 | 0.00 | 0.00 |
| 13 | 0.25 | 0.23 | 0.11 | 0.12 | 0.05 | 577.00 | 12.33 | 0.33 | 0.67 | 0.00 |
| 14 | 0.22 | 0.26 | 0.12 | 0.08 | 0.05 | 728.00 | 13.00 | 0.33 | 0.33 | 0.00 |
| 15 | 0.18 | 0.13 | 0.09 | 0.05 | 0.02 | 679.00 | 10.33 | 1.00 | 0.33 | 0.00 |
| 16 | 0.16 | 0.15 | 0.07 | 0.04 | 0.02 | 425.67 | 4.67 | 1.00 | 0.00 | 0.00 |
| 17 | 0.29 | 0.31 | 0.11 | 0.10 | 0.06 | 305.00 | 6.33 | 0.00 | 1.00 | 0.00 |
| 18 | 0.19 | 0.15 | 0.06 | 0.05 | 0.05 | 334.00 | 7.33 | 1.00 | 0.67 | 0.00 |
| 19 | 0.22 | 0.19 | 0.10 | 0.11 | 0.05 | 516.67 | 5.33 | 0.67 | 0.00 | 0.00 |
| 20 | 0.15 | 0.15 | 0.07 | 0.04 | 0.01 | 358.33 | 4.67 | 1.00 | 0.00 | 0.00 |
| 21 | 0.17 | 0.18 | 0.06 | 0.02 | 0.01 | 249.00 | 5.00 | 1.00 | 0.00 | 0.00 |
| 22 | 0.24 | 0.20 | 0.08 | 0.05 | 0.05 | 313.00 | 6.33 | 0.67 | 0.33 | 0.33 |
| 23 | 0.20 | 0.25 | 0.13 | 0.12 | 0.07 | 372.67 | 9.00 | 0.67 | 1.00 | 0.00 |
| 24 | 0.36 | 0.29 | 0.11 | 0.12 | 0.04 | 384.33 | 7.67 | 0.00 | 1.00 | 0.67 |
| 25 | 0.12 | 0.19 | 0.09 | 0.08 | 0.03 | 232.67 | 4.67 | 1.00 | 1.00 | 0.33 |
| 26 | 0.21 | 0.18 | 0.07 | 0.07 | 0.03 | 277.67 | 4.67 | 1.33 | 0.67 | 0.00 |
| 27 | 0.18 | 0.25 | 0.10 | 0.07 | 0.04 | 285.00 | 6.33 | 0.67 | 0.00 | 0.67 |
| 28 | 0.25 | 0.25 | 0.08 | 0.13 | 0.06 | 329.00 | 3.33 | 1.00 | 0.67 | 0.33 |
| 29 | 0.14 | 0.15 | 0.10 | 0.03 | 0.02 | 217.67 | 3.33 | 1.00 | 0.33 | 0.00 |
| 30 | 0.21 | 0.25 | 0.13 | 0.12 | 0.08 | 280.33 | 4.00 | 1.00 | 0.00 | 0.00 |
| 31 | 0.23 | 0.19 | 0.12 | 0.03 | 0.06 | 241.33 | 6.00 | 1.00 | 0.67 | 0.67 |
| 32 | 0.20 | 0.18 | 0.10 | 0.06 | 0.04 | 307.33 | 5.33 | 0.67 | 0.33 | 0.33 |
| 33 | 0.25 | 0.28 | 0.14 | 0.10 | 0.05 | 361.33 | 10.00 | 0.67 | 0.67 | 0.33 |
| 34 | 0.16 | 0.16 | 0.08 | 0.05 | 0.01 | 232.00 | 6.00 | 1.00 | 0.33 | 0.33 |
| 35 | 0.24 | 0.24 | 0.14 | 0.10 | 0.08 | 301.00 | 10.67 | 1.33 | 0.33 | 0.33 |
| 36 | 0.17 | 0.25 | 0.10 | 0.10 | 0.06 | 369.67 | 9.33 | 0.67 | 0.67 | 0.00 |
| 37 | 0.22 | 0.30 | 0.17 | 0.17 | 0.12 | 495.33 | 11.33 | 0.67 | 0.33 | 0.00 |
| 38 | 0.32 | 0.38 | 0.18 | 0.11 | 0.08 | 513.33 | 12.33 | 2.00 | 0.00 | 0.00 |
| 39 | 0.20 | 0.22 | 0.13 | 0.09 | 0.04 | 460.00 | 10.00 | 0.67 | 0.00 | 0.00 |
| 40 | 0.27 | 0.33 | 0.18 | 0.16 | 0.08 | 544.00 | 11.00 | 0.33 | 0.67 | 0.00 |
| 41 | 0.15 | 0.30 | 0.15 | 0.10 | 0.10 | 423.67 | 9.00 | 0.67 | 0.00 | 0.00 |
| 42 | 0.23 | 0.27 | 0.12 | 0.11 | 0.08 | 345.00 | 6.33 | 0.67 | 0.33 | 0.33 |
| 43 | 0.13 | 0.13 | 0.06 | 0.03 | 0.02 | 283.67 | 6.33 | 0.67 | 0.33 | 0.33 |
| 44 | 0.16 | 0.18 | 0.13 | 0.11 | 0.09 | 302.00 | 4.00 | 0.33 | 0.33 | 0.00 |
| 45 | 0.17 | 0.27 | 0.16 | 0.13 | 0.09 | 229.00 | 7.67 | 0.67 | 0.67 | 0.67 |
| 46 | 0.16 | 0.24 | 0.18 | 0.11 | 0.03 | 261.00 | 4.67 | 1.33 | 0.00 | 0.00 |
| 47 | 0.15 | 0.17 | 0.07 | 0.07 | 0.05 | 268.33 | 4.67 | 0.33 | 0.33 | 0.00 |
| 48 | 0.20 | 0.28 | 0.23 | 0.22 | 0.13 | 497.00 | 8.67 | 2.33 | 0.33 | 0.00 |
| 49 | 0.10 | 0.18 | 0.11 | 0.10 | 0.09 | 327.67 | 7.33 | 0.67 | 0.33 | 0.00 |
| 50 | 0.13 | 0.27 | 0.16 | 0.18 | 0.10 | 394.33 | 9.33 | 1.33 | 0.33 | 0.33 |
| 51 | 0.09 | 0.17 | 0.11 | 0.12 | 0.06 | 144.33 | 3.33 | 0.67 | 0.00 | 0.00 |
| 52 | 0.12 | 0.17 | 0.11 | 0.12 | 0.12 | 249.00 | 5.33 | 0.67 | 0.33 | 0.00 |
| 53 | 0.13 | 0.25 | 0.15 | 0.17 | 0.17 | 356.67 | 10.00 | 0.00 | 0.67 | 0.00 |
| 54 | 0.12 | 0.19 | 0.14 | 0.13 | 0.10 | 334.33 | 8.33 | 1.33 | 1.00 | 0.67 |
| 55 | 0.07 | 0.13 | 0.11 | 0.08 | 0.10 | 128.67 | 3.67 | 0.67 | 0.33 | 0.33 |
| 56 | 0.13 | 0.21 | 0.14 | 0.15 | 0.10 | 250.67 | 6.00 | 0.67 | 1.33 | 0.00 |
| 57 | 0.11 | 0.20 | 0.16 | 0.15 | 0.16 | 304.00 | 8.67 | 1.67 | 1.00 | 0.33 |
| 58 | 0.20 | 0.33 | 0.25 | 0.27 | 0.22 | 401.33 | 8.33 | 1.00 | 0.00 | 0.67 |
| 59 | 0.11 | 0.13 | 0.09 | 0.10 | 0.04 | 300.33 | 3.67 | 0.00 | 0.33 | 0.00 |
| 60 | 0.14 | 0.27 | 0.15 | 0.15 | 0.19 | 428.00 | 9.33 | 0.33 | 0.67 | 0.00 |
| 61 | 0.03 | 0.04 | 0.02 | 0.01 | 0.01 | 64.33 | 1.67 | 0.67 | 0.00 | 0.00 |
| 62 | 0.18 | 0.25 | 0.15 | 0.14 | 0.12 | 251.33 | 8.33 | 1.67 | 0.33 | 0.33 |
| 63 | 0.19 | 0.24 | 0.15 | 0.13 | 0.10 | 296.67 | 7.00 | 1.33 | 0.00 | 1.00 |
| 64 | 0.23 | 0.37 | 0.24 | 0.20 | 0.18 | 536.67 | 15.33 | 3.00 | 0.33 | 0.67 |
| 65 | 0.10 | 0.12 | 0.07 | 0.07 | 0.03 | 112.00 | 4.33 | 0.67 | 0.33 | 0.00 |
| 66 | 0.13 | 0.24 | 0.18 | 0.15 | 0.13 | 176.67 | 5.00 | 1.67 | 0.00 | 1.00 |
| 67 | 0.09 | 0.14 | 0.12 | 0.13 | 0.12 | 156.00 | 6.00 | 1.00 | 0.33 | 0.00 |
| 68 | 0.16 | 0.26 | 0.13 | 0.12 | 0.07 | 220.67 | 5.33 | 0.33 | 0.67 | 0.00 |
| 69 | 0.13 | 0.24 | 0.16 | 0.16 | 0.09 | 216.67 | 5.00 | 0.67 | 0.67 | 0.33 |
| 70 | 0.11 | 0.15 | 0.10 | 0.08 | 0.04 | 243.33 | 5.33 | 0.33 | 0.67 | 0.33 |
| 71 | 0.15 | 0.33 | 0.19 | 0.23 | 0.14 | 489.00 | 15.00 | 1.67 | 0.67 | 0.00 |
| 72 | 0.07 | 0.10 | 0.04 | 0.02 | 0.02 | 56.00 | 4.67 | 0.67 | 0.33 | 0.00 |
| 73 | 0.09 | 0.10 | 0.04 | 0.01 | 0.00 | 120.67 | 5.33 | 0.67 | 0.67 | 0.00 |
| 74 | 0.13 | 0.18 | 0.11 | 0.09 | 0.07 | 316.33 | 7.00 | 0.67 | 0.33 | 0.00 |
| 75 | 0.10 | 0.14 | 0.07 | 0.05 | 0.04 | 252.33 | 9.33 | 0.33 | 0.00 | 0.67 |
| 76 | 0.09 | 0.12 | 0.06 | 0.05 | 0.03 | 291.33 | 6.67 | 0.00 | 1.00 | 0.00 |
| 77 | 0.08 | 0.11 | 0.06 | 0.04 | 0.03 | 226.67 | 7.33 | 0.33 | 0.00 | 0.00 |
| 78 | 0.18 | 0.26 | 0.14 | 0.11 | 0.09 | 365.67 | 11.33 | 1.67 | 0.33 | 0.67 |
| 79 | 0.09 | 0.13 | 0.10 | 0.09 | 0.07 | 116.00 | 5.33 | 0.67 | 0.00 | 0.33 |
| 80 | 0.15 | 0.25 | 0.13 | 0.12 | 0.08 | 220.00 | 5.67 | 1.00 | 0.00 | 0.00 |
| 81 | 0.13 | 0.23 | 0.17 | 0.16 | 0.09 | 224.00 | 4.67 | 0.33 | 1.00 | 0.33 |
| 82 | 0.11 | 0.20 | 0.09 | 0.08 | 0.04 | 264.00 | 8.67 | 0.33 | 0.33 | 0.33 |
| 83 | 0.16 | 0.29 | 0.19 | 0.23 | 0.13 | 444.00 | 13.33 | 1.33 | 1.00 | 0.00 |
| 84 | 0.05 | 0.08 | 0.03 | 0.00 | 0.01 | 52.33 | 3.67 | 1.00 | 0.33 | 0.00 |
| 85 | 0.17 | 0.21 | 0.13 | 0.09 | 0.07 | 321.00 | 9.00 | 0.67 | 0.33 | 0.00 |
| 86 | 0.05 | 0.07 | 0.02 | 0.01 | 0.01 | 154.67 | 3.67 | 0.33 | 0.33 | 0.00 |
| 87 | 0.11 | 0.15 | 0.09 | 0.06 | 0.06 | 273.33 | 9.00 | 0.33 | 0.67 | 0.67 |
| 88 | 0.09 | 0.10 | 0.06 | 0.04 | 0.02 | 285.00 | 7.33 | 0.33 | 0.33 | 0.00 |
| 89 | 0.22 | 0.19 | 0.09 | 0.06 | 0.05 | 821.67 | 17.33 | 1.00 | 0.33 | 0.33 |
| 90 | 0.41 | 0.42 | 0.16 | 0.15 | 0.06 | 1450.67 | 46.67 | 4.33 | 2.33 | 0.33 |
| 91 | 0.23 | 0.16 | 0.08 | 0.07 | 0.04 | 862.67 | 30.00 | 2.67 | 0.67 | 0.00 |
| 92 | 0.27 | 0.20 | 0.10 | 0.06 | 0.05 | 1025.33 | 23.33 | 2.00 | 0.00 | 0.00 |
| 93 | 0.35 | 0.31 | 0.15 | 0.11 | 0.13 | 624.33 | 11.33 | 1.33 | 0.33 | 0.00 |
| 94 | 0.11 | 0.15 | 0.09 | 0.06 | 0.05 | 172.67 | 3.67 | 0.33 | 0.00 | 0.00 |
| 95 | 0.12 | 0.27 | 0.17 | 0.23 | 0.24 | 395.67 | 9.33 | 0.33 | 1.00 | 0.00 |
| 96 | 0.13 | 0.21 | 0.15 | 0.12 | 0.09 | 312.67 | 10.00 | 1.33 | 0.33 | 0.00 |
| 97 | 0.04 | 0.06 | 0.06 | 0.07 | 0.07 | 136.33 | 2.33 | 0.33 | 1.00 | 0.67 |
| 98 | 0.17 | 0.22 | 0.13 | 0.10 | 0.09 | 313.67 | 6.00 | 0.67 | 0.67 | 0.33 |
| 99 | 0.22 | 0.25 | 0.13 | 0.08 | 0.05 | 819.67 | 13.67 | 1.00 | 0.00 | 0.00 |
| 100 | 0.15 | 0.10 | 0.07 | 0.03 | 0.01 | 524.67 | 8.33 | 0.67 | 0.33 | 0.00 |
| 101 | 0.18 | 0.21 | 0.08 | 0.06 | 0.04 | 348.33 | 3.67 | 0.67 | 0.33 | 0.00 |
| 102 | 0.26 | 0.26 | 0.09 | 0.08 | 0.06 | 300.33 | 7.33 | 0.67 | 1.00 | 0.00 |
| 103 | 0.23 | 0.19 | 0.09 | 0.11 | 0.06 | 440.33 | 8.00 | 0.67 | 0.33 | 0.00 |
| 104 | 0.09 | 0.07 | 0.05 | 0.07 | 0.07 | 258.33 | 2.00 | 0.67 | 1.00 | 0.67 |
| 105 | 0.08 | 0.17 | 0.12 | 0.09 | 0.10 | 128.67 | 3.67 | 0.67 | 0.33 | 0.33 |
| 106 | 0.14 | 0.19 | 0.13 | 0.13 | 0.08 | 268.67 | 6.67 | 0.67 | 1.00 | 0.00 |
| 107 | 0.13 | 0.24 | 0.18 | 0.20 | 0.21 | 335.00 | 9.67 | 1.67 | 1.00 | 0.33 |
| 108 | 0.18 | 0.28 | 0.22 | 0.22 | 0.17 | 400.67 | 9.33 | 0.67 | 0.00 | 0.67 |
| 109 | 0.15 | 0.13 | 0.05 | 0.07 | 0.02 | 427.67 | 9.33 | 0.67 | 0.33 | 0.00 |
| 110 | 0.26 | 0.21 | 0.11 | 0.09 | 0.05 | 592.00 | 10.67 | 0.00 | 0.67 | 0.00 |

Raw data of root diameter classes (Total Root volume and Total Root Tips) under P - conditions:

|  | TRV | | | | | TRT | | | | |
| --- | --- | --- | --- | --- | --- | --- | --- | --- | --- | --- |
| Genotypes | TRV1 | TRV2 | TRV3 | TRV4 | TRV5 | TRT1 | TRT2 | TRT3 | TRT4 | TRT5 |
| 1 | 0.22 | 0.18 | 0.10 | 0.04 | 0.02 | 572.00 | 12.00 | 3.33 | 0.33 | 0.00 |
| 2 | 0.29 | 0.22 | 0.08 | 0.03 | 0.02 | 367.67 | 10.33 | 1.33 | 1.00 | 0.00 |
| 3 | 0.17 | 0.12 | 0.05 | 0.04 | 0.01 | 310.33 | 4.67 | 0.67 | 1.33 | 0.00 |
| 4 | 0.24 | 0.21 | 0.08 | 0.05 | 0.02 | 359.33 | 6.67 | 1.00 | 0.67 | 0.33 |
| 5 | 0.32 | 0.36 | 0.16 | 0.13 | 0.06 | 623.00 | 12.33 | 1.67 | 0.33 | 0.00 |
| 6 | 0.25 | 0.18 | 0.10 | 0.06 | 0.04 | 490.33 | 6.67 | 0.00 | 0.33 | 0.67 |
| 7 | 0.34 | 0.27 | 0.14 | 0.11 | 0.07 | 568.00 | 11.33 | 0.67 | 0.00 | 0.00 |
| 8 | 0.13 | 0.12 | 0.05 | 0.04 | 0.03 | 416.67 | 6.67 | 1.00 | 0.67 | 0.00 |
| 9 | 0.27 | 0.17 | 0.10 | 0.07 | 0.02 | 1520.33 | 15.67 | 1.00 | 1.33 | 0.33 |
| 10 | 0.17 | 0.13 | 0.05 | 0.06 | 0.04 | 792.00 | 11.67 | 2.00 | 0.33 | 0.00 |
| 11 | 0.31 | 0.32 | 0.14 | 0.04 | 0.02 | 804.67 | 11.33 | 3.33 | 0.33 | 0.00 |
| 12 | 0.10 | 0.09 | 0.05 | 0.06 | 0.04 | 227.00 | 3.67 | 0.00 | 1.33 | 0.00 |
| 13 | 0.15 | 0.08 | 0.04 | 0.03 | 0.02 | 412.00 | 4.00 | 1.00 | 0.00 | 0.67 |
| 14 | 0.16 | 0.09 | 0.05 | 0.03 | 0.02 | 389.33 | 6.00 | 0.67 | 1.00 | 0.00 |
| 15 | 0.19 | 0.12 | 0.05 | 0.03 | 0.02 | 461.00 | 6.67 | 1.00 | 0.33 | 0.00 |
| 16 | 0.15 | 0.14 | 0.06 | 0.05 | 0.06 | 213.00 | 5.67 | 0.00 | 0.00 | 0.00 |
| 17 | 0.19 | 0.27 | 0.09 | 0.06 | 0.07 | 553.67 | 12.33 | 1.67 | 0.00 | 0.00 |
| 18 | 0.13 | 0.05 | 0.03 | 0.02 | 0.03 | 307.33 | 8.33 | 1.33 | 0.00 | 0.33 |
| 19 | 0.15 | 0.14 | 0.04 | 0.04 | 0.03 | 366.33 | 5.67 | 0.33 | 0.67 | 0.33 |
| 20 | 0.17 | 0.21 | 0.07 | 0.08 | 0.06 | 567.33 | 12.00 | 0.67 | 1.00 | 0.33 |
| 21 | 0.21 | 0.25 | 0.13 | 0.11 | 0.05 | 272.67 | 6.67 | 1.67 | 0.33 | 0.33 |
| 22 | 0.19 | 0.22 | 0.08 | 0.06 | 0.05 | 205.33 | 7.67 | 1.00 | 0.00 | 0.00 |
| 23 | 0.16 | 0.32 | 0.17 | 0.15 | 0.16 | 250.33 | 8.33 | 1.67 | 1.00 | 0.00 |
| 24 | 0.18 | 0.30 | 0.18 | 0.16 | 0.11 | 225.00 | 6.33 | 2.00 | 0.33 | 0.00 |
| 25 | 0.14 | 0.21 | 0.11 | 0.11 | 0.10 | 224.67 | 7.00 | 1.33 | 0.00 | 0.33 |
| 26 | 0.18 | 0.25 | 0.12 | 0.10 | 0.08 | 260.33 | 4.67 | 2.00 | 0.33 | 0.33 |
| 27 | 0.15 | 0.23 | 0.13 | 0.09 | 0.06 | 249.00 | 5.67 | 1.00 | 0.33 | 0.00 |
| 28 | 0.11 | 0.14 | 0.08 | 0.06 | 0.03 | 162.33 | 3.00 | 1.33 | 0.67 | 0.00 |
| 29 | 0.14 | 0.18 | 0.11 | 0.07 | 0.05 | 156.00 | 5.67 | 0.00 | 0.67 | 0.00 |
| 30 | 0.14 | 0.36 | 0.23 | 0.20 | 0.17 | 197.33 | 11.00 | 1.00 | 0.67 | 0.67 |
| 31 | 0.12 | 0.13 | 0.07 | 0.05 | 0.04 | 91.33 | 4.67 | 0.67 | 0.67 | 0.33 |
| 32 | 0.10 | 0.20 | 0.11 | 0.11 | 0.09 | 181.67 | 7.00 | 0.67 | 0.33 | 0.33 |
| 33 | 0.09 | 0.15 | 0.06 | 0.06 | 0.03 | 158.67 | 4.67 | 1.00 | 0.67 | 0.33 |
| 34 | 0.10 | 0.11 | 0.04 | 0.04 | 0.02 | 180.33 | 3.67 | 0.00 | 0.00 | 0.67 |
| 35 | 0.12 | 0.14 | 0.09 | 0.08 | 0.04 | 246.67 | 3.67 | 1.00 | 0.33 | 0.33 |
| 36 | 0.19 | 0.24 | 0.11 | 0.09 | 0.03 | 355.33 | 11.33 | 1.33 | 0.33 | 0.00 |
| 37 | 0.16 | 0.17 | 0.09 | 0.06 | 0.04 | 314.67 | 9.33 | 2.00 | 0.00 | 0.00 |
| 38 | 0.17 | 0.21 | 0.12 | 0.08 | 0.05 | 342.67 | 8.67 | 1.33 | 0.00 | 0.00 |
| 39 | 0.16 | 0.16 | 0.10 | 0.08 | 0.04 | 264.33 | 4.33 | 1.00 | 0.00 | 0.00 |
| 40 | 0.07 | 0.09 | 0.05 | 0.05 | 0.01 | 152.33 | 4.33 | 0.00 | 0.00 | 0.00 |
| 41 | 0.12 | 0.08 | 0.04 | 0.03 | 0.02 | 200.33 | 3.00 | 0.00 | 0.33 | 0.00 |
| 42 | 0.13 | 0.13 | 0.09 | 0.08 | 0.04 | 339.00 | 7.67 | 0.33 | 0.33 | 0.00 |
| 43 | 0.09 | 0.08 | 0.04 | 0.01 | 0.00 | 194.67 | 3.67 | 0.33 | 0.33 | 0.00 |
| 44 | 0.13 | 0.15 | 0.09 | 0.04 | 0.02 | 154.33 | 3.67 | 1.00 | 0.33 | 0.00 |
| 45 | 0.21 | 0.16 | 0.09 | 0.06 | 0.02 | 236.67 | 5.33 | 0.67 | 1.00 | 0.00 |
| 46 | 0.13 | 0.12 | 0.10 | 0.04 | 0.02 | 171.67 | 4.67 | 0.67 | 0.33 | 0.00 |
| 47 | 0.14 | 0.19 | 0.12 | 0.06 | 0.03 | 177.67 | 4.67 | 0.67 | 0.00 | 0.00 |
| 48 | 0.21 | 0.26 | 0.10 | 0.07 | 0.06 | 264.00 | 12.00 | 0.33 | 0.00 | 0.00 |
| 49 | 0.15 | 0.22 | 0.12 | 0.10 | 0.06 | 225.33 | 3.67 | 0.67 | 0.33 | 0.33 |
| 50 | 0.09 | 0.18 | 0.10 | 0.09 | 0.04 | 235.33 | 5.33 | 0.67 | 0.33 | 0.33 |
| 51 | 0.11 | 0.16 | 0.11 | 0.08 | 0.05 | 270.33 | 3.67 | 1.00 | 0.67 | 0.33 |
| 52 | 0.05 | 0.08 | 0.03 | 0.01 | 0.01 | 162.33 | 3.33 | 1.00 | 0.33 | 0.33 |
| 53 | 0.20 | 0.32 | 0.16 | 0.15 | 0.09 | 313.33 | 8.33 | 1.33 | 0.33 | 0.67 |
| 54 | 0.14 | 0.17 | 0.09 | 0.10 | 0.07 | 272.00 | 3.00 | 1.67 | 0.67 | 0.33 |
| 55 | 0.20 | 0.27 | 0.19 | 0.17 | 0.11 | 511.67 | 9.67 | 1.00 | 0.67 | 0.00 |
| 56 | 0.20 | 0.28 | 0.14 | 0.11 | 0.06 | 513.33 | 11.00 | 2.33 | 1.00 | 0.00 |
| 57 | 0.12 | 0.13 | 0.08 | 0.04 | 0.02 | 97.33 | 2.00 | 0.67 | 0.00 | 0.00 |
| 58 | 0.24 | 0.27 | 0.14 | 0.13 | 0.08 | 192.33 | 10.00 | 0.67 | 0.67 | 0.00 |
| 59 | 0.16 | 0.22 | 0.11 | 0.09 | 0.04 | 175.00 | 2.67 | 1.33 | 0.33 | 0.00 |
| 60 | 0.11 | 0.21 | 0.10 | 0.11 | 0.09 | 173.67 | 5.67 | 0.67 | 1.33 | 0.00 |
| 61 | 0.04 | 0.02 | 0.02 | 0.02 | 0.03 | 124.00 | 1.67 | 0.33 | 0.00 | 1.00 |
| 62 | 0.16 | 0.22 | 0.16 | 0.15 | 0.11 | 217.67 | 7.00 | 0.33 | 0.67 | 0.00 |
| 63 | 0.12 | 0.16 | 0.11 | 0.09 | 0.07 | 249.00 | 5.67 | 1.67 | 0.00 | 0.00 |
| 64 | 0.15 | 0.21 | 0.14 | 0.12 | 0.11 | 330.33 | 4.33 | 0.67 | 0.33 | 0.00 |
| 65 | 0.20 | 0.32 | 0.18 | 0.16 | 0.14 | 552.00 | 12.67 | 1.00 | 0.67 | 0.33 |
| 66 | 0.16 | 0.18 | 0.15 | 0.10 | 0.05 | 466.67 | 6.33 | 0.00 | 0.67 | 0.00 |
| 67 | 0.16 | 0.46 | 0.24 | 0.21 | 0.12 | 503.67 | 9.33 | 0.33 | 0.00 | 0.33 |
| 68 | 0.11 | 0.20 | 0.11 | 0.08 | 0.07 | 308.00 | 5.67 | 1.33 | 0.00 | 1.00 |
| 69 | 0.08 | 0.10 | 0.05 | 0.05 | 0.04 | 305.67 | 6.00 | 0.00 | 1.00 | 0.00 |
| 70 | 0.10 | 0.11 | 0.08 | 0.04 | 0.03 | 271.67 | 6.67 | 1.33 | 0.33 | 0.00 |
| 71 | 0.07 | 0.09 | 0.03 | 0.01 | 0.01 | 77.33 | 1.00 | 0.67 | 0.00 | 0.00 |
| 72 | 0.08 | 0.09 | 0.05 | 0.02 | 0.00 | 115.33 | 1.67 | 0.67 | 0.67 | 0.00 |
| 73 | 0.06 | 0.08 | 0.04 | 0.03 | 0.01 | 83.00 | 2.00 | 0.67 | 0.33 | 0.00 |
| 74 | 0.04 | 0.07 | 0.03 | 0.01 | 0.00 | 76.67 | 2.33 | 0.33 | 0.33 | 0.00 |
| 75 | 0.06 | 0.12 | 0.06 | 0.01 | 0.01 | 130.00 | 3.00 | 0.33 | 0.00 | 0.00 |
| 76 | 0.03 | 0.05 | 0.01 | 0.00 | 0.00 | 81.00 | 2.67 | 1.67 | 0.00 | 0.00 |
| 77 | 0.07 | 0.07 | 0.01 | 0.01 | 0.02 | 129.67 | 2.33 | 0.67 | 0.00 | 0.33 |
| 78 | 0.25 | 0.19 | 0.06 | 0.03 | 0.02 | 408.33 | 10.67 | 1.33 | 1.00 | 0.00 |
| 79 | 0.26 | 0.17 | 0.08 | 0.04 | 0.01 | 378.67 | 8.00 | 1.00 | 1.00 | 0.00 |
| 80 | 0.20 | 0.18 | 0.06 | 0.04 | 0.01 | 363.33 | 6.67 | 0.33 | 1.00 | 0.00 |
| 81 | 0.20 | 0.26 | 0.12 | 0.09 | 0.06 | 453.33 | 6.33 | 0.67 | 0.00 | 0.33 |
| 82 | 0.26 | 0.24 | 0.11 | 0.07 | 0.02 | 490.00 | 10.00 | 1.67 | 0.33 | 0.67 |
| 83 | 0.35 | 0.23 | 0.15 | 0.12 | 0.09 | 527.67 | 9.33 | 0.00 | 0.33 | 0.00 |
| 84 | 0.24 | 0.20 | 0.09 | 0.05 | 0.02 | 493.67 | 7.33 | 1.33 | 0.00 | 0.00 |
| 85 | 0.12 | 0.11 | 0.07 | 0.05 | 0.03 | 565.00 | 11.00 | 1.00 | 1.67 | 0.33 |
| 86 | 0.35 | 0.23 | 0.10 | 0.10 | 0.05 | 1873.67 | 19.67 | 1.00 | 0.67 | 0.00 |
| 87 | 0.21 | 0.20 | 0.09 | 0.03 | 0.01 | 640.67 | 9.67 | 4.33 | 0.00 | 0.00 |
| 88 | 0.21 | 0.21 | 0.10 | 0.06 | 0.05 | 415.00 | 6.00 | 0.33 | 1.67 | 0.00 |
| 89 | 0.11 | 0.08 | 0.02 | 0.03 | 0.02 | 282.33 | 2.67 | 0.33 | 0.00 | 0.00 |
| 90 | 0.16 | 0.08 | 0.06 | 0.03 | 0.02 | 449.00 | 5.33 | 1.33 | 0.67 | 0.67 |
| 91 | 0.21 | 0.12 | 0.06 | 0.04 | 0.04 | 513.67 | 9.00 | 1.00 | 0.67 | 0.00 |
| 92 | 0.13 | 0.11 | 0.04 | 0.03 | 0.04 | 220.00 | 4.00 | 0.00 | 0.00 | 0.00 |
| 93 | 0.16 | 0.22 | 0.09 | 0.07 | 0.06 | 454.67 | 10.00 | 1.33 | 0.00 | 0.00 |
| 94 | 0.17 | 0.14 | 0.05 | 0.03 | 0.04 | 393.00 | 9.33 | 1.33 | 0.00 | 0.00 |
| 95 | 0.12 | 0.08 | 0.04 | 0.05 | 0.04 | 260.33 | 7.67 | 0.67 | 0.33 | 0.67 |
| 96 | 0.10 | 0.11 | 0.08 | 0.04 | 0.03 | 271.67 | 6.67 | 1.33 | 0.33 | 0.00 |
| 97 | 0.07 | 0.09 | 0.03 | 0.01 | 0.01 | 77.33 | 1.00 | 0.67 | 0.00 | 0.00 |
| 98 | 0.08 | 0.09 | 0.05 | 0.02 | 0.00 | 115.33 | 1.67 | 0.67 | 0.67 | 0.00 |
| 99 | 0.06 | 0.08 | 0.04 | 0.03 | 0.01 | 83.00 | 2.00 | 0.67 | 0.33 | 0.00 |
| 100 | 0.04 | 0.07 | 0.03 | 0.01 | 0.00 | 76.67 | 2.33 | 0.33 | 0.33 | 0.00 |
| 101 | 0.06 | 0.12 | 0.06 | 0.01 | 0.01 | 130.00 | 3.00 | 0.33 | 0.00 | 0.00 |
| 102 | 0.03 | 0.05 | 0.01 | 0.00 | 0.00 | 81.00 | 2.67 | 1.67 | 0.00 | 0.00 |
| 103 | 0.07 | 0.07 | 0.01 | 0.01 | 0.02 | 129.67 | 2.33 | 0.67 | 0.00 | 0.33 |
| 104 | 0.13 | 0.14 | 0.08 | 0.04 | 0.01 | 281.67 | 7.33 | 0.67 | 0.00 | 0.00 |
| 105 | 0.21 | 0.29 | 0.13 | 0.08 | 0.07 | 264.67 | 9.00 | 0.67 | 0.00 | 0.00 |
| 106 | 0.14 | 0.19 | 0.10 | 0.07 | 0.04 | 204.33 | 7.33 | 0.33 | 0.00 | 0.33 |
| 107 | 0.15 | 0.24 | 0.13 | 0.11 | 0.07 | 279.00 | 5.00 | 0.67 | 0.67 | 0.00 |
| 108 | 0.10 | 0.13 | 0.08 | 0.07 | 0.04 | 225.33 | 3.67 | 0.67 | 0.33 | 0.67 |
| 109 | 0.07 | 0.11 | 0.06 | 0.04 | 0.04 | 199.67 | 2.67 | 0.67 | 0.67 | 0.33 |
| 110 | 0.15 | 0.22 | 0.12 | 0.11 | 0.07 | 256.00 | 7.00 | 2.00 | 0.33 | 0.33 |
